# DARE Training: Teaching Educators How to Revise Internal Medicine Residency Lectures by Using an Anti-racism Framework

**DOI:** 10.15766/mep_2374-8265.11351

**Published:** 2023-11-07

**Authors:** Amanda R. Jowell, Aisha K. James, Rashmi Jasrasaria, Michael S. Kelly, Madeleine I. Matthiesen, Darshali A. Vyas, Sherri-Ann M. Burnett-Bowie, Jessica A. Zeidman

**Affiliations:** 1 First-Year Resident, Department of Medicine, Massachusetts General Hospital; 2 Primary Care Physician and Director for Racial Justice, Department of Medicine, and Primary Care Physician and Associate Director for the Diversity, Equity and Inclusion Committee, Department of Pediatrics, Massachusetts General Hospital for Children; Instructor in Medicine, Harvard Medical School; 3 Primary Care Physician, Department of Medicine, and Associate Director, Center for Immigrant Health, Massachusetts General Hospital; Instructor in Medicine, Harvard Medical School; 4 Pulmonary and Critical Care Fellow, Department of Medicine, Massachusetts General Hospital and Beth Israel Deaconess Medical Center; 5 Hospitalist, Departments of Medicine and Pediatrics, Core Educator Faculty, Department of Medicine, Associate Program Director, Internal Medicine and Pediatrics Residency Program, Massachusetts General Hospital for Children; Instructor in Medicine, Harvard Medical School; 6 Endocrinologist, Department of Medicine, Massachusetts General Hospital; Associate Professor of Medicine, Harvard Medical School; Associate Director, Massachusetts General Center for Diversity and Inclusion, Massachusetts General Hospital; and Chair, Diversity and Inclusion Board, Department of Medicine, Massachusetts General Hospital; 7 Primary Care Physician and Primary Care Program Director, Department of Medicine, Massachusetts General Hospital; Instructor in Medicine, Harvard Medical School; † Co-senior author

**Keywords:** Case-Based Learning, Curriculum Development, Health Equity, Internal Medicine, Self-Assessment, Anti-racism, Diversity, Equity, Inclusion

## Abstract

**Introduction:**

Systemic inequities and provider-held biases reinforce racism and further disparities in graduate medical education. We developed the Department of Medicine Anti-Racism and Equity Educational Initiative (DARE) to improve internal medicine residency conferences. We trained faculty and residents to serve as coaches to support other faculty in delivering lectures. The training leveraged a best-practices checklist to revise existing lectures.

**Methods:**

We recruited internal medicine faculty and residents to serve as DARE coaches, who supported educators in improving lectures’ anti-racism content. During the training, coaches watched a videotaped didactic presentation that we created about health equity and anti-racism frameworks. DARE coaches then participated in a workshop where they engaged in case-based learning and small-group discussion to apply the DARE best-practices checklist to sample lecture slides. To assess training effectiveness, coaches completed pre- and posttraining assessments in which they edited different sample lecture slides. Our training took 1 hour to complete.

**Results:**

Thirty-four individuals completed DARE training. Following the training, the sample slides were significantly improved with respect to diversity of graphics (*p* < .001), discussion of research participant demographics (*p* < .001), and discussion of the impact of racism/bias on health disparities (*p* = .03). After DARE training, 23 of 24 participants (96%) endorsed feeling more prepared to bring an anti-racist framework to lectures and to support colleagues in doing the same.

**Discussion:**

Training residents and faculty to use DARE principles in delivering internal medicine lectures is an innovative and effective way to integrate anti-racism into internal medicine residency conferences.

## Educational Objectives

By the end of this activity, learners will be able to:

1. Define racism and anti-racism.
2. Identify lecture characteristics that perpetuate racism and other forms of bias (e.g., lack of diverse representation in images, use of cases or images that confirm historical stereotypes, use of out-of-date terminology, promotion of clinical decision tools that conflate race with genetics).
3. Describe strategies to reduce bias in lecture content and increase information about racism's impact on health in lectures.
4. Apply a best-practices checklist to revise and teach medical curricula using an anti-racist and equity framework.

## Introduction

Racism is a public health crisis, and structural, institutional, interpersonal, and internalized racism worsen health outcomes.^1–7^ To ensure that physicians have the knowledge and tools to address racism and care for diverse communities, medical students, residents, educators, and national organizations have called for an examination of how race and racism are addressed in medical education.^8,9^

Curricula that educate medical trainees about racism and other forms of bias and discrimination can advance health care quality.^5,10,11^ To date, the majority of published models or interventions for improving discussions of racism and health inequities are discrete preclinical medical school curricula,^12–21^ including lecture series,^13,18,20^ case-based workshops or interactive group sessions,^12,14–17^ and multidisciplinary clinic-based projects.^19^ Lectures may occur once^12^ or monthly^13^ and may include topics such as microaggressions,^16^ implicit bias,^13,15^ or the impact of structural racism on social determinants of health.^17–20^

While most interventions focus on delivering new content to medical trainees, there are few peer-reviewed resources to support medical educators in incorporating racism and health inequities content into existing educational curricula—a missed opportunity.^8,18,21–23^ Furthermore, most of these initiatives are implemented in undergraduate medical education (UME) settings, thus creating a gap in advancing education related to equity during residency training. Moreover, graduate medical education (GME) settings present unique challenges. Whereas UME settings often have course directors who design and oversee significant portions of the UME curricula, many GME curricula consist of conference series that are taught by a large and diffuse faculty and do not have course directors. In such settings, many faculty may give only one lecture annually. This structure makes it particularly difficult to implement curricular reform. Furthermore, medical educators report feeling unprepared to revise educational materials and facilitate discussions about racism in medicine.^21–24^

In response to these gaps and challenges, we developed and implemented the Department of Medicine Anti-Racism and Equity Educational Initiative (DARE). In DARE, we trained volunteer residents and faculty to become coaches who can support other medical educators in improving how race and racism are discussed in lectures. DARE coaches use an anti-racist and equity-focused framework to revise lectures before they are delivered. This DARE best-practices framework can be used to develop novel lectures or to revise existing medical education content by analyzing and editing it to align with these best practices. While outside the scope of this publication, after completing this training we paired coaches with internal medicine residency noon conference lecturers to support the lecturers in revising their materials. This publication describes the development, implementation, and evaluation of this novel training session for DARE coaches.

Our initiative draws on Knowles’ self-directed learning framework, in which individuals are self-directed learners internally motivated to pursue and manage their own learning opportunities.^25^ In this setting, our DARE coaches volunteered to be part of the initiative and participated in DARE training on their own time, motivated by their desire to improve their ability to apply health equity and anti-racism frameworks to medical education. Our initiative also derives from Bandura's social cognitive theory, in which learners are motivated to learn from their peers through observing, retaining, and reproducing demonstrated behavior.^26^ In our model, DARE training participants are internally motivated to attend the training and apply the skills modeled by the DARE workshop facilitator and other participants (their peers) to their own medical education practice and their future work as DARE coaches.

## Methods

### Setting

We implemented the DARE training in an internal medicine residency program at a large urban academic medical center. The study authors, who included program leadership, faculty, residents, and a medical student, designed the training. This project was considered exempt by the Massachusetts General Brigham Institutional Review Board.

### Participant Recruitment

We developed our training for internal medicine faculty and PGY 1 and higher residents. We focused on recruiting faculty, as we anticipated that it might be difficult for residents to coach faculty members. The Massachusetts General Hospital Department of Medicine internal medicine residency program director sent emails informing all core internal medicine residency teaching faculty of the DARE initiative. These emails emphasized DARE's importance in upholding the residency's mission of eliminating bias and preparing trainees to advance health equity. Additionally, we solicited feedback and facilitated engagement in the initiative by presenting the DARE project to the residency associate program directors and core faculty as well as Department of Medicine committees focused on education, equity, and community health. We also presented to and solicited feedback about the DARE project from internal medicine residents, and subsequently, we recruited residents who had expressed a specific interest in DARE and/or health equity efforts in medical education. We trained faculty and residents together as the skills were at the appropriate level for both. We did not require coaches to have any prior skill set or experiences outside of a desire to participate and learn.

### Training Description

We conducted a synthetic literature review to identify existing frameworks to guide the creation of bias-free curricula.^27^ Based on our synthetic literature review and the expertise in our group, we created the DARE checklist of best practices for improving anti-racism and equity content in educational conferences (Appendix A). DARE training included a preworkshop videotaped didactic presentation and a virtual synchronous workshop where participants used the DARE checklist to revise educational materials to reduce content perpetuating racism and other forms of bias and to include content on health inequities and the systems propagating them.

We developed a required 13-minute videotaped didactic presentation reviewing key concepts such as definitions of racism, the impact of racism on health, examples of racism in medical education, and strategies to address racism in medical education. Participants were given access to this video 2 weeks before their participation in the virtual synchronous workshop and asked to watch the video at their convenience prior to attending the workshop. Offering this didactic content in videotaped format enabled us to provide flexibility to participants for completing the didactic portion of the training and to use the virtual synchronous workshop time efficiently. For the purposes of this publication, we have converted the 13-minute video into the preworkshop introduction facilitator guide (Appendix B) and preworkshop introduction slides (Appendix C) to enable institutions to adapt this portion of the training. The video also introduced the DARE checklist. An optional, publicly available, 8-minute video, developed by Warren Alpert Medical School educators, was recommended if participants wanted to learn more about common manifestations of racism in curricula prior to the synchronous workshop.^28^

Participants next attended a 30-minute virtual synchronous workshop. One of the authors (Jessica A. Zeidman) facilitated the workshop using the workshop facilitator guide (Appendix D) and accompanying workshop slides (Appendix E). The facilitator guide provided a workshop overview as well as the script that accompanied each of the workshop slides. This facilitator guide was geared toward anyone interested in organizing a DARE training, and no additional training was required prior to conducting the workshop. The workshop used case-based learning and small-group discussion to enable participants to practice applying the checklist to sample internal medicine lecture slides, identify aspects of the sample lecture slides that did not align with the checklist, and practice suggesting edits. Each sample lecture slide was designed to highlight different DARE checklist principles so that participants could practice applying each aspect of the checklist. During the workshop, the facilitator used an anonymous virtual polling platform to facilitate audience participation. For cases 1–3, the facilitator asked participants to use the DARE checklist to answer multiple-choice questions on improving the sample lecture slides’ content. For the last two cases, the facilitator asked participants to use the DARE checklist to provide free-text suggestions on how to improve the sample lecture slides. To reinforce checklist principles, after participants had submitted their responses and viewed those of their peers, the facilitator led a discussion describing the ways varied responses illustrated different elements of the checklist. Following this discussion, the facilitator showed a sample version of a revised lecture slide, created by the authors, illustrating one way the checklist principles could be applied to improve the slide's content.

### Training Evaluation

#### Administration of the assessment

Before starting the preworkshop videotaped didactic presentation, participants completed the pretraining assessment (Appendix F), in which they were asked to provide revisions to a sample PowerPoint slide's content to make it more inclusive and anti-racist. Following completion of the workshop, participants completed the posttraining assessment (Appendix G), where they reviewed and revised a different PowerPoint slide. The desired revisions encompassed the DARE checklist principles. In addition, the posttraining assessment was used to collect participant demographics as well as participants’ perceived preparedness to apply the training.

#### Evaluation of the assessment

The primary outcome was improvement in participants’ ability to apply the DARE checklist to edit existing educational content, which was measured by comparing pre- and postworkshop assessments. Two authors (Madeleine I. Matthiesen and Michael S. Kelly) developed a rubric (Appendix H) to assess how well the DARE checklist principles had been applied to the pre- and postworkshop assessments. The rubric was designed to assess the key aspects of the checklist promoting anti-racism and inclusion. Specifically, the rubric allowed measurement of five broad categories: diversity of photographs, diversity of graphics, discussion of research study population demographics, use of race/ethnicity in case vignettes, and discussion of the impact of racism on health inequities. Four of the five categories were assessed on a 3-point scale (−1 = *perpetuates racism/inequity,* 0 = *neutral,* 1 = *incorporates anti-racism/equity*). One category, research studies, was assessed on a 2-point scale (0 = *neutral—presents research study without discussion of baseline characteristics of study population,* 1 = *incorporates anti-racism/equity regarding baseline characteristics of study population*). To pilot test the rubric, the other authors (Aisha K. James, Amanda R. Jowell, Darshali A. Vyas, Jessica A. Zeidman, Rashmi Jasrasaria, and Sherri-Ann M. Burnett-Bowie) independently evaluated two different sample noon conference lectures using the rubric and gave feedback to optimize it. Two authors (Amanda R. Jowell and Michael S. Kelly) independently evaluated the DARE coaches’ pre- and posttraining assessments, and the mean of their scores was utilized. The change in total rubric score (range: −4 to 5) was the primary outcome. Paired *t* tests were used to compare pre- and posttraining assessment scores. Only individuals who completed a pre- and posttraining assessment were included in the assessment (*n* = 16). A *p* value < .05 was considered statistically significant.

The secondary outcome was participants’ perceived preparedness to apply the training. Participants (*n* = 24) responded to two questions about perceived preparedness using a 5-point Likert scale (1 = *strongly disagree*, 5 = *strongly agree*):

1. I feel more prepared to make my own lectures more anti-racist after participating in the coaching training.
2. I feel more prepared to coach presenters after participating in the coaching training.

REDCap software was used to distribute and store the pre- and posttraining assessments. Data were analyzed using R version 4.2.1 (R Core Team). We have included a timeline for executing the full curriculum, including the program evaluation (Appendix I).

## Results

### Baseline Characteristics

Between May and December 2021, we sent emails to our core internal medicine teaching residency faculty and select residents who had previously expressed interest in DARE (71 individuals). We conducted seven DARE trainings with 34 participants in total. Twenty-nine participants completed the pretraining assessment (response rate: 85%), and 24 completed the posttraining assessment (response rate = 71%), when demographic information was also collected. Sixteen participants completed both pre- and postworkshop assessments, and an additional eight individuals completed the postworkshop assessment only. Participant demographics are detailed in Table 1. Attendees were majority cisgender female, White (non-Latinx or Hispanic), attending physicians.

**Table 1.**
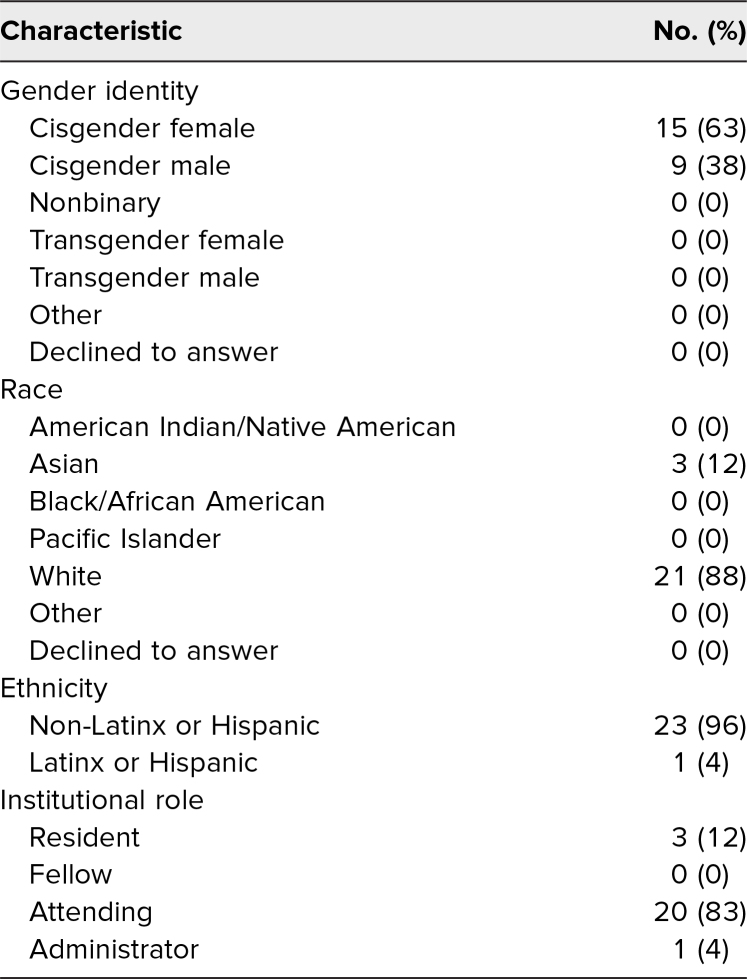
Baseline Characteristics of Participants (*N* = 24)

### Primary Outcome

The mean total rubric score for workshop attendees (*n* = 16) significantly increased after the training (from −0.5, *SD* = 1.9, to 1.9, *SD* = 1.7, *p* < .001; Table 2). Attendees demonstrated a statistically significant improvement in rubric scores related to diversity of graphics (from −0.4, *SD* = 0.7, to 0.8, *SD* = 0.5, *p* < .001), discussion of study subjects’ race/ethnicity (from 0.1, *SD* = 0.3, to 0.8, *SD* = 0.5, *p* < .001), and discussion of the impact of racism/bias on health disparities (from 0.3, *SD* = 0.8, to 0.8, *SD* = 0.4, *p* = .03; Table 2).

**Table 2.**
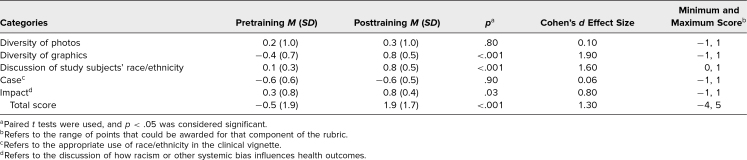
Mean Pre- and Posttraining Assessment Scores (*N* = 16)

### Secondary Outcome

After participating in the training, 23 participants (96%) indicated that they strongly agreed or agreed they felt more prepared to bring an anti-racist and equity-focused framework to their own medical curricula. Twenty-three participants (96%) indicated that they strongly agreed or agreed they felt more prepared to coach their colleagues to bring an anti-racist and equity-focused framework to their medical curricula.

## Discussion

DARE training improved participants’ ability to revise existing internal medicine residency lecture content using an anti-racist and equity-focused framework. Specifically, the training led to increased diversification of graphics, discussion of research study participants’ race and/or ethnicity (or acknowledgment of its absence), and discussion of the impact of racism on health inequities. After the training, nearly all participants endorsed feeling prepared to revise their own existing lectures and to coach colleagues in revising their existing lectures. We are encouraged by these results, as lack of preparedness has previously been identified as a particular issue among faculty.^21,24^

Our findings suggest that the DARE training can help attendees revise medical education content effectively. To our knowledge, a tool kit integrating an anti-racist and equity framework into existing residency lectures does not currently exist. Because multiple participants can be trained at once and participants are prepared to both advance anti-racism and health equity in their own work and coach others to do the same, this training can be scaled across a large teaching faculty. By training educators who give residency educational conferences, the anti-racist and equity framework can be incorporated into longitudinal curricula. One of the strengths of our training is that it is generalizable. We implemented the training with educators in internal medicine residency; however, it could be applied to all levels of medical education, from medical students to faculty members. For example, multiple medical school course directors could be trained in the same session and could then serve as coaches for the faculty in their courses, leading to powerful downstream effects across multiple curricula.

While the coaching model has powerful implications, the DARE training itself may also be beneficial for all teaching faculty, fellows, and residents from any department, regardless of whether they become coaches afterwards. DARE training could stand on its own as a program used to help prepare teaching faculty, fellows, and residents to integrate health equity frameworks into their own curricula. Furthermore, our training is accessible, takes 1 hour to complete, is conducted entirely virtually, and includes prerecorded video that can be viewed asynchronously. Given that we have provided all resources needed to execute the DARE training in this publication, we think programs will be able to use and adapt these materials for their own needs.

When adapting DARE, implementers should consider the following questions: Is there a particular part of the curriculum that would benefit from this training the most or where the training would have the most impact? Is there a part of the curriculum where this is most feasible? Are there members of the educational community likely to be internally motivated to participate on a voluntary basis, and how can recruitment be targeted to them? Alternatively, one could consider making the training required for certain groups or incorporating it into existing faculty development opportunities, such as faculty retreats. Although our implementation focused primarily on faculty, most residents and fellows deliver lectures during training and become the faculty of the future. DARE training could be adapted to be delivered to residents and fellows to use for their own talks by changing the framing from reviewing someone else's slides to reviewing one's own slides. Finally, because we implemented DARE training in an internal medicine residency, the sample lecture slides used in the workshop reflect common topics in internal medicine. When adapting the training to another specialty, the sample lecture slides can be edited to reflect topics common to that specialty.

Through training implementation, we learned the importance of hosting training sessions at different times (early morning, noon, and evening) to facilitate attendance. We had robust session attendance and engagement with the material. This was likely due to advertising the trainings to core teaching faculty who had a culture of reflection and continuous improvement of teaching skills. We also learned the value of having the residency leadership team's support. The emails of introduction and support sent by the program director early in the intervention facilitated participation among core faculty.

We learned that we may need to adopt different strategies to enhance participation. We advertised our training to 71 individuals (core internal medicine teaching faculty and select, interested residents), and 34 individuals participated. We think it may be useful to publicize the training to all faculty members in a department. Furthermore, it may be helpful to create incentives for participation as well as address barriers. Incentives could include counting DARE training toward promotion, providing financial compensation, or including DARE training in existing and mandatory faculty development. Forgiving clinical productivity lost while attending DARE training may reduce barriers to participation.

Additionally, we learned that while fellows and residents may benefit from the training, having residents and fellows coach faculty may lead to challenging power dynamics. For this reason, we focused our efforts on recruiting attendings to serve as coaches. However, we maintain that all faculty, fellows, and residents would greatly benefit from this training as they are teachers within the hospital system.

This training was conducted at a single site, and findings may not be generalizable to other academic sites or departments. Our small sample size may limit our interpretation. While 34 individuals participated in the trainings, only 16 participants completed both pre- and posttraining assessments. Thus, it is possible that the training was not as effective among those participants who did not complete both pre- and posttraining assessments. The limited assessment completion may have been due to the additional time commitment, no protected time to complete the assessments, or lack of compensation. Furthermore, we did not see improvement in the diversity of photographs category or the appropriate use of race/ethnicity in clinical vignettes. This may have been due to the small sample size, especially in the diversity of photographs category, as there was a trend toward significance. These findings may also reflect a need to pay greater attention to teaching these concepts in future trainings, such as by incorporating them repeatedly in the workshop cases.

Our evaluation was also limited by the fact that we did not directly evaluate all our stated educational objectives due to concern that a longer evaluation would be associated with lower rates of completion. While we directly evaluated only Educational Objective 4, in order to successfully apply the checklist it is likely that participants need to be able to identify characteristics of lectures that perpetuate racism (Educational Objective 2) and describe some strategies to increase anti-racist content in lectures (Educational Objective 3). We did not evaluate whether participants could define racism or anti-racism (Educational Objective 1).

The racial and ethnic diversity of our participants was limited, and thus, results may not be generalizable to a more diverse group. Our participants reflected the limited diversity of faculty, which was consistent with other academic medical centers.^29^ Diverse participation would have brought a greater breadth of experiences to participant responses and workshop discussion. However, as participation in the training was voluntary, focused recruitment of participants identifying as underrepresented in medicine (UiM) risked exacerbating the minority tax, wherein UiM faculty and residents have additional diversity-related responsibilities that often do not lead to academic promotion.^30^

Following the training, we paired DARE coaches with educators (residents and faculty) in the department's residency noon conference series and asked the coaches to employ skills from the training to help those educators revise their presentations based on the DARE checklist. To assess the efficacy of the DARE initiative, we analyzed whether this DARE coaching led to integration of an anti-racist and equity-focused framework in the noon conference series’ content. This work was recently published.^31^ Finally, we plan to develop an asynchronous, interactive, computer-based, phase two training to replace the synchronous virtual training and improve access and scalability.

## Appendices


DARE Checklist of Best Practices.pptxPreworkshop Intro Facilitator Guide.docxPreworkshop Intro Slides.pptxWorkshop Facilitator Guide.docxWorkshop Slides.pptxPretraining Assessment.pptxPosttraining Assessment.pptxDARE Rubric.docxDARE Training Timeline.pptx
*All appendices are peer reviewed as integral parts of the Original Publication.*
