# Supplementary material for: DARE Training: Teaching Educators How to Revise Internal Medicine Residency Lectures by Using an Anti-racism Framework
Source: MedEdPORTAL. 2023 Nov 7;19:11351. doi: 10.15766/mep_2374-8265.11351 (PMC10627787; doi:10.15766/mep_2374-8265.11351)
Supplement: Supplementary file 1 — DARE Checklist of Best Practices.pptxPreworkshop Intro Facilitator Guide.docxPreworkshop Intro Slides.pptxWorkshop Facilitator Guide.docxWorkshop Slides.pptxPretraining Assessment.pptxPosttraining Assessment.pptxDARE Rubric.docxDARE Training Timeline.pptx [file mep_2374-8265.11351-s001.zip › D. Workshop Facilitator Guide.docx]

**Appendix D: Facilitator Guide for the Workshop on Using the DARE Checklist to Bring an Anti-Racist and Equity Framework to Medical Education**

**Workshop Overview:** Over the course of this 30-minute workshop, the facilitator will work with learners to use the DARE best practices checklist to edit sample lecture slides (**Appendix E**). Participants should have access to a copy of the DARE Checklist (**Appendix A**) throughout the workshop to use as a reference when suggesting these edits, as the purpose of the workshop is to have them practice using the checklist as a guide. This workshop was designed to be conducted virtually but can also be conducted in-person. Prior to the workshop, learners should have watched the assigned training video (A recording of **Appendix B+C**) and should be familiar with checklist principles. After a brief introduction to the workshop, including naming the learning objectives and reviewing workshop organization, the facilitator will engage learners in editing 5 sample lecture slides. An anonymous virtual polling platform can be used to facilitate active learning. Learners could also answer the questions verbally.

**PowerPoint Guide:** The numbers below indicate the corresponding PowerPoint slide in **Appendix E**. Black, non-italicized font is the facilitator’s script. *Red and italicized font* are instructions for the facilitator for that slide.

1. Welcome everyone to the DARE Training Workshop.
2. On behalf of DARE Team Leadership, thank you so much for supporting this important initiative.
3. Building on what you learned watching the video, at the end of this workshop, we hope that you can:
   1. Apply the DARE best practices checklist to revise and teach medical curricula using an anti-racist and equity framework.

During the workshop we will practice revising content. We hope this skillset will serve you in revising and editing your own content to include an anti-racist and equity focused framework, as well as supporting others to do the same.

1. To do this, we will use the checklist to work through some sample slides together. We’ll start with some true-false and multiple-choice questions and then work our way to some more difficult examples. To get started, open the DARE Checklist, to use as a reference during the session. We will be using an anonymous virtual polling platform to allow you to anonymously answer questions.
2. On to practice slide 1. Imagine you are working with a faculty member about their heart failure talk and you are reviewing the slide from last year’s talk. Use the anonymous virtual polling platform to answer the following question:
   1. True or False: This slide includes a clinical calculator that includes race

*DARE training participants to use the anonymous virtual polling platform. Engage participants by acknowledging the answers coming in.*

1. This slide recommends the use of a risk calculator, the Get With the Guidelines Heart Failure (GWTG-HF) Risk Score, in which race can be used as an optional characteristic. When utilized, it gives a lower mortality risk prediction for people identified as being Black. However, the prevalence of heart failure and heart failure associated morbidity/mortality are higher among Black individuals. In general, the use of race-based calculators such as this one is problematic, because they
   - Can conflate race with genetics.
   - Identify race, not racism, as the risk factor for disease. Differences in outcomes are multifactorial but are fundamentally grounded in differential access to resources and other sociodemographic risk factors. By naming race as the risk factor, such calculators obscure the racism driving such differential access to resources.

When you see a race-based calculator such as this, pause and use the checklist. Be sure of the following:

- - Don’t conflate race with genetics.
  - Do consider if racism, not race, or a social determinant of health, is the risk factor for disease.

You could either recommend to the faculty member to:

- - Omit the use of this calculator or recommend that people not use race correction when they use it.
  - Select a different calculator to use.
  - Use this as an opportunity to identify racism as the risk factor here that drives disparities and include accurate information on inequities in outcomes driven by racism. We have shown here on this edited slide some changes you could suggest that reflect this approach.

1. Great, now on to practice slide 2. Imagine you are working with a faculty member about their talk on cirrhosis. Using the DARE Checklist, which of the following aspects of this slide would you address?
   1. I would add pronouns.
   2. I would revise stigmatizing language.
   3. I would diversify images.

You can select as many as you wish.

*As answers trickle into the anonymous virtual polling platform, engage learners by reading out the answers. For example, one could say: I see some folks saying they would add pronouns, diversify images, etc.*

1. In this case, we took the opportunity to
   1. Add pronouns to the case, normalizing the use of pronouns.
   2. Remove stigmatizing language of “alcohol abuse.”
   3. Diversify images: if we are trying to teach about an exam finding it's important to include less and more pigmented skin so that learners can appreciate different presentations.
2. On to practice slide 3. Imagine you are assisting a faculty member on a talk about anticoagulation management. Using the DARE Checklist, which of the following aspects of this slide would you address? Do you
   1. Keep moving to the next slide…no concerns here.
   2. Look at the study population demographics to see if there is diverse representation.

*As answers trickle into the anonymous virtual polling platform, engage learners by reading out the answers.*

1. This highlights the checklist principle: Do address inclusion/exclusion of diverse populations when describing research studies. When discussing a research study, assess for diverse representation. Here is Table 1 for the study.
2. In this case, you might add a statement to address the inclusion vs. exclusion of diverse populations when presenting research studies.
3. On to practice slide 4. Imagine you are assisting a faculty member in a talk on aortic stenosis, and you come across the following slide. Based on the DARE Checklist, what edits would you potentially make on this slide? This is a free response question. Please type into the anonymous virtual polling platform some edits you could make.

*As answers trickle into the anonymous virtual polling platform, engage learners by reading out the answers.*

1. Here are some examples of edits you might make
   1. No longer default to White race as the reference range/normal range
   2. Add information on inequities in management and outcomes.
2. Moving on to the last practice slide: Imagine you are assisting a faculty member in a talk on COVID-19. Based on the DARE Checklist, what edits would you suggest on this slide? This is a free response question. Please type into the anonymous virtual polling platform some edits you could make.

*As answers trickle into the anonymous virtual polling platform, engage learners by reading out the answers.*

1. In this case, we are already doing one checklist principle. In the table, we are providing information on inequities in disease prevalence and outcomes. However, the way that we have framed that in the list of risk factors identifies race, not racism, as the risk factor. As we have seen with the other slide examples, there is no one right way to edit the content to align with best practices. Rather, there are many ways that the content could be improved. In this case we took the opportunity to:
   1. Remove race as a risk factor. And instead, identify racism as the risk factor. For example, one could say “because Black, Latinx, and Indigenous people are overrepresented in frontline jobs and communities with significant exposure to pollution, we see differential outcomes in the prevalence, morbidity, and mortality from COVID 19.”
   2. Eliminate the vaccine backlog statement that is problematic because it fails to discuss how racism contributes to differential vaccination rates.
2. We so appreciate your attendance and engagement with the DARE initiative.
