# Supplementary material for: DARE Training: Teaching Educators How to Revise Internal Medicine Residency Lectures by Using an Anti-racism Framework
Source: MedEdPORTAL. 2023 Nov 7;19:11351. doi: 10.15766/mep_2374-8265.11351 (PMC10627787; doi:10.15766/mep_2374-8265.11351)
Supplement: Supplementary file 1 — DARE Checklist of Best Practices.pptxPreworkshop Intro Facilitator Guide.docxPreworkshop Intro Slides.pptxWorkshop Facilitator Guide.docxWorkshop Slides.pptxPretraining Assessment.pptxPosttraining Assessment.pptxDARE Rubric.docxDARE Training Timeline.pptx [file mep_2374-8265.11351-s001.zip › E. Workshop Slides.pptx]

## Slide 1
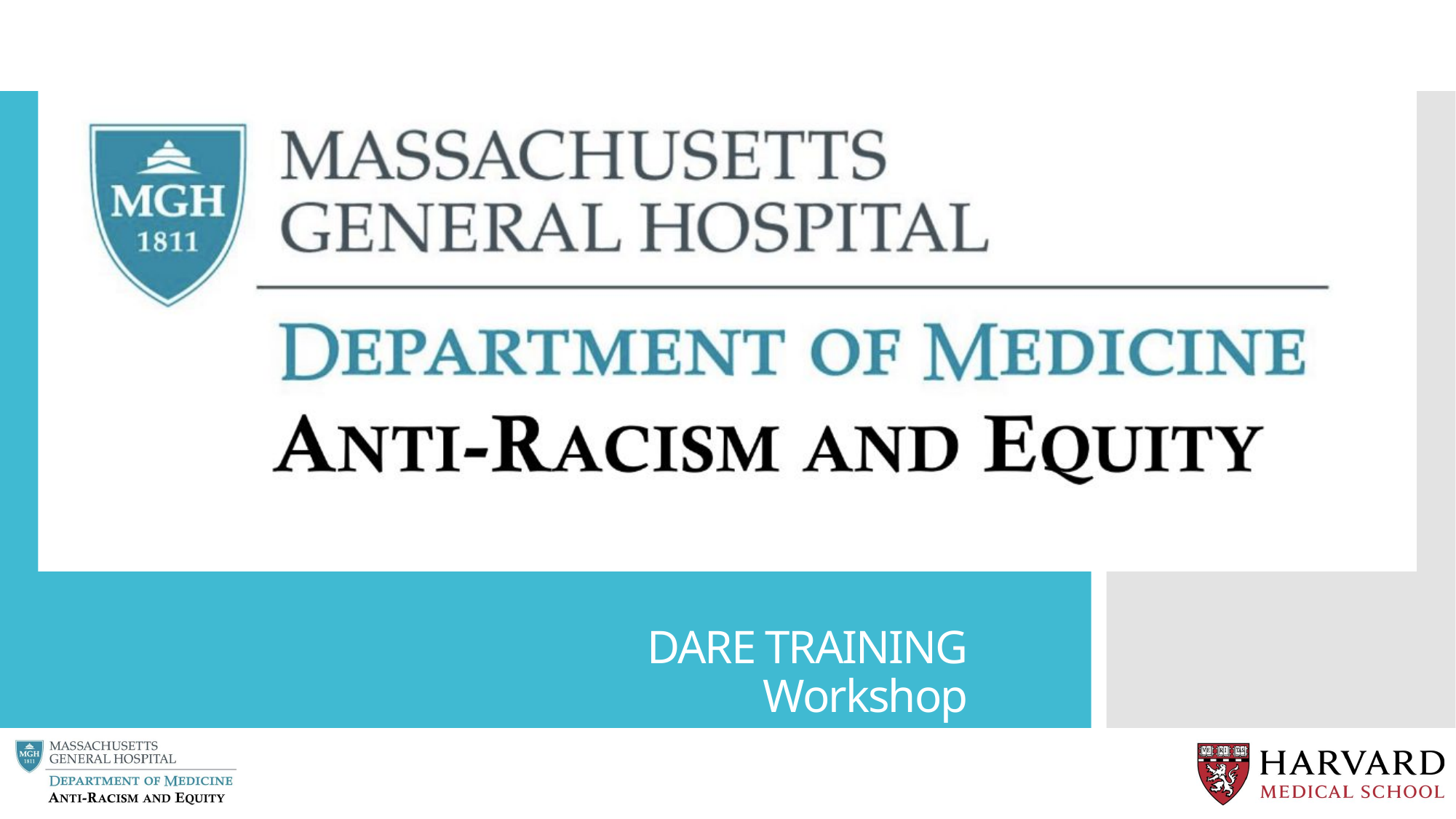

# DARE TRAINING Workshop

## Slide 2
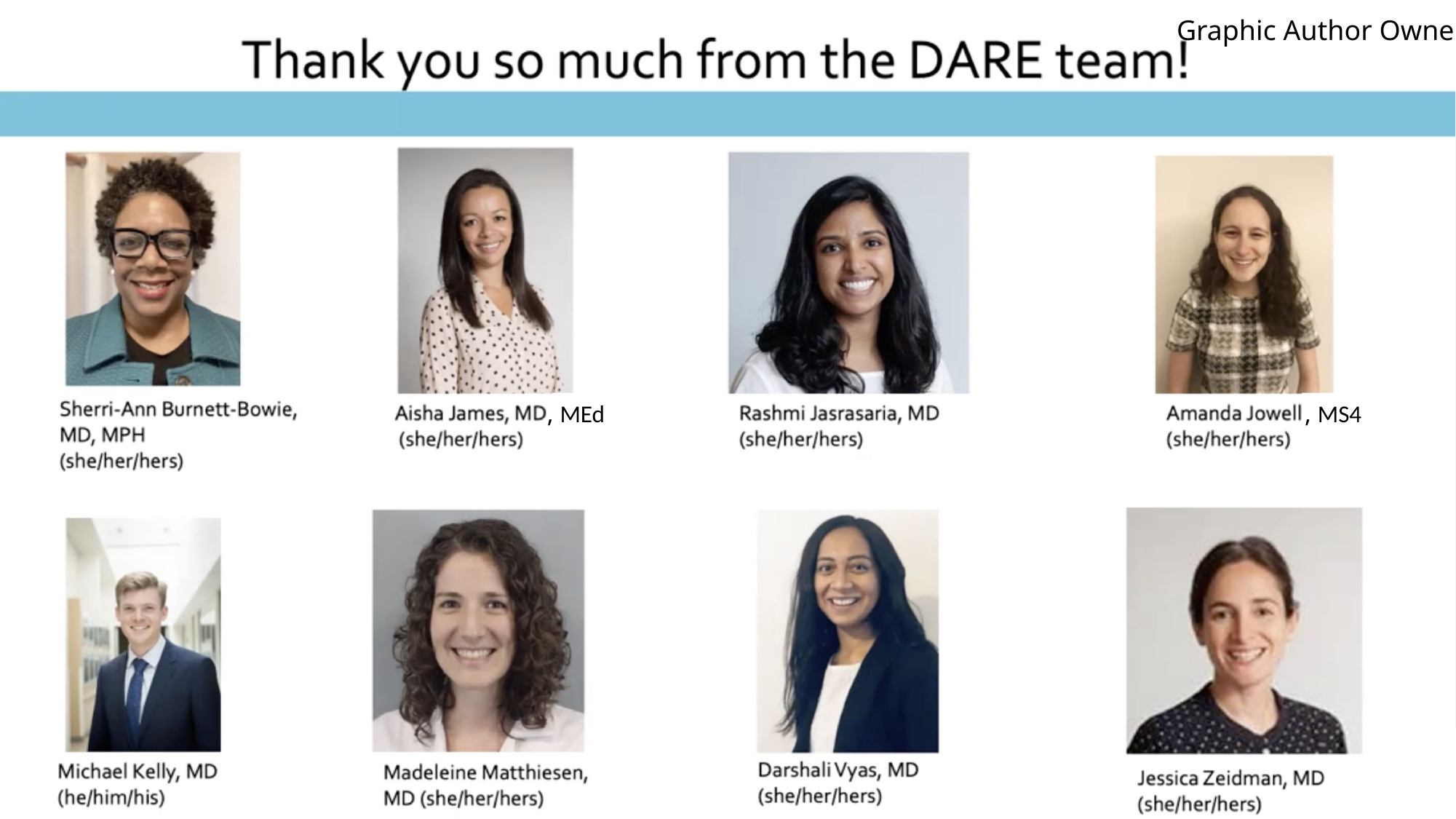

Graphic Author Owned
#
, MEd
, MS4

## Slide 3
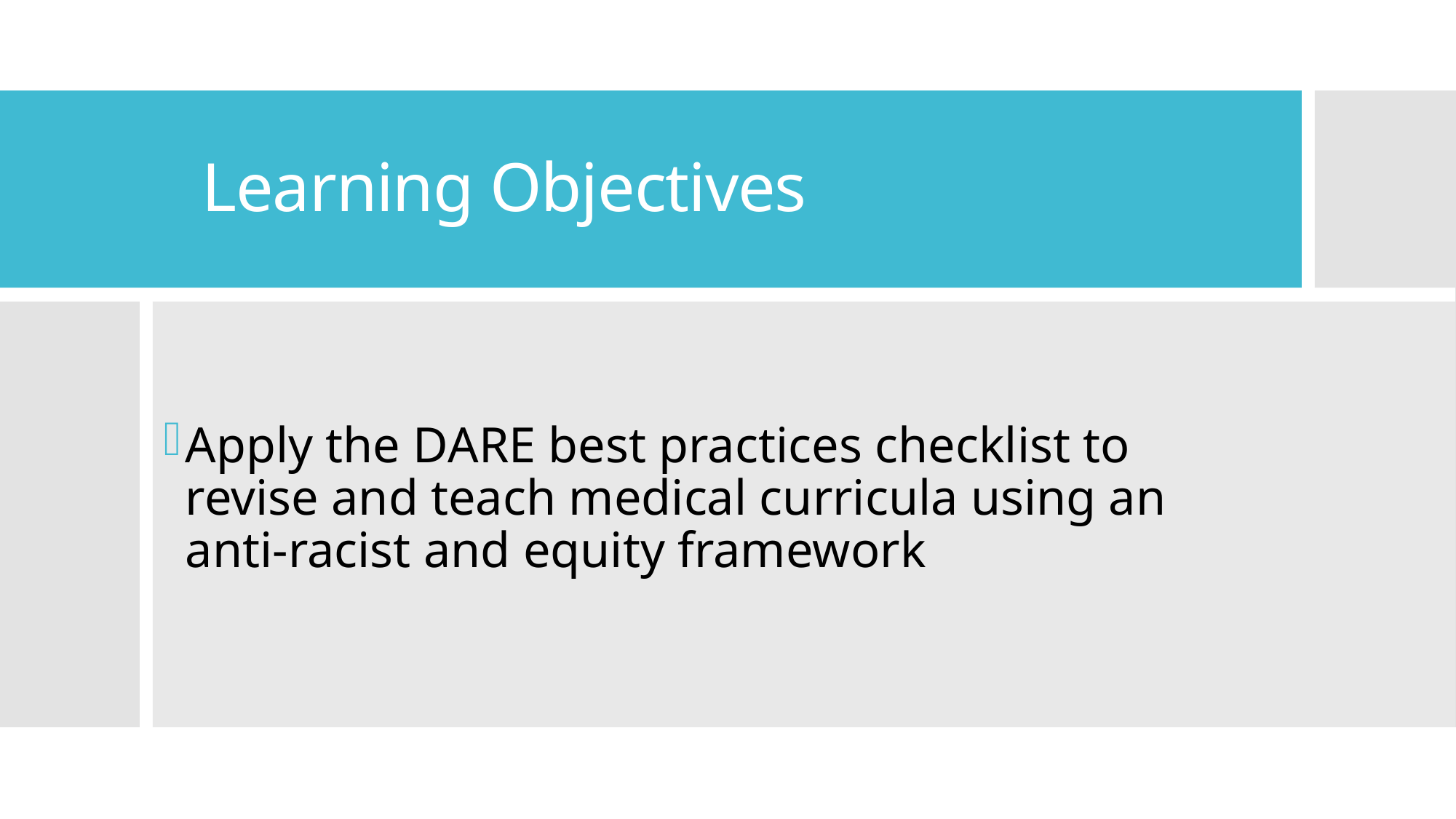

# Learning Objectives
Apply the DARE best practices checklist to revise and teach medical curricula using an anti-racist and equity framework

## Slide 4
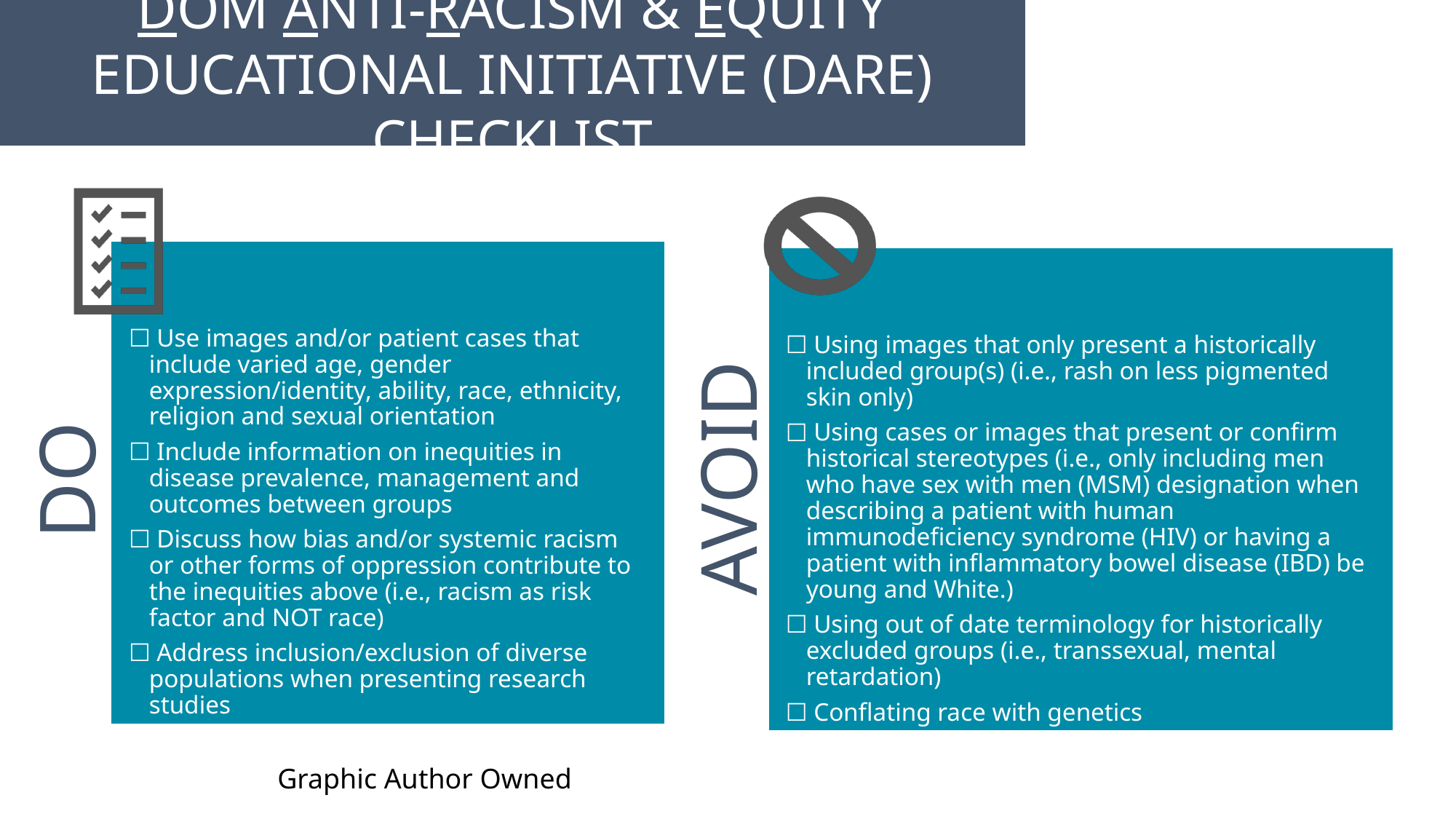

DOM Anti-Racism & Equity Educational Initiative (DARE) Checklist
Graphic Author Owned

## Slide 5
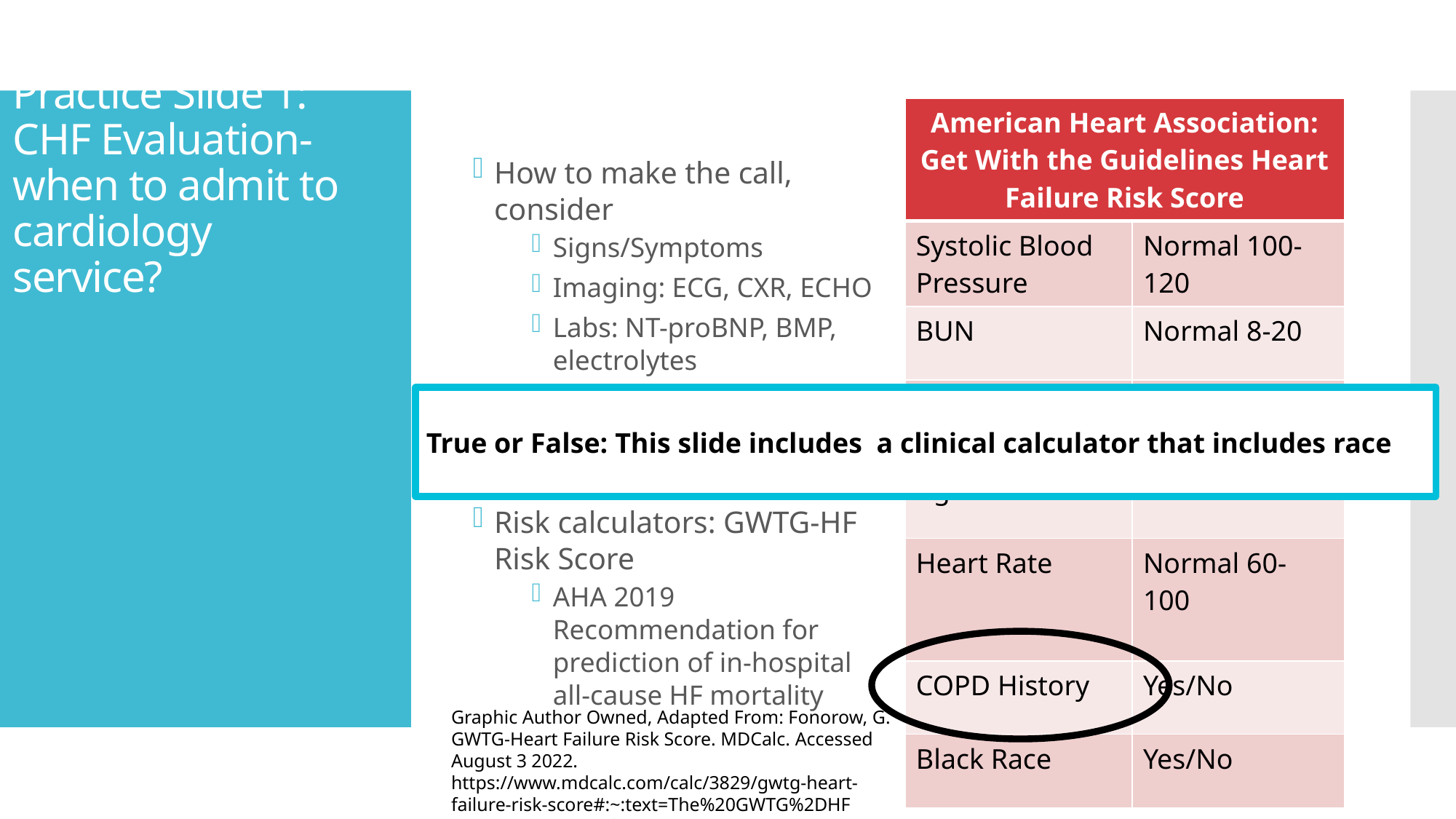

How to make the call, consider
Signs/Symptoms
Imaging: ECG, CXR, ECHO
Labs: NT-proBNP, BMP, electrolytes
Response to treatments (e.g. diuretics, beta blockers, ACE-I)
Risk calculators: GWTG-HF Risk Score
AHA 2019 Recommendation for prediction of in-hospital all-cause HF mortality
| American Heart Association: Get With the Guidelines Heart Failure Risk Score | |
| --- | --- |
| Systolic Blood Pressure | Normal 100-120 |
| BUN | Normal 8-20 |
| Sodium | Normal 135-145 |
| Age | |
| Heart Rate | Normal 60-100 |
| COPD History | Yes/No |
| Black Race | Yes/No |
Practice Slide 1: CHF Evaluation- when to admit to cardiology service?
True or False: This slide includes a clinical calculator that includes race
# Practice Slide 3: HF Evaluation- when to admit to cardiology service?
Graphic Author Owned, Adapted From: Fonorow, G. GWTG-Heart Failure Risk Score. MDCalc. Accessed August 3 2022. https://www.mdcalc.com/calc/3829/gwtg-heart-failure-risk-score#:~:text=The%20GWTG%2DHF%20Risk%20Score,for%20patients%20hospitalized%20with%20HF.

## Slide 6
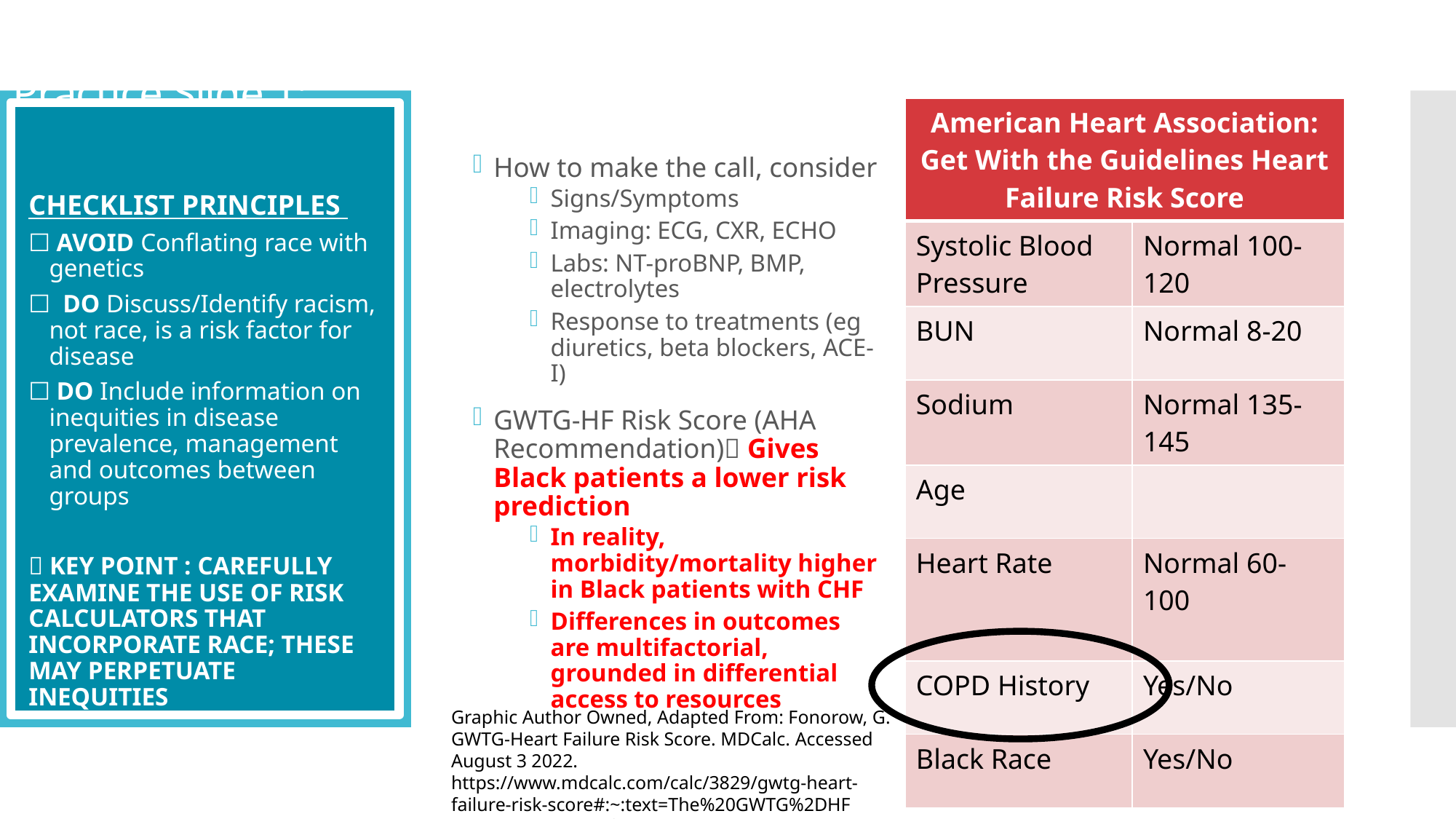

How to make the call, consider
Signs/Symptoms
Imaging: ECG, CXR, ECHO
Labs: NT-proBNP, BMP, electrolytes
Response to treatments (eg diuretics, beta blockers, ACE-I)
GWTG-HF Risk Score (AHA Recommendation) Gives Black patients a lower risk prediction
In reality, morbidity/mortality higher in Black patients with CHF
Differences in outcomes are multifactorial, grounded in differential access to resources
| American Heart Association: Get With the Guidelines Heart Failure Risk Score | |
| --- | --- |
| Systolic Blood Pressure | Normal 100-120 |
| BUN | Normal 8-20 |
| Sodium | Normal 135-145 |
| Age | |
| Heart Rate | Normal 60-100 |
| COPD History | Yes/No |
| Black Race | Yes/No |
CHECKLIST PRINCIPLES
 AVOID Conflating race with genetics
 DO Discuss/Identify racism, not race, is a risk factor for disease
 DO Include information on inequities in disease prevalence, management and outcomes between groups
 KEY POINT : CAREFULLY EXAMINE THE USE OF RISK CALCULATORS THAT INCORPORATE RACE; THESE MAY PERPETUATE INEQUITIES
Practice Slide 1: CHF Evaluation- when to admit to cardiology service?
# Practice Slide 3: HF Evaluation- when to admit to cardiology service?
Graphic Author Owned, Adapted From: Fonorow, G. GWTG-Heart Failure Risk Score. MDCalc. Accessed August 3 2022. https://www.mdcalc.com/calc/3829/gwtg-heart-failure-risk-score#:~:text=The%20GWTG%2DHF%20Risk%20Score,for%20patients%20hospitalized%20with%20HF.

## Slide 7
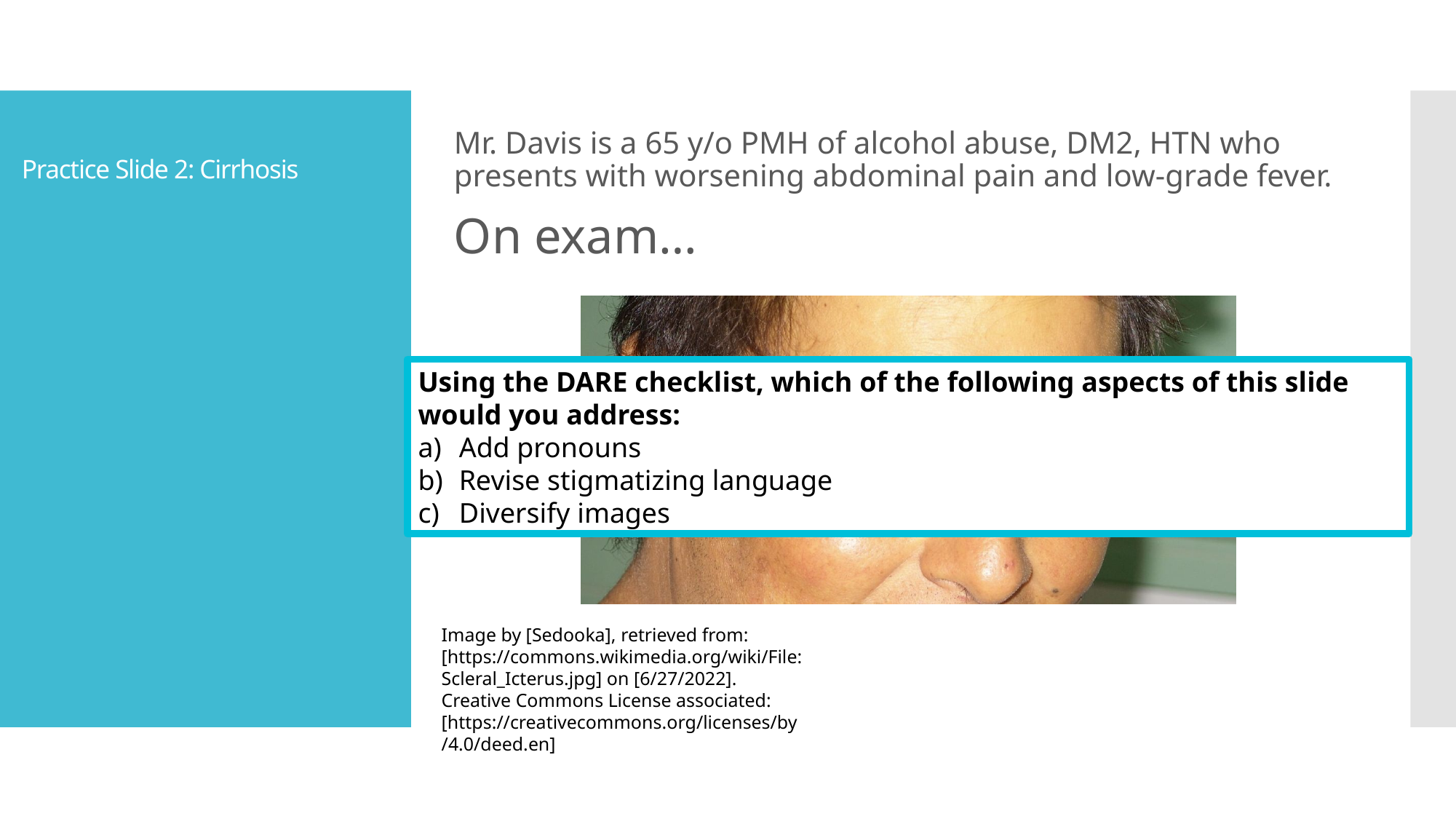

Mr. Davis is a 65 y/o PMH of alcohol abuse, DM2, HTN who presents with worsening abdominal pain and low-grade fever.
On exam…
# Practice Slide 2: Cirrhosis
Using the DARE checklist, which of the following aspects of this slide would you address:
Add pronouns
Revise stigmatizing language
Diversify images
Image by [Sedooka], retrieved from: [https://commons.wikimedia.org/wiki/File:Scleral_Icterus.jpg] on [6/27/2022]. Creative Commons License associated: [https://creativecommons.org/licenses/by/4.0/deed.en]

## Slide 8
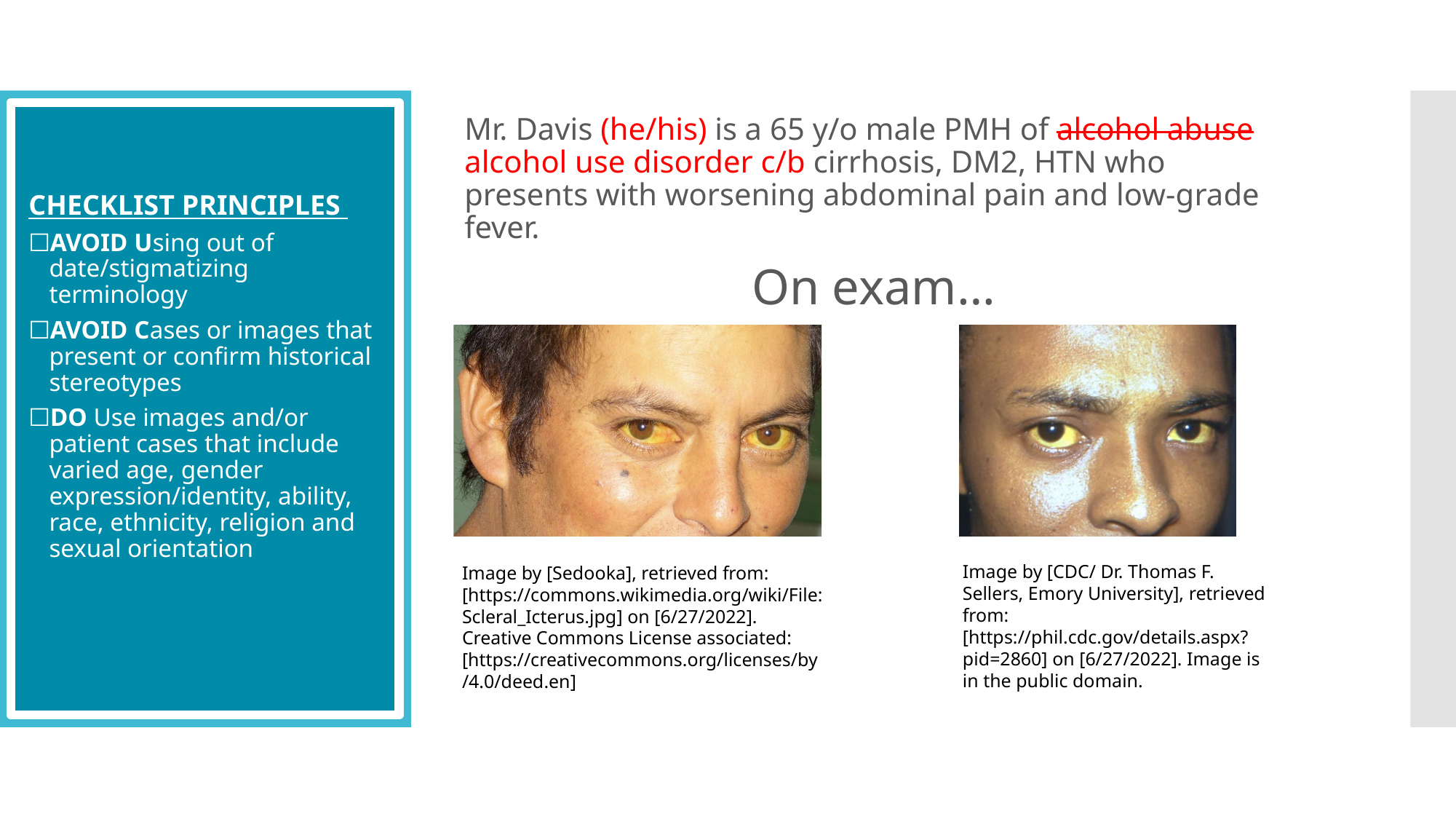

Mr. Davis (he/his) is a 65 y/o male PMH of alcohol abuse alcohol use disorder c/b cirrhosis, DM2, HTN who presents with worsening abdominal pain and low-grade fever.
On exam…
CHECKLIST PRINCIPLES
AVOID Using out of date/stigmatizing terminology
AVOID Cases or images that present or confirm historical stereotypes
DO Use images and/or patient cases that include varied age, gender expression/identity, ability, race, ethnicity, religion and sexual orientation
# Practice Slide 2: Cirrhosis
Image by [CDC/ Dr. Thomas F. Sellers, Emory University], retrieved from: [https://phil.cdc.gov/details.aspx?pid=2860] on [6/27/2022]. Image is in the public domain.
Image by [Sedooka], retrieved from: [https://commons.wikimedia.org/wiki/File:Scleral_Icterus.jpg] on [6/27/2022]. Creative Commons License associated: [https://creativecommons.org/licenses/by/4.0/deed.en]

## Slide 9
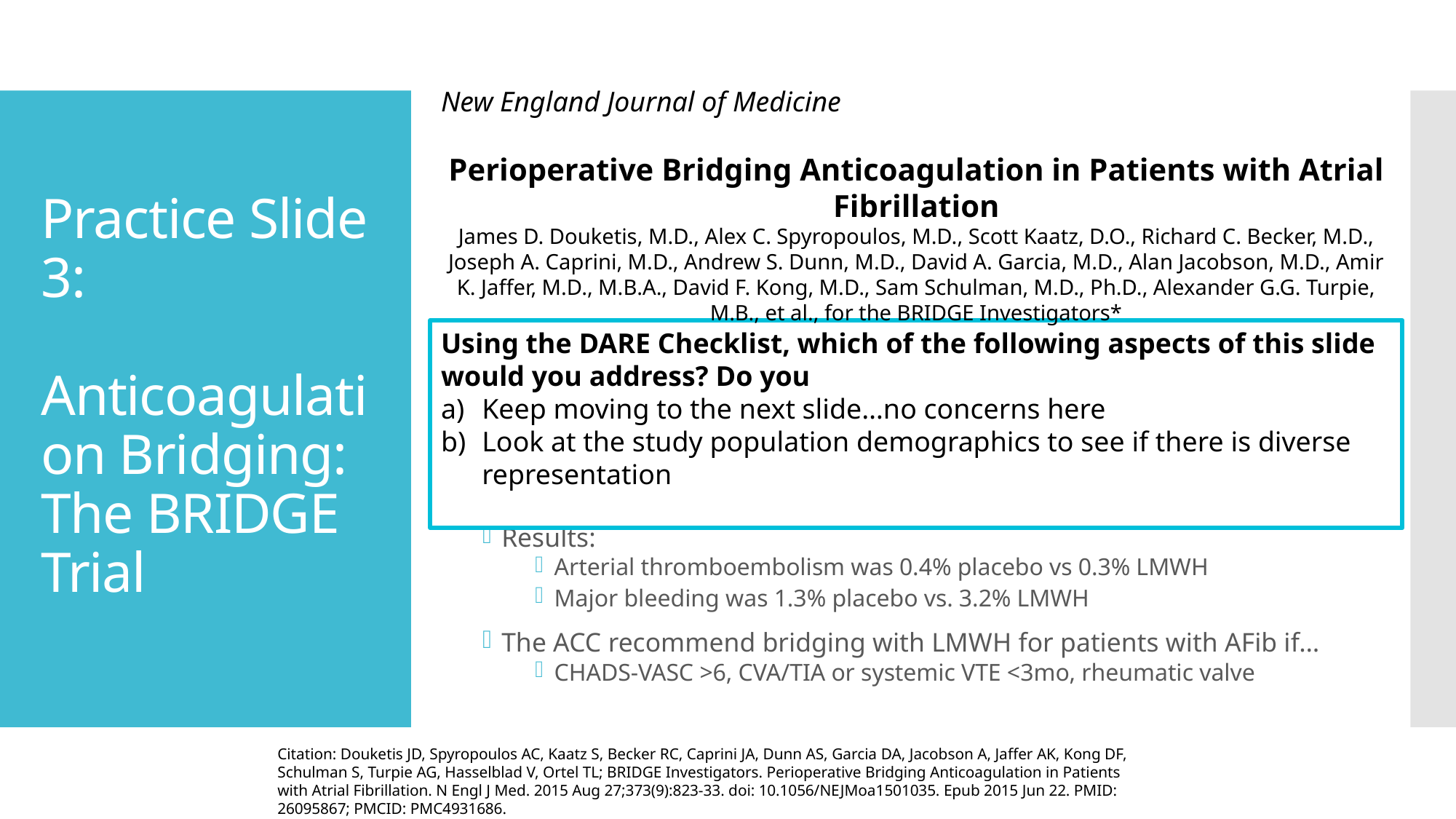

New England Journal of Medicine
Perioperative Bridging Anticoagulation in Patients with Atrial Fibrillation
James D. Douketis, M.D., Alex C. Spyropoulos, M.D., Scott Kaatz, D.O., Richard C. Becker, M.D., Joseph A. Caprini, M.D., Andrew S. Dunn, M.D., David A. Garcia, M.D., Alan Jacobson, M.D., Amir K. Jaffer, M.D., M.B.A., David F. Kong, M.D., Sam Schulman, M.D., Ph.D., Alexander G.G. Turpie, M.B., et al., for the BRIDGE Investigators*
# Practice Slide 3: Anticoagulation Bridging: The BRIDGE Trial
Using the DARE Checklist, which of the following aspects of this slide would you address? Do you
Keep moving to the next slide…no concerns here
Look at the study population demographics to see if there is diverse representation
Randomized, double-blind, placebo-controlled trial in which, after perioperative interruption of warfarin therapy, ~1800 patients were randomly assigned to receive bridging anticoagulation therapy with LMWH vs. Placebo. The primary outcomes were arterial thromboembolism (stroke, systemic embolism, or transient ischemic attack) and major bleeding.
Results:
Arterial thromboembolism was 0.4% placebo vs 0.3% LMWH
Major bleeding was 1.3% placebo vs. 3.2% LMWH
The ACC recommend bridging with LMWH for patients with AFib if…
CHADS-VASC >6, CVA/TIA or systemic VTE <3mo, rheumatic valve
Citation: Douketis JD, Spyropoulos AC, Kaatz S, Becker RC, Caprini JA, Dunn AS, Garcia DA, Jacobson A, Jaffer AK, Kong DF, Schulman S, Turpie AG, Hasselblad V, Ortel TL; BRIDGE Investigators. Perioperative Bridging Anticoagulation in Patients with Atrial Fibrillation. N Engl J Med. 2015 Aug 27;373(9):823-33. doi: 10.1056/NEJMoa1501035. Epub 2015 Jun 22. PMID: 26095867; PMCID: PMC4931686.

## Slide 10
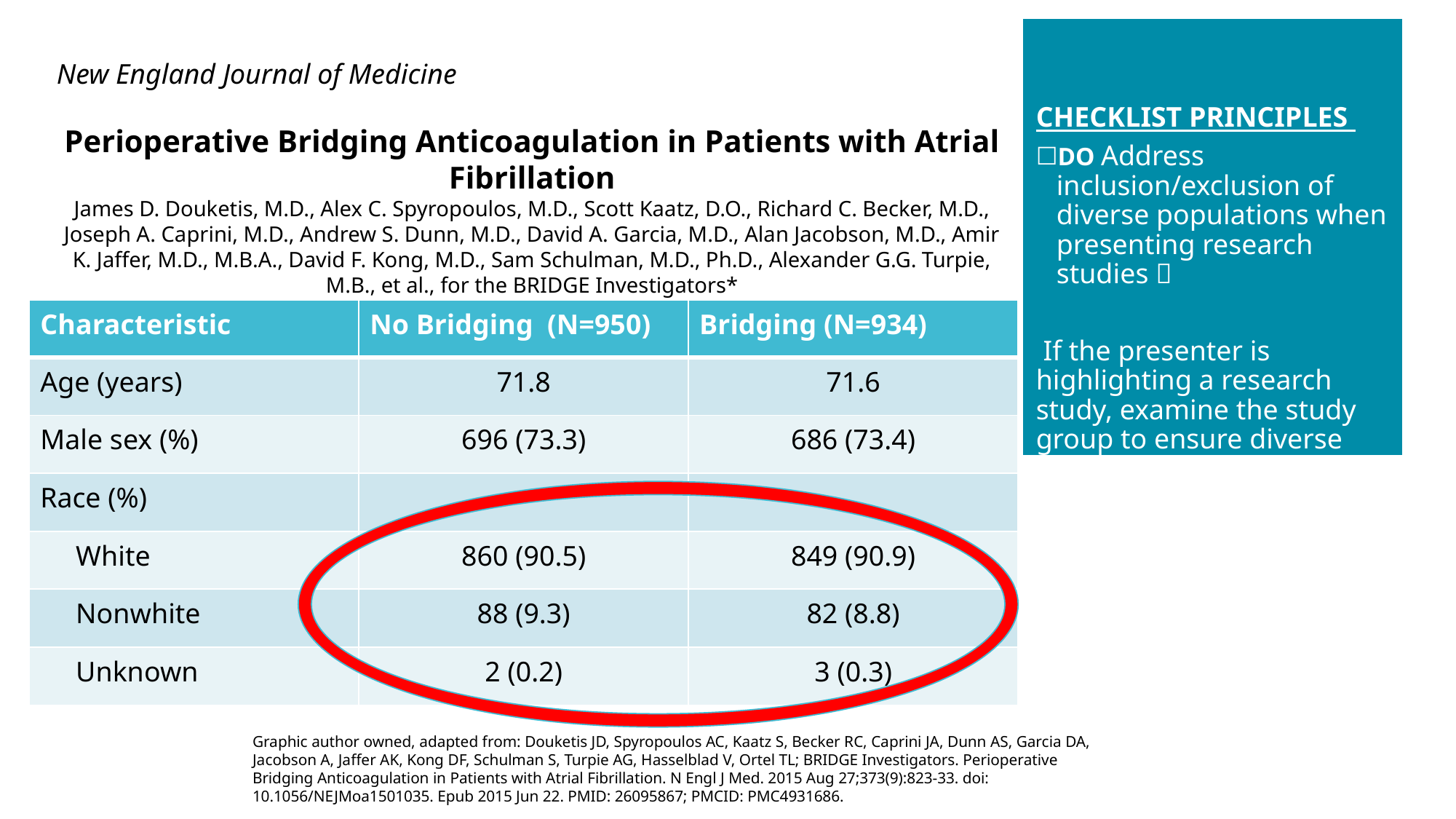

CHECKLIST PRINCIPLES
DO Address inclusion/exclusion of diverse populations when presenting research studies 
 If the presenter is highlighting a research study, examine the study group to ensure diverse representation
New England Journal of Medicine
Perioperative Bridging Anticoagulation in Patients with Atrial Fibrillation
James D. Douketis, M.D., Alex C. Spyropoulos, M.D., Scott Kaatz, D.O., Richard C. Becker, M.D., Joseph A. Caprini, M.D., Andrew S. Dunn, M.D., David A. Garcia, M.D., Alan Jacobson, M.D., Amir K. Jaffer, M.D., M.B.A., David F. Kong, M.D., Sam Schulman, M.D., Ph.D., Alexander G.G. Turpie, M.B., et al., for the BRIDGE Investigators*
| Characteristic | No Bridging (N=950) | Bridging (N=934) |
| --- | --- | --- |
| Age (years) | 71.8 | 71.6 |
| Male sex (%) | 696 (73.3) | 686 (73.4) |
| Race (%) | | |
| White | 860 (90.5) | 849 (90.9) |
| Nonwhite | 88 (9.3) | 82 (8.8) |
| Unknown | 2 (0.2) | 3 (0.3) |
Graphic author owned, adapted from: Douketis JD, Spyropoulos AC, Kaatz S, Becker RC, Caprini JA, Dunn AS, Garcia DA, Jacobson A, Jaffer AK, Kong DF, Schulman S, Turpie AG, Hasselblad V, Ortel TL; BRIDGE Investigators. Perioperative Bridging Anticoagulation in Patients with Atrial Fibrillation. N Engl J Med. 2015 Aug 27;373(9):823-33. doi: 10.1056/NEJMoa1501035. Epub 2015 Jun 22. PMID: 26095867; PMCID: PMC4931686.

## Slide 11
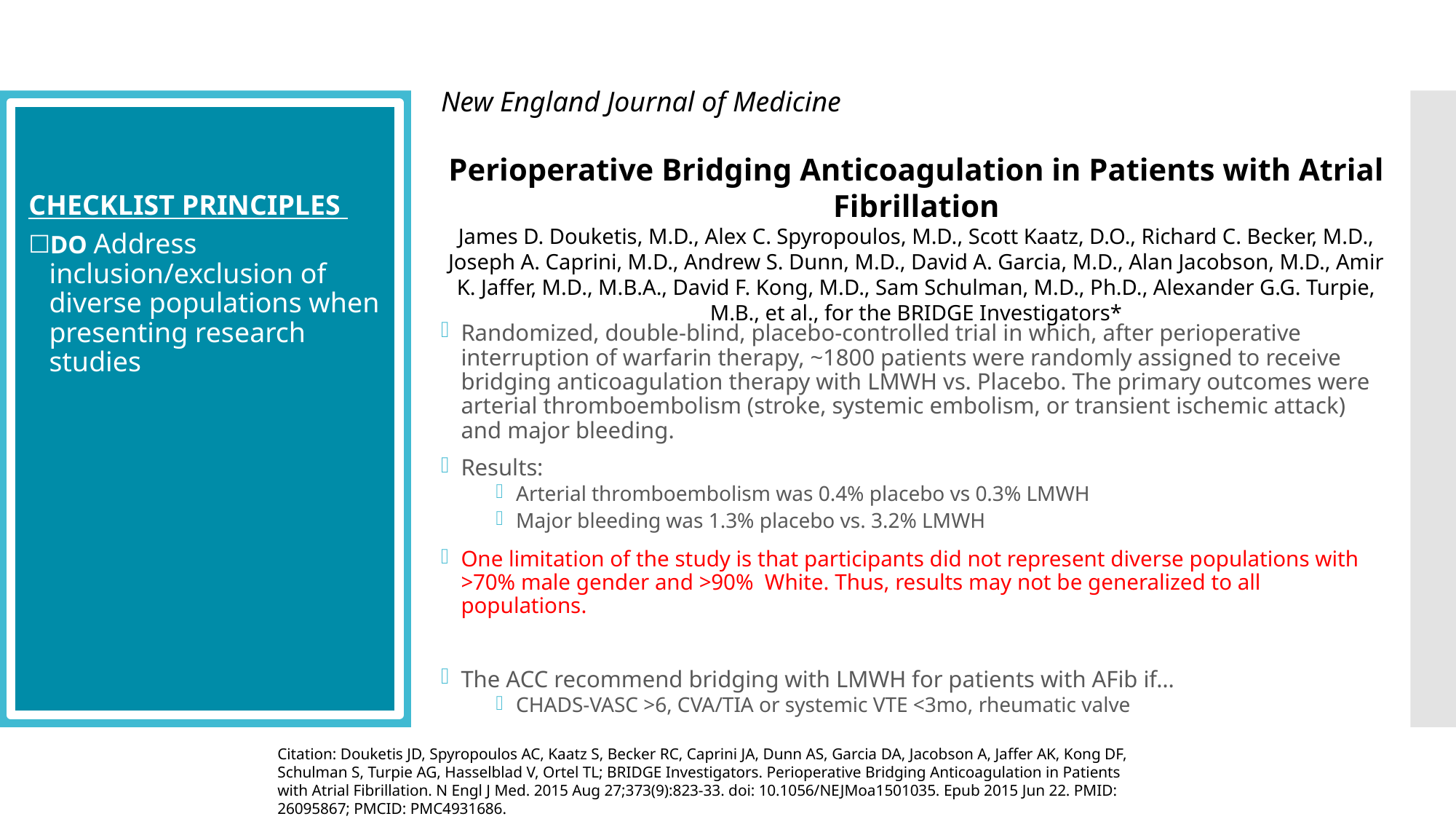

New England Journal of Medicine
Perioperative Bridging Anticoagulation in Patients with Atrial Fibrillation
James D. Douketis, M.D., Alex C. Spyropoulos, M.D., Scott Kaatz, D.O., Richard C. Becker, M.D., Joseph A. Caprini, M.D., Andrew S. Dunn, M.D., David A. Garcia, M.D., Alan Jacobson, M.D., Amir K. Jaffer, M.D., M.B.A., David F. Kong, M.D., Sam Schulman, M.D., Ph.D., Alexander G.G. Turpie, M.B., et al., for the BRIDGE Investigators*
CHECKLIST PRINCIPLES
DO Address inclusion/exclusion of diverse populations when presenting research studies
# Practice Slide 5: Anticoagulation Bridging: The BRIDGE Trial
Randomized, double-blind, placebo-controlled trial in which, after perioperative interruption of warfarin therapy, ~1800 patients were randomly assigned to receive bridging anticoagulation therapy with LMWH vs. Placebo. The primary outcomes were arterial thromboembolism (stroke, systemic embolism, or transient ischemic attack) and major bleeding.
Results:
Arterial thromboembolism was 0.4% placebo vs 0.3% LMWH
Major bleeding was 1.3% placebo vs. 3.2% LMWH
One limitation of the study is that participants did not represent diverse populations with >70% male gender and >90% White. Thus, results may not be generalized to all populations.
The ACC recommend bridging with LMWH for patients with AFib if…
CHADS-VASC >6, CVA/TIA or systemic VTE <3mo, rheumatic valve
Citation: Douketis JD, Spyropoulos AC, Kaatz S, Becker RC, Caprini JA, Dunn AS, Garcia DA, Jacobson A, Jaffer AK, Kong DF, Schulman S, Turpie AG, Hasselblad V, Ortel TL; BRIDGE Investigators. Perioperative Bridging Anticoagulation in Patients with Atrial Fibrillation. N Engl J Med. 2015 Aug 27;373(9):823-33. doi: 10.1056/NEJMoa1501035. Epub 2015 Jun 22. PMID: 26095867; PMCID: PMC4931686.

## Slide 12
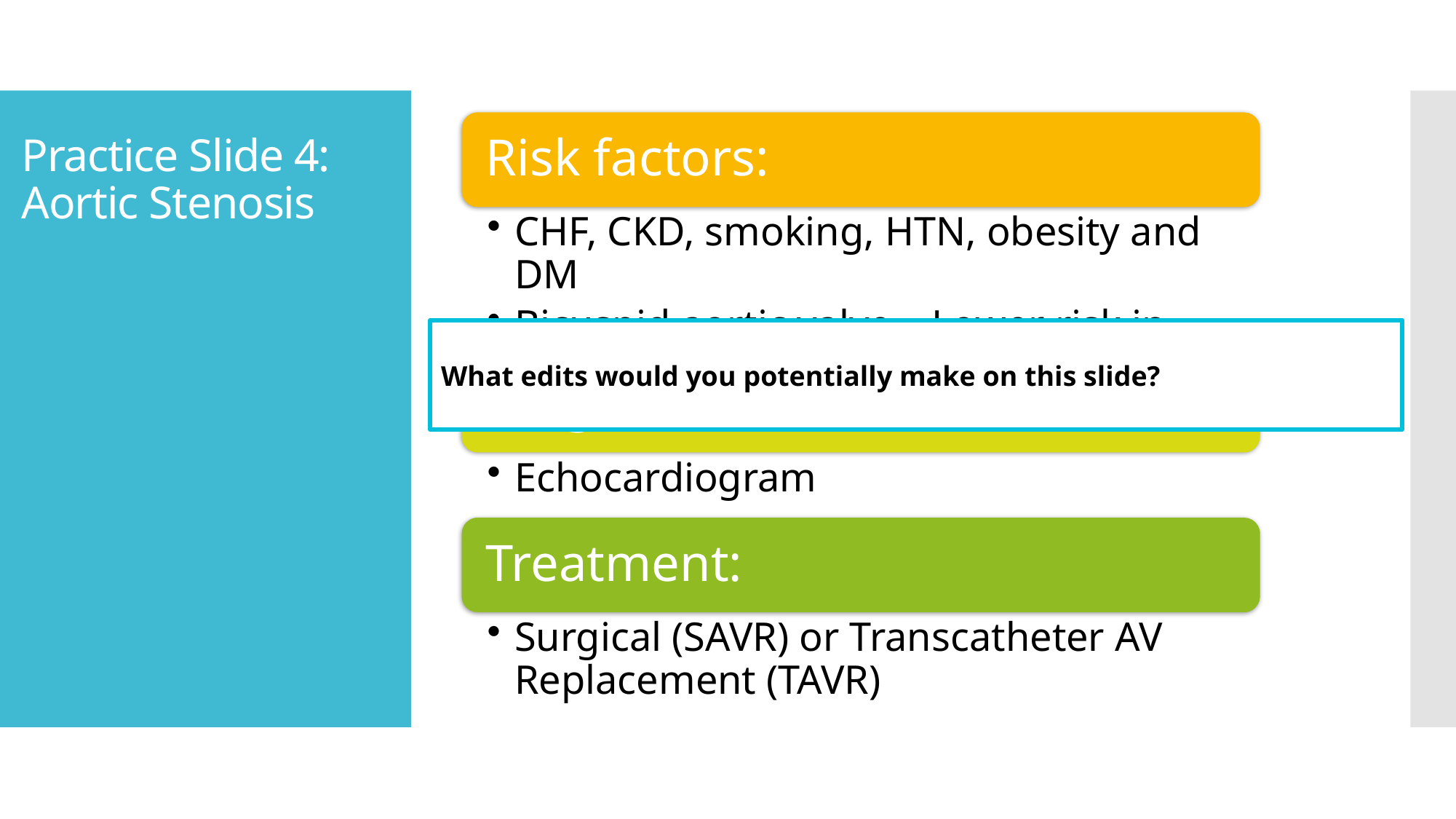

# Practice Slide 4: Aortic Stenosis
What edits would you potentially make on this slide?

## Slide 13
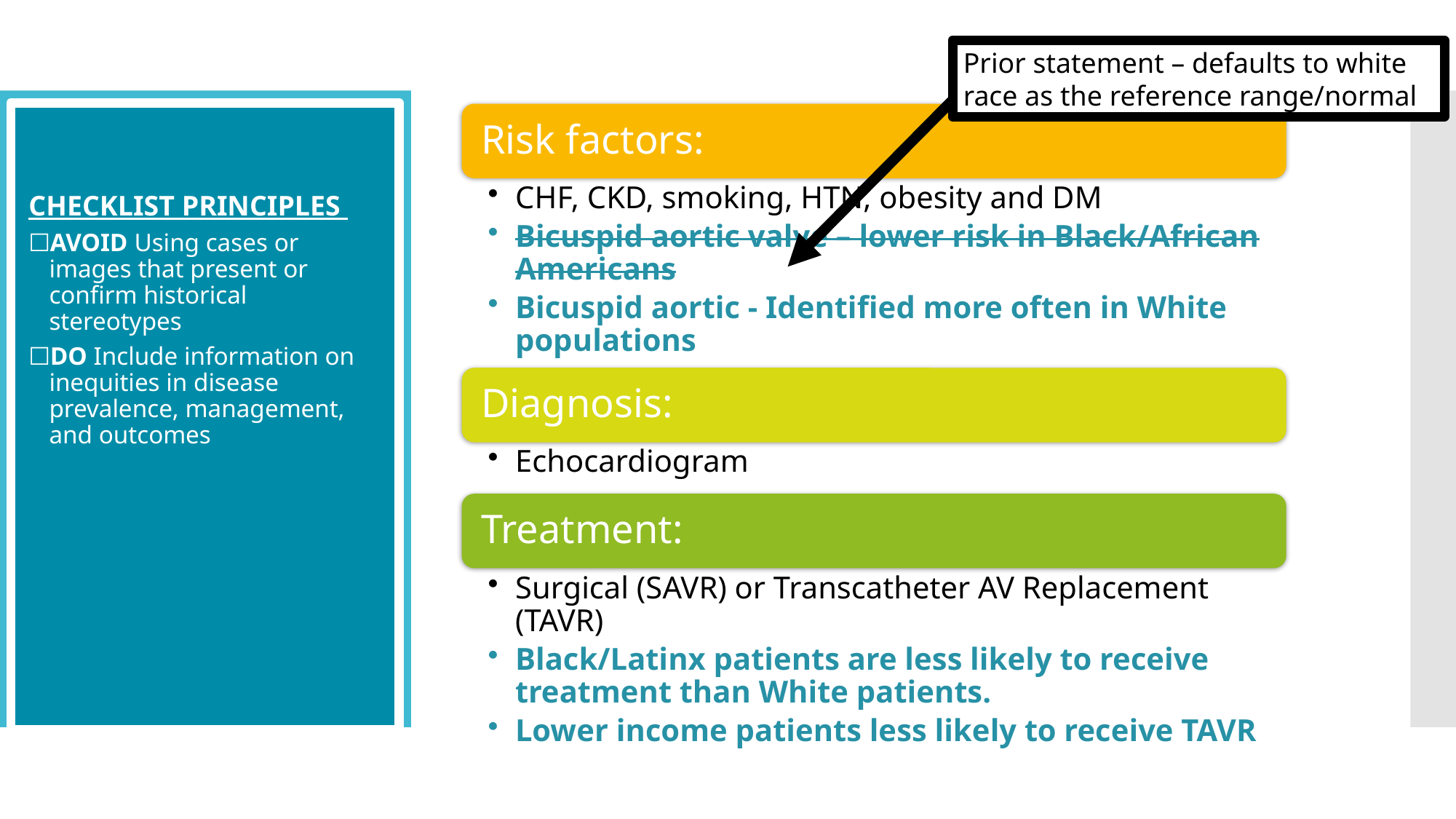

Prior statement – defaults to white race as the reference range/normal
CHECKLIST PRINCIPLES
AVOID Using cases or images that present or confirm historical stereotypes
DO Include information on inequities in disease prevalence, management, and outcomes
# Practice Slide #3: Aortic Stenosis

## Slide 14
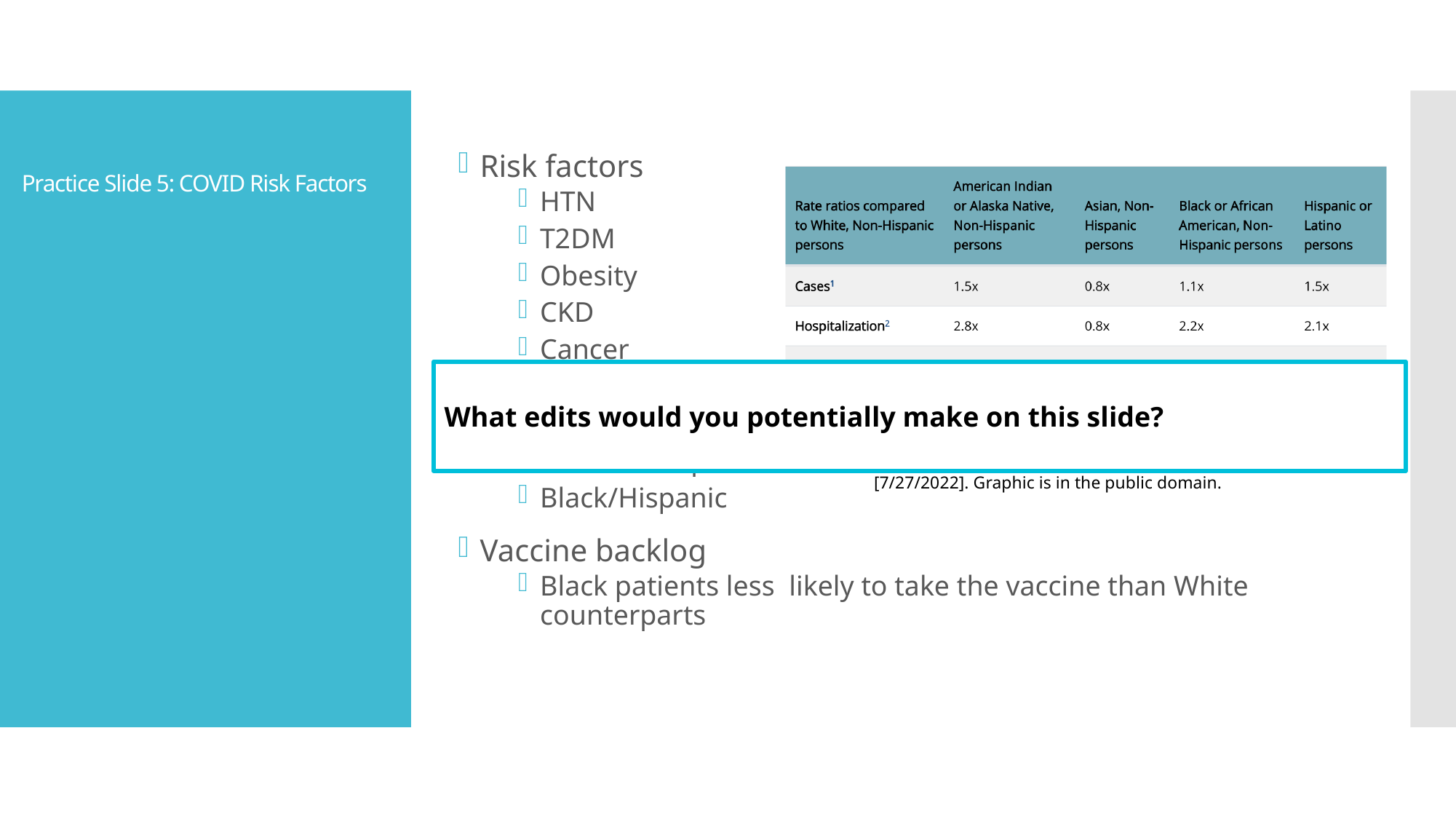

Risk factors
HTN
T2DM
Obesity
CKD
Cancer
Smoking/ETOH
Working frontline jobs
Pollution exposure
Black/Hispanic
Vaccine backlog
Black patients less likely to take the vaccine than White counterparts
# Practice Slide 5: COVID Risk Factors
What edits would you potentially make on this slide?
Graphic from [CDC.Gov], retrieved fromhttps://www.cdc.gov/coronavirus/2019-ncov/covid-data/investigations-discovery/hospitalization-death-by-race-ethnicity.html] on [7/27/2022]. Graphic is in the public domain.

## Slide 15
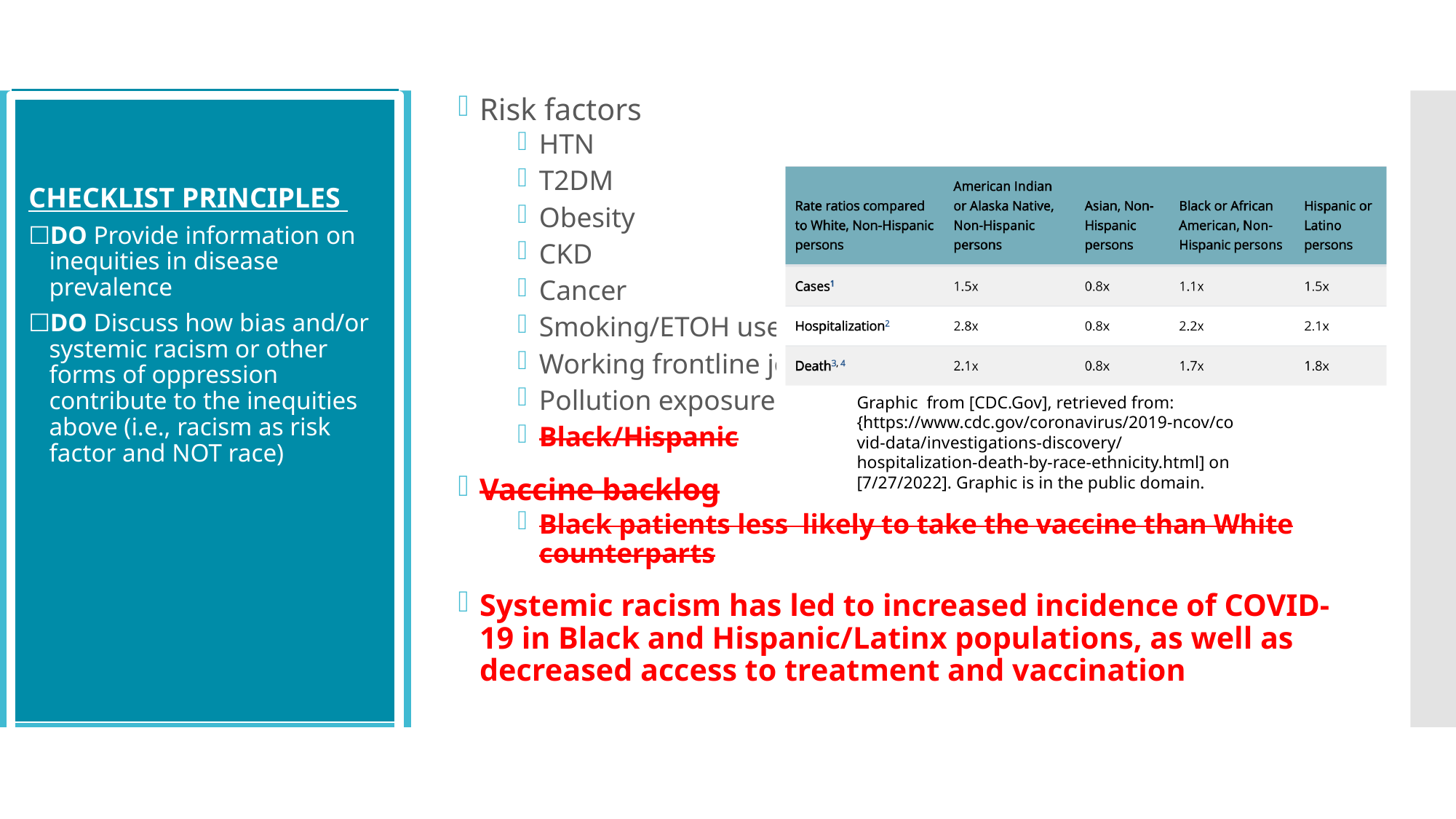

CHECKLIST PRINCIPLES
DO Provide information on inequities in disease prevalence
DO Discuss how bias and/or systemic racism or other forms of oppression contribute to the inequities above (i.e., racism as risk factor and NOT race)
Risk factors
HTN
T2DM
Obesity
CKD
Cancer
Smoking/ETOH use
Working frontline jobs
Pollution exposure
Black/Hispanic
Vaccine backlog
Black patients less likely to take the vaccine than White counterparts
Systemic racism has led to increased incidence of COVID-19 in Black and Hispanic/Latinx populations, as well as decreased access to treatment and vaccination
# Practice Slide 4: COVID Risk Factors
Graphic from [CDC.Gov], retrieved from: {https://www.cdc.gov/coronavirus/2019-ncov/covid-data/investigations-discovery/hospitalization-death-by-race-ethnicity.html] on [7/27/2022]. Graphic is in the public domain.

## Slide 16
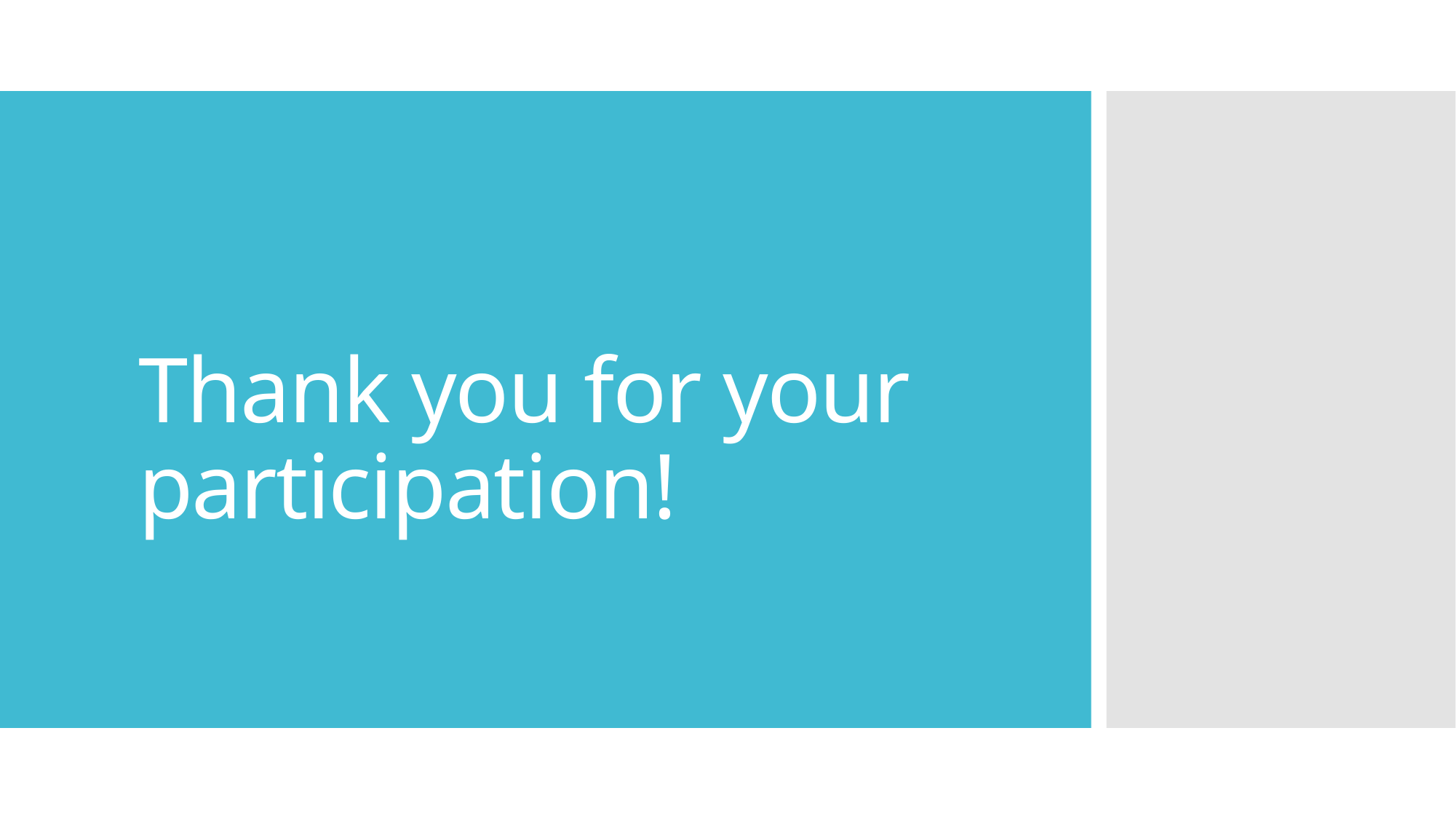

# Thank you for your participation!
