# Supplementary material for: DARE Training: Teaching Educators How to Revise Internal Medicine Residency Lectures by Using an Anti-racism Framework
Source: MedEdPORTAL. 2023 Nov 7;19:11351. doi: 10.15766/mep_2374-8265.11351 (PMC10627787; doi:10.15766/mep_2374-8265.11351)
Supplement: Supplementary file 1 — DARE Checklist of Best Practices.pptxPreworkshop Intro Facilitator Guide.docxPreworkshop Intro Slides.pptxWorkshop Facilitator Guide.docxWorkshop Slides.pptxPretraining Assessment.pptxPosttraining Assessment.pptxDARE Rubric.docxDARE Training Timeline.pptx [file mep_2374-8265.11351-s001.zip › H. DARE Rubric.docx]

**Appendix H: DARE Rubric**

*This appendix can be used to evaluate, or score, how well the DARE Checklist of Best Practices is applied to lectures slides. This rubric used to score the pre-training and post-training assessments of the coach training.*

| Skill Domain | Score |
| --- | --- |
| A) Assessment of photographs: |  |
| - Images mostly represent only historically included groups | -1 |
| - Images do not include a particular group | 0 |
| - Images mostly reflect diversity of individuals | 1 |
| - Photographs were not included | N/A* |
| B) Assessment of graphics: |  |
| - Graphics include historically excluded groups in a stereotypical way and /or fail to address the impact of bias | -1 |
| - Graphics do not contain information about historically excluded groups | 0 |
| - Graphics address impact of bias or systemic inequities on historically excluded groups | 1 |
| - Graphics were not included | N/A* |
| C) Assessment of research studies: |  |
| - Research participants’ race/ethnicity not described or discussed | 0 |
| - Research participants’ race/ethnicity described and discussed | 1 |
| - Research studies were not included | N/A* |
| D) Assessment of clinical cases: |  |
| - Historical racial/ethnic stereotypes reinforced by clinical cases | -1 |
| - Race/ethnicity of patients in clinical cases not mentioned | 0 |
| - Clinical cases move beyond historical stereotypes | 1 |
| E) Assessment of impact of race/ethnicity on disease prevalence, management, or outcomes: |  |
| - Inequities explained in ways that reinforce stereotypes/falsehoods or not discussed | -1 |
| - Impact of race on prevalence, management, or outcomes not discussed | 0 |
| - Impact of bias, systemic racism, or other forms of structural inequity on health inequities discussed | 1 |

*N/A

Total rubric score = score for A + B + C + D + E (if a component was scored as not applicable, the component was not included in the total rubric score)
