# Supplementary material for: DARE Training: Teaching Educators How to Revise Internal Medicine Residency Lectures by Using an Anti-racism Framework
Source: MedEdPORTAL. 2023 Nov 7;19:11351. doi: 10.15766/mep_2374-8265.11351 (PMC10627787; doi:10.15766/mep_2374-8265.11351)
Supplement: Supplementary file 1 — DARE Checklist of Best Practices.pptxPreworkshop Intro Facilitator Guide.docxPreworkshop Intro Slides.pptxWorkshop Facilitator Guide.docxWorkshop Slides.pptxPretraining Assessment.pptxPosttraining Assessment.pptxDARE Rubric.docxDARE Training Timeline.pptx [file mep_2374-8265.11351-s001.zip › I. DARE Training Timeline.pptx]

## Slide 1
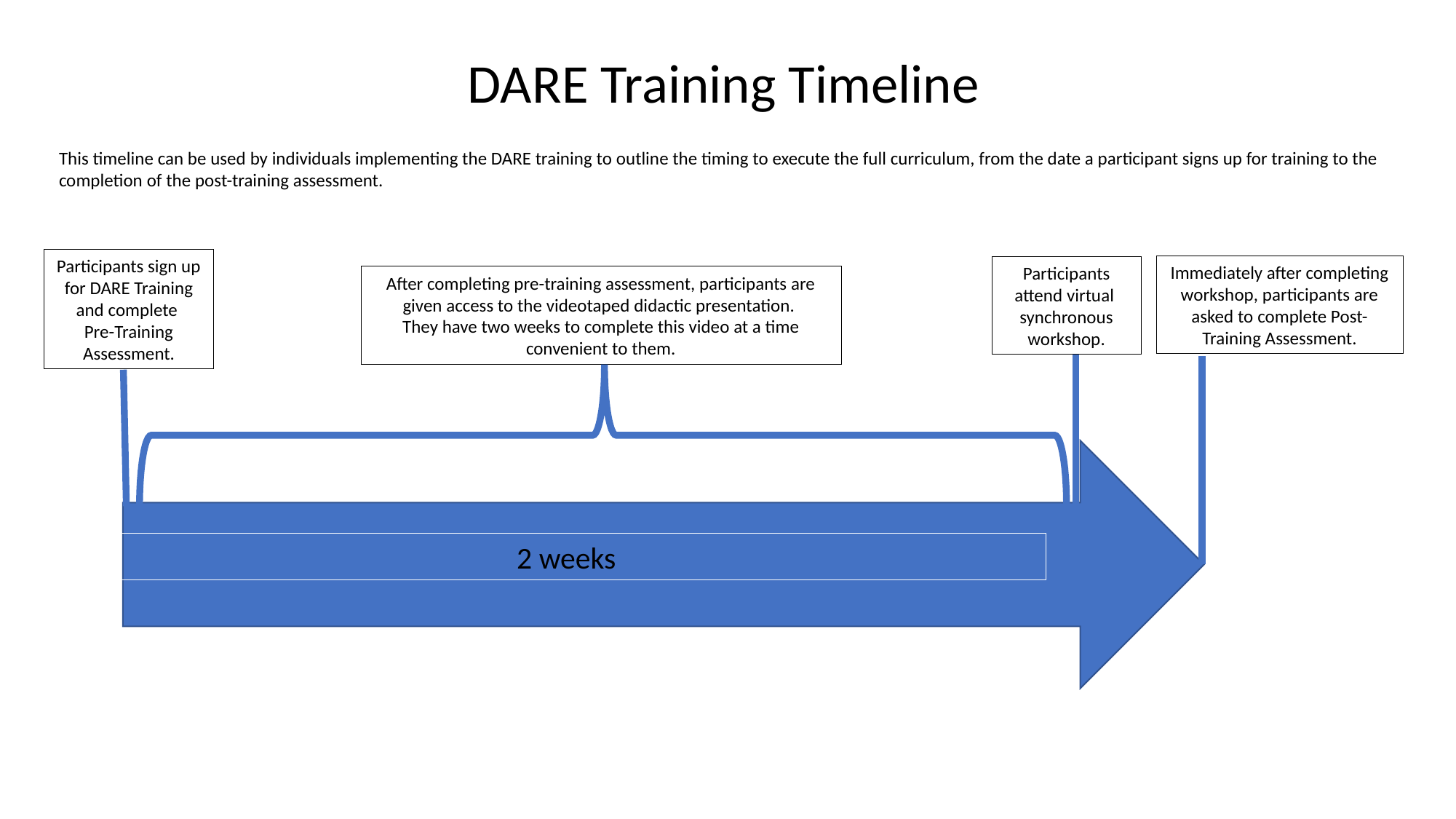

DARE Training Timeline
This timeline can be used by individuals implementing the DARE training to outline the timing to execute the full curriculum, from the date a participant signs up for training to the completion of the post-training assessment.
Participants sign up for DARE Training and complete
Pre-Training Assessment.
Immediately after completing workshop, participants are asked to complete Post-Training Assessment.
Participants attend virtual synchronous workshop.
After completing pre-training assessment, participants are given access to the videotaped didactic presentation.
They have two weeks to complete this video at a time convenient to them.
2 weeks
