# Supplementary material for: DARE Training: Teaching Educators How to Revise Internal Medicine Residency Lectures by Using an Anti-racism Framework
Source: MedEdPORTAL. 2023 Nov 7;19:11351. doi: 10.15766/mep_2374-8265.11351 (PMC10627787; doi:10.15766/mep_2374-8265.11351)
Supplement: Supplementary file 1 — DARE Checklist of Best Practices.pptxPreworkshop Intro Facilitator Guide.docxPreworkshop Intro Slides.pptxWorkshop Facilitator Guide.docxWorkshop Slides.pptxPretraining Assessment.pptxPosttraining Assessment.pptxDARE Rubric.docxDARE Training Timeline.pptx [file mep_2374-8265.11351-s001.zip › B. Preworkshop Intro Facilitator Guide.docx]

**Appendix B: Pre-Workshop Introduction Facilitator Guide**

*This script can be used to accompany the pre-workshop introduction slide deck. This presentation defines racism, anti-racism, and common examples of racism and inequities within medical practice and education. It also introduces the DARE Checklist of Best Practices to help address anti-racism and health inequities in medical education.*

*This presentation will take about 15 minutes.*

Slide 1: Welcome all, and thank you for joining us for the first part of the DARE Coach Training.

Slide 2: My name is _______ and along with the rest of my team, we are thrilled you are here, and we look forward to meeting you soon.

Slide 3: At the end of this video, we hope that you will be able to define racism and anti-racism, identify lecture characteristics that perpetuate racism and other forms of bias, and describe strategies reduce bias in lecture content and increase information about racism’s impact on health in lectures. We’ll introduce a checklist that incorporates these, along with other core principles that you will use to help support presenters in eliminating racism and other forms of bias from our conferences. We will then come together in person in the coming weeks to work through cases to apply the checklist and core principles.

Slide 4: Our DARE mission is “to ensure that educational conferences do not reinforce bias of any kind, but rather work to dismantle the structures, including racism, that obstruct our ability to follow through on the MGH mission to deliver the best health care in a safe, compassionate environment; to advance that care through innovative research and education; and to improve the health and well-being of the diverse communities we serve.”

Slide 5: In addition to this video, if you would like to learn more about identifying and eliminating racism and other forms of bias from lectures, we recommend watching Brown University Alpert Medical School’s video on creating inclusive didactics.

Slide 6: First, defining racism. We ask you to pause the video here and read through the different levels of racism.

Slide 7: Racism, not race, is the cause of the health inequities we see in the US.

Each of the four levels of racism create imbalances in power and wealth that shape policies, markets and systems. That, in turn, drives unequal distribution of the social determinants of health, such as access to safe and affordable housing, job security, quality education, healthy food, and living wages. These differences in access to social determinants of health create inequities in disease, illness and well-being.

Slide 8: To illustrate a more concrete example of this concept, let’s take a look at racism, redlining and COVID19.

Racism was the driving force behind the redlining policy of the 1930s. Per the redlining policy, the US Government systemically denied communities of color and economically disadvantaged communities access to credit and insurance, preventing them from accumulating wealth in the form of home ownership and passing on this wealth to future generations.

Limited home ownership led to power and wealth imbalances such as limited investment and tax revenue in those neighborhoods, as well as limited local control of the surrounding environment. This in turn led to widespread, inequitable distribution of social determinants of health, like:

Slide 9: Reduced property values, under-funded public schools, the departure of grocery stores, inferior access to social and health services, lack of green space and increased exposure to pollution.

Slide 10: These inequities in social determinants of health lead to elevated risk of diseases, such as hypertension, diabetes, obesity, chronic kidney disease and pulmonary disease. All of these diseases are COVID19 risk factors and help explain the increased morbidity and mortality among Black and Latinx Americans.

Slide 11: We will now turn our attention to look at some specific examples of racism in medicine. Let’s take a look at guidelines, clinical decision tools and metrics.

Slide 12: For example, the eGFR calculation as an estimate of kidney function is endorsed and recommended by multiple societies and research groups. The eGFR calculation uses race as an estimate of muscle mass, yet there is no evidence to support that race is indeed a viable proxy. This has delayed Black patients’ referral to specialists and kidney transplant wait-lists and puts Black patients at elevated risk for worsening chronic kidney disease and end stage renal disease.

Slide 13: Next let's look at implicit bias.

Slide 14: Implicit bias has been shown to influence referral for cardiac catheterization

Slide 15: Microaggressions in medicine are prevalent as well, and they affect both patients and providers.

Slide 16: An example is a patient directing all of their questions to a white resident instead of to their Black colleague.

Slide 17: Now let’s look at using race as risk factor for disease. Using race as a risk factor for disease fails to address that racism, not race, predisposes patients to disease.

Slide 18: Consider the example of hypertension and Black patients. Black Americans share the greatest burden of hypertension, and as a result, race is often erroneously listed as a risk factor for hypertension. Some use the salt-sensitivity hypothesis to explain elevated rates of hypertension in Black patients. The salt sensitivity hypothesis posits that Black Americans have a gene that allows them to retain salt more avidly than non-Black Americans.

Slide 19: There is no scientific evidence that backs this hypothesis. Instead, some of the real drivers behind elevated rates of hypertension in Black communities is structural, institutional, and interpersonal racism.

Slide 20:

Medical education also perpetuates racism.

Slide 21: As explained by LaShyra Nolen in NEJM, Medical schools and residency programs fail to teach students how to recognize disease processes in darker skin tones. They also teach what is called a “race-based diagnostic bias,” explained by Cristina Amutah et al in the NEJM. Race-based diagnostic bias is the presentation of links between racial groups and particular diseases. Let’s look at sickle cell disease. By teaching that sickle cell disease only affects Black patients rather than affecting patients who come from regions of the world at high risk for malaria, students are trained to associate sickle cell disease with Black patients. This can lead to the incorrect diagnosis of sickle cell disease in Black patients and delayed diagnosis of the sickle cell disease in non-Black patients.

Slide 22:

Lastly, institutional policies. Hospitals may only accept a limited number of low-cost insurance plans. Because of power and wealth imbalances, Black, Indigenous and communities of color are more likely to experience this restricted access to healthcare.

Slide 23:

“Anti-racism is the active process of identifying and eliminating racism by changing systems, organizational structures, policies, practices and attitudes to ensure that opportunity, power, and resources are shared equitably.”

Within the DARE context, we can strive to do this through:

Slide 24: Using images and cases with varied race, age, gender expression, sexual orientation

Slide 25: Providing cases and images that do not reinforce historical stereotypes such as IBD in a young white man

Slide 26: Identifying racism, not race, as a risk factor for disease

Slide 27: Including information on inequities in disease prevalence, management, treatment, and outcomes

Slide 28: Explaining how forms of oppression, such as implicit bias or systemic racism, drive inequities

Slide 29: Addressing both the inclusion and exclusion of certain races in research

Slide 30: Using correct terminology

This brings us to the checklist, which reflects all of these concepts!

Slide 31: This checklist will be your guide when you work with presenters. Not all points on the checklist may be applicable to a certain presentation, for example some presentations don’t use cases or include research studies, and that is absolutely fine! We encourage you to integrate as many elements of it as you are able to. Please pause here and take a moment to read it. We will be using it in our case-based workshop so we will have plenty of opportunities to practice using the checklist soon.

Slide 32: Thank you so much in advance for your work as a DARE coach! Dr. Ibram X. Kendi

captures the idea that striving to be anti-racist is a journey, not an endpoint, that we are on together. Sometimes we will make mistakes. Sometimes the presenters will make mistakes. Your role is to accompany presenters on this journey. We are not asking them to be experts, and we are not asking you to be experts. We are asking that you create a safe space to help them identify and eliminate racism and other forms of bias from their conferences. Thank you being on this journey with them and with us.

Slide 33: We now will transition to some additional guiding principles we ask you to keep in mind as you advise presenters.

Slide 34: First, we want to highlight the importance of centering and elevating BIPOC voices, experiences, activism, work and research. Consider including BIPOC’s work in both medical and non-medical spheres, such as: sociology research, news pieces, podcasts, and activism. In fact, the majority of the resources used to create this presentation are from BIPOC authors, educators, researchers and advocates.

Slide 35: An additional guiding principle is to include actionable ways for listeners to get involved and be an advocate. This means asking questions such as what is being done to combat racism in medicine, and advocate for patients and communities? What needs to be done? How can we be part of this effort?

Slide 36: Lastly, while this training is focused on identifying and eliminating racism and racial bias in lectures, we encourage you to apply these principles to address other forms of bias that you encounter as you are supporting presenters. This includes, for example, using correct pronouns in cases, addressing ageism in clinical practice, and being on the lookout for the other forms of bias you see here.

Slide 37: Thank you all for listening and we look forward to joining you in person to apply the checklist and work through some cases together at your workshop.

Slide 38: If you’d like to explore racism in medicine and medical education more deeply, here some resources we recommend. On the following slides we list all the resources that helped create this presentation.
