# Supplementary material for: DARE Training: Teaching Educators How to Revise Internal Medicine Residency Lectures by Using an Anti-racism Framework
Source: MedEdPORTAL. 2023 Nov 7;19:11351. doi: 10.15766/mep_2374-8265.11351 (PMC10627787; doi:10.15766/mep_2374-8265.11351)
Supplement: Supplementary file 1 — DARE Checklist of Best Practices.pptxPreworkshop Intro Facilitator Guide.docxPreworkshop Intro Slides.pptxWorkshop Facilitator Guide.docxWorkshop Slides.pptxPretraining Assessment.pptxPosttraining Assessment.pptxDARE Rubric.docxDARE Training Timeline.pptx [file mep_2374-8265.11351-s001.zip › C. Preworkshop Intro Slides.pptx]

## Slide 1
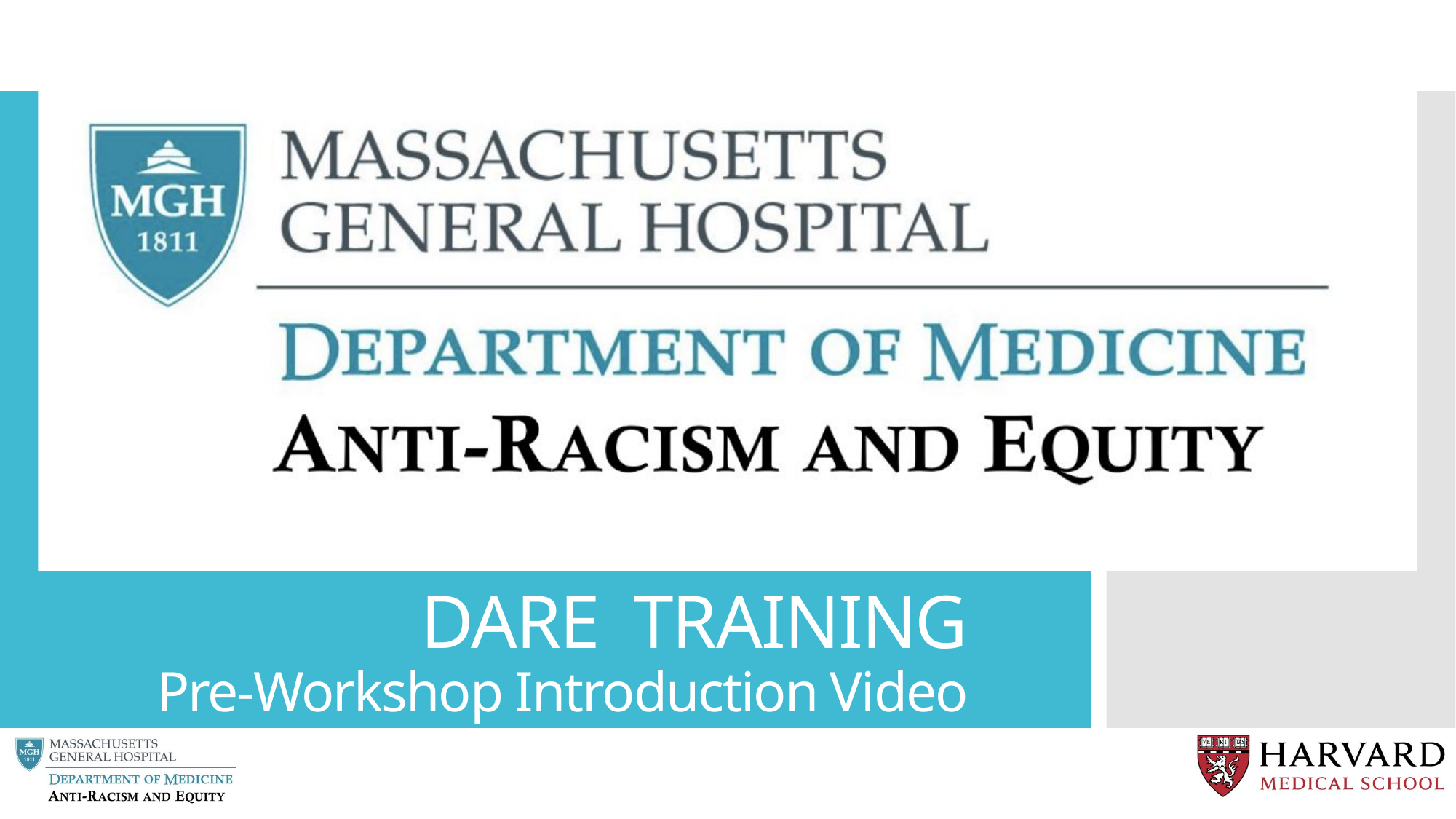

# DARE TRAINING Pre-Workshop Introduction Video

## Slide 2
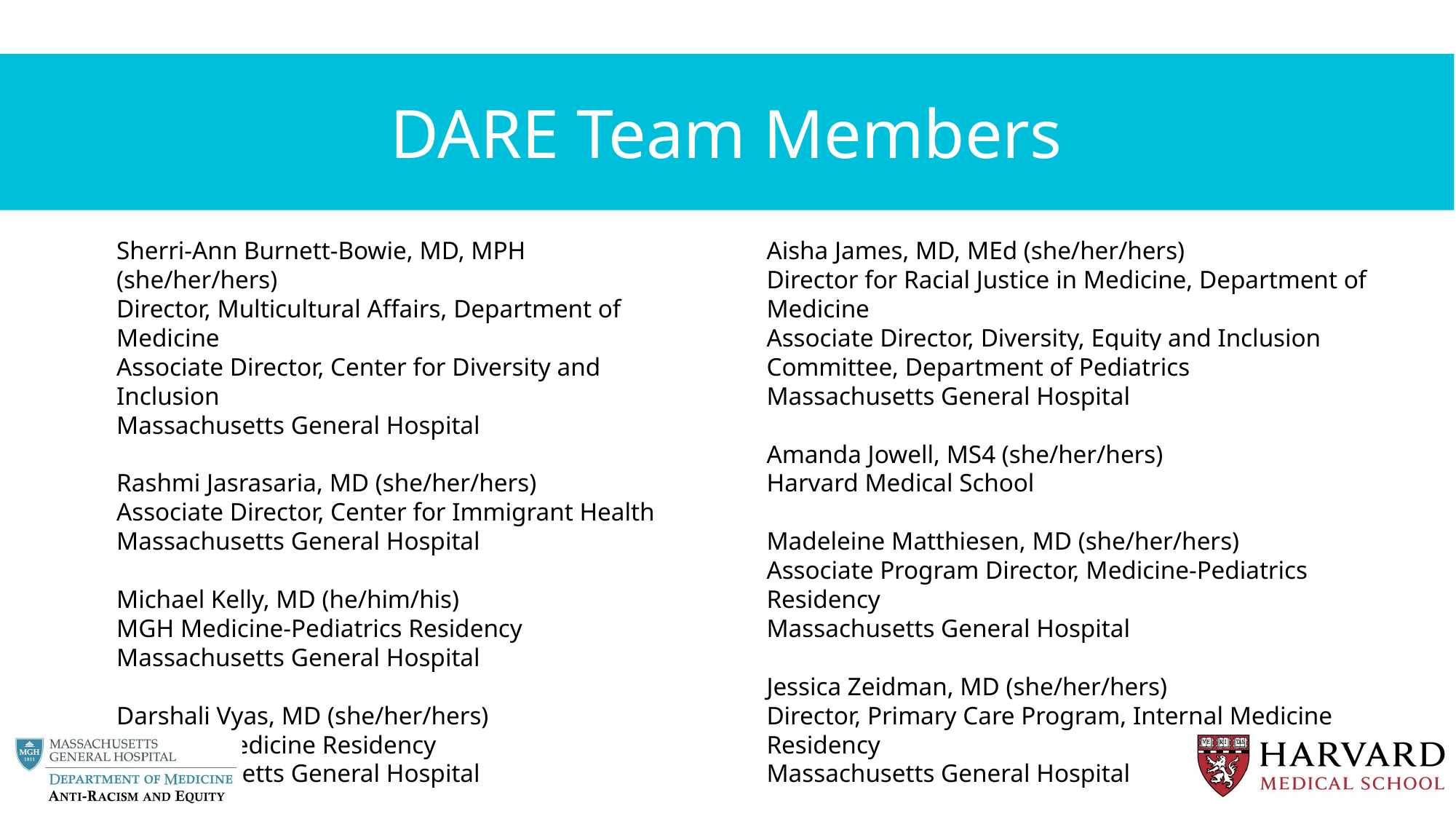

DARE Team Members
Sherri-Ann Burnett-Bowie, MD, MPH (she/her/hers)
Director, Multicultural Affairs, Department of Medicine
Associate Director, Center for Diversity and Inclusion
Massachusetts General Hospital
Rashmi Jasrasaria, MD (she/her/hers)
Associate Director, Center for Immigrant Health
Massachusetts General Hospital
Michael Kelly, MD (he/him/his)
MGH Medicine-Pediatrics Residency
Massachusetts General Hospital
Darshali Vyas, MD (she/her/hers)
Internal Medicine Residency
Massachusetts General Hospital
Aisha James, MD, MEd (she/her/hers)
Director for Racial Justice in Medicine, Department of Medicine
Associate Director, Diversity, Equity and Inclusion Committee, Department of Pediatrics
Massachusetts General Hospital
Amanda Jowell, MS4 (she/her/hers)
Harvard Medical School
Madeleine Matthiesen, MD (she/her/hers)
Associate Program Director, Medicine-Pediatrics Residency
Massachusetts General Hospital
Jessica Zeidman, MD (she/her/hers)
Director, Primary Care Program, Internal Medicine Residency
Massachusetts General Hospital

## Slide 3
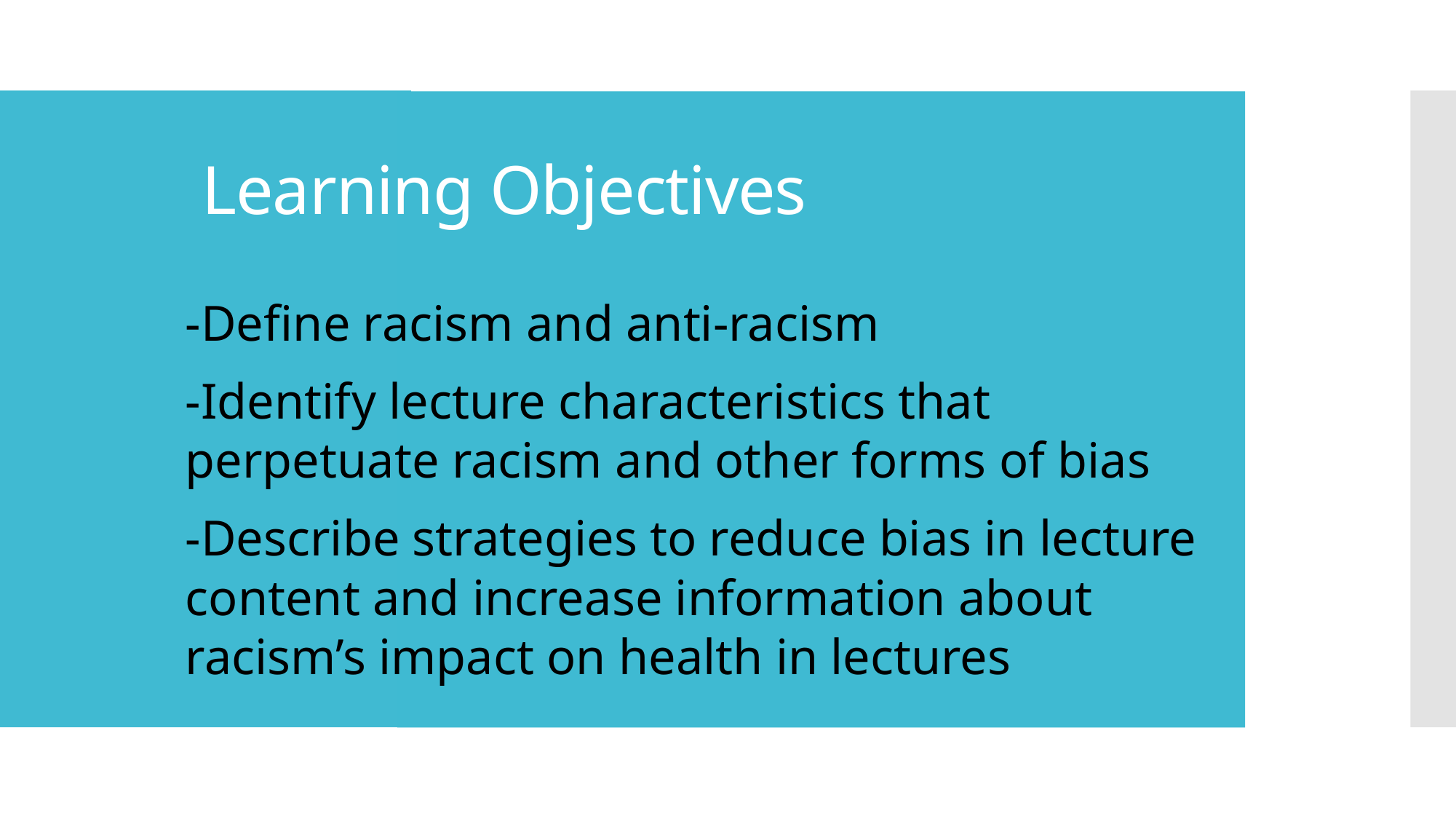

# Learning Objectives
-Define racism and anti-racism
-Identify lecture characteristics that perpetuate racism and other forms of bias
-Describe strategies to reduce bias in lecture content and increase information about racism’s impact on health in lectures

## Slide 4
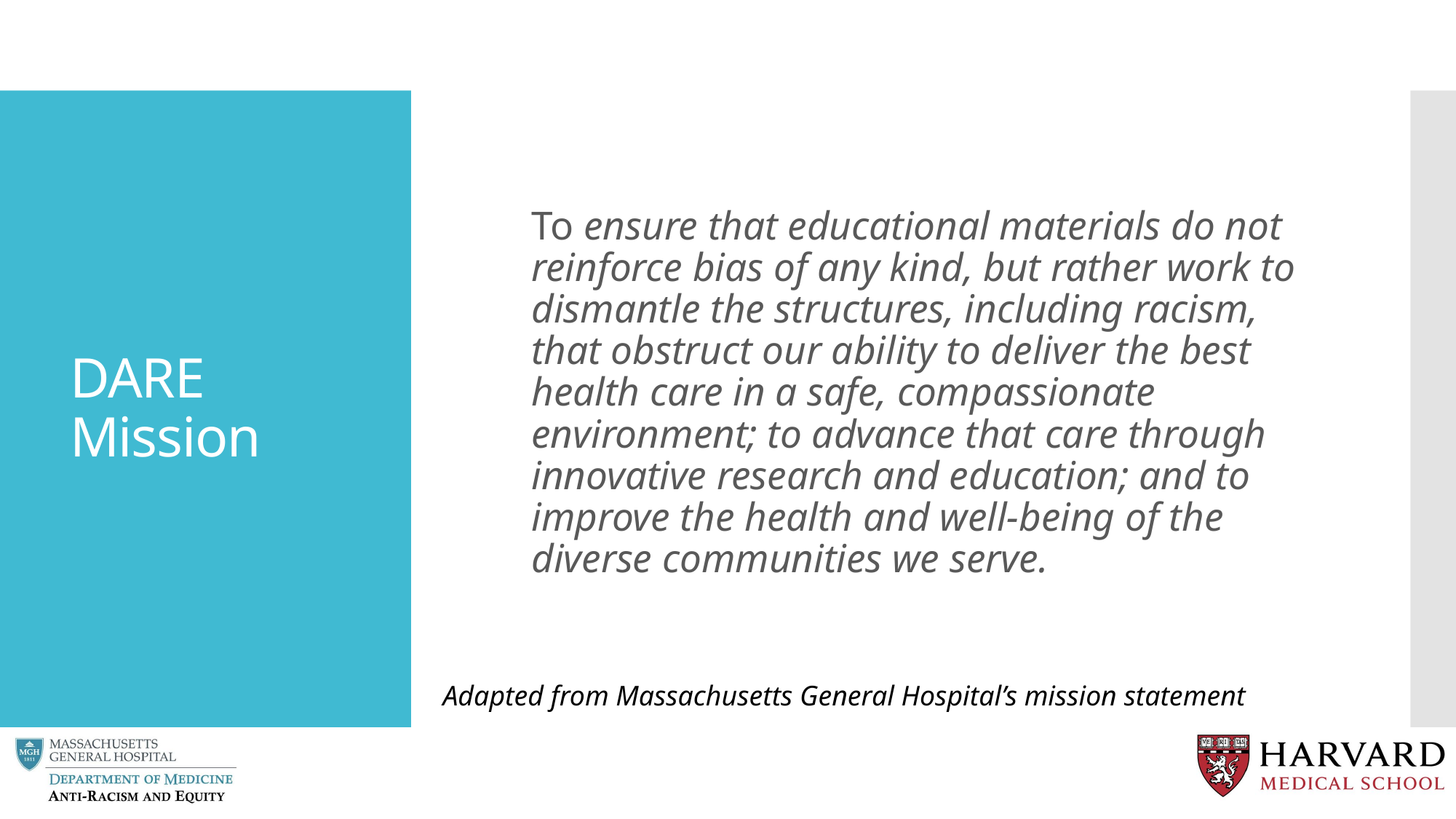

To ensure that educational materials do not reinforce bias of any kind, but rather work to dismantle the structures, including racism, that obstruct our ability to deliver the best health care in a safe, compassionate environment; to advance that care through innovative research and education; and to improve the health and well-being of the diverse communities we serve.
# DARE Mission
Adapted from Massachusetts General Hospital’s mission statement

## Slide 5
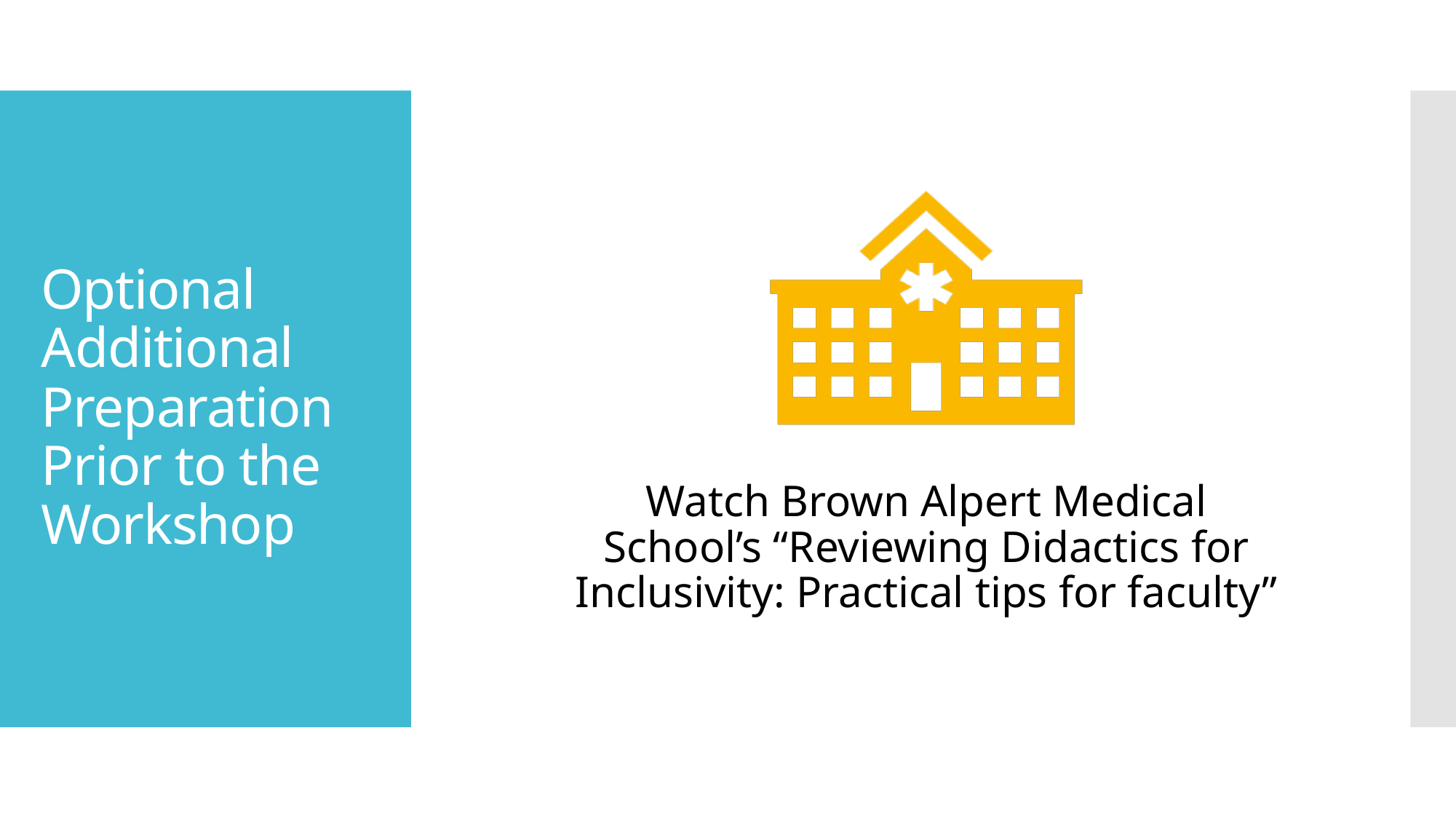

# Optional Additional Preparation Prior to the Workshop
Watch Brown Alpert Medical School’s “Reviewing Didactics for Inclusivity: Practical tips for faculty”

## Slide 6
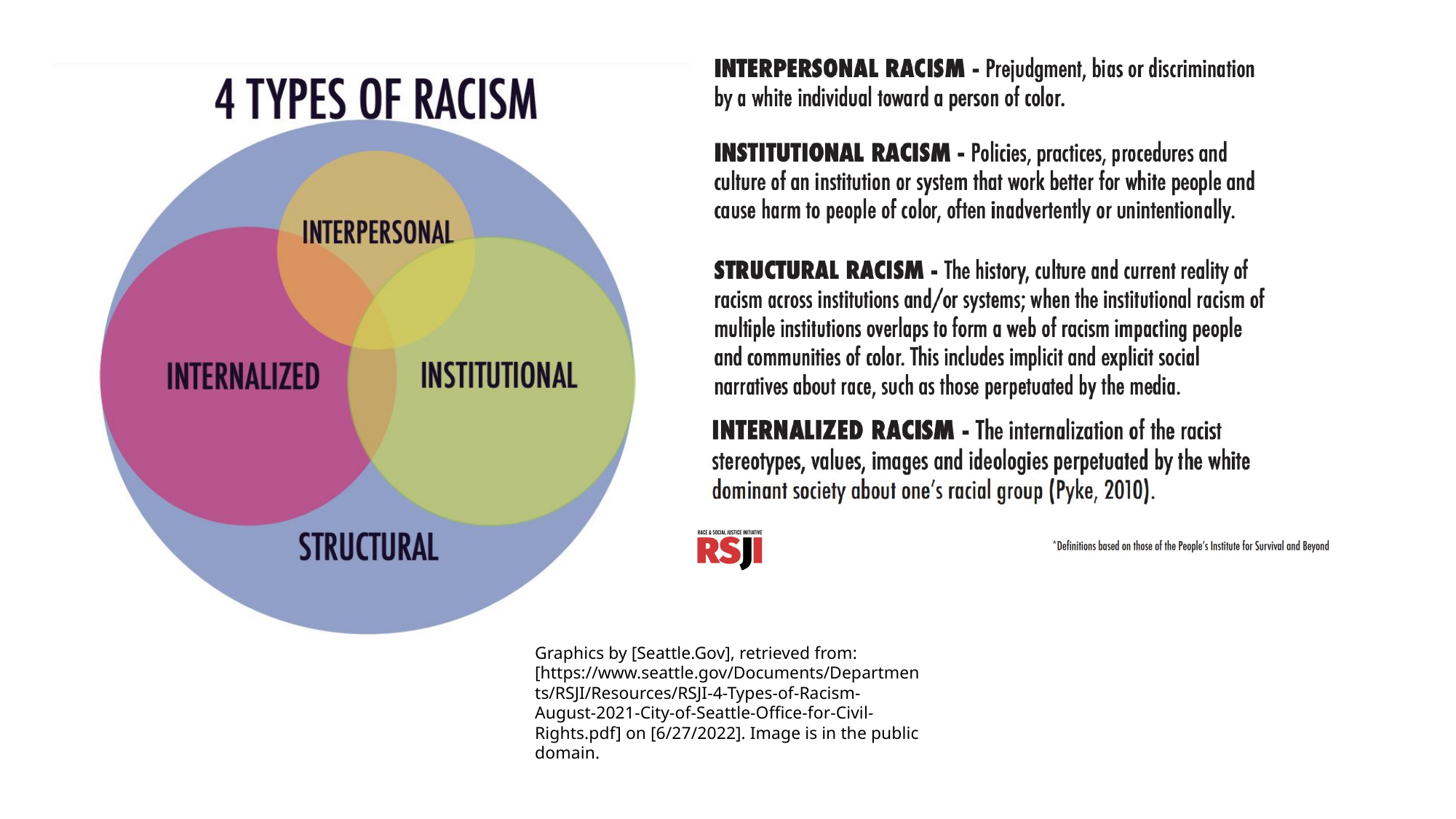

Graphic adapted from
Defining Racism
Graphics by [Seattle.Gov], retrieved from:[https://www.seattle.gov/Documents/Departments/RSJI/Resources/RSJI-4-Types-of-Racism-August-2021-City-of-Seattle-Office-for-Civil-Rights.pdf] on [6/27/2022]. Image is in the public domain.

## Slide 7
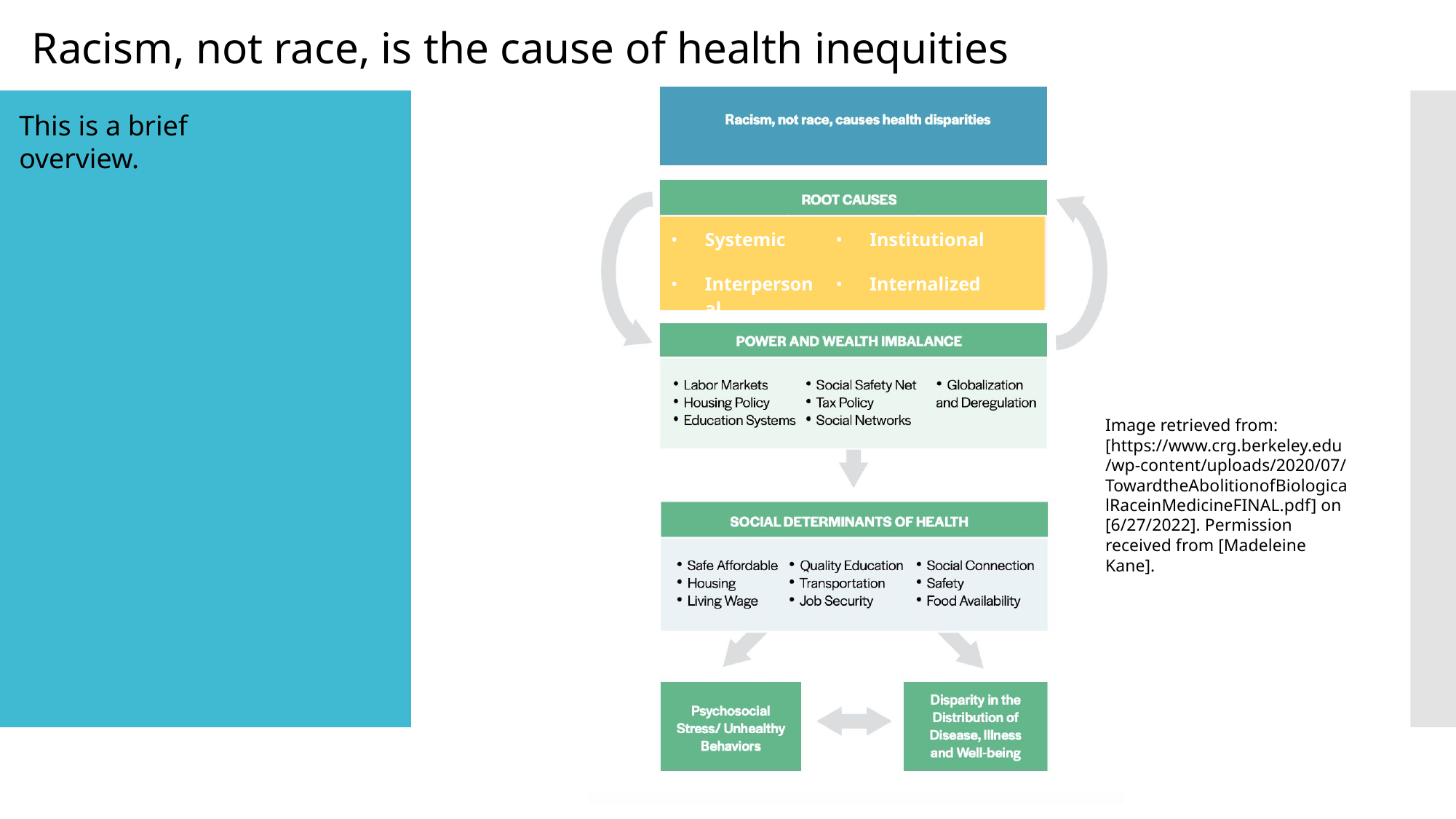

Racism, not race, is the cause of health inequities
This is a brief overview.
| Systemic | Institutional |
| --- | --- |
| Interpersonal | Internalized |
Image retrieved from: [https://www.crg.berkeley.edu/wp-content/uploads/2020/07/TowardtheAbolitionofBiologicalRaceinMedicineFINAL.pdf] on [6/27/2022]. Permission received from [Madeleine Kane].

## Slide 8
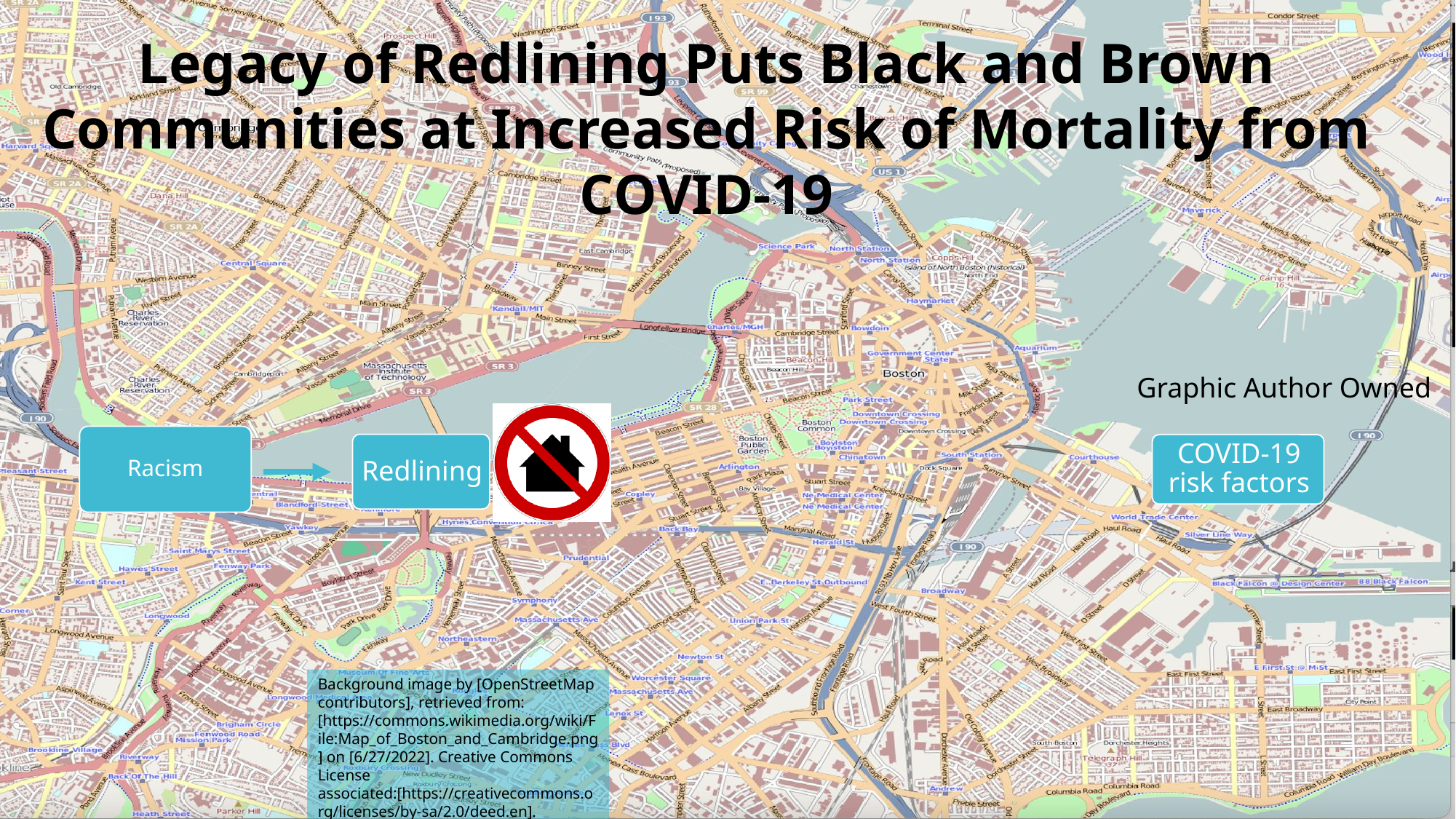

Legacy of Redlining Puts Black and Brown Communities at Increased Risk of Mortality from COVID-19
Graphic Author Owned
Racism
Background image by [OpenStreetMap contributors], retrieved from: [https://commons.wikimedia.org/wiki/File:Map_of_Boston_and_Cambridge.png] on [6/27/2022]. Creative Commons License associated:[https://creativecommons.org/licenses/by-sa/2.0/deed.en].

## Slide 9
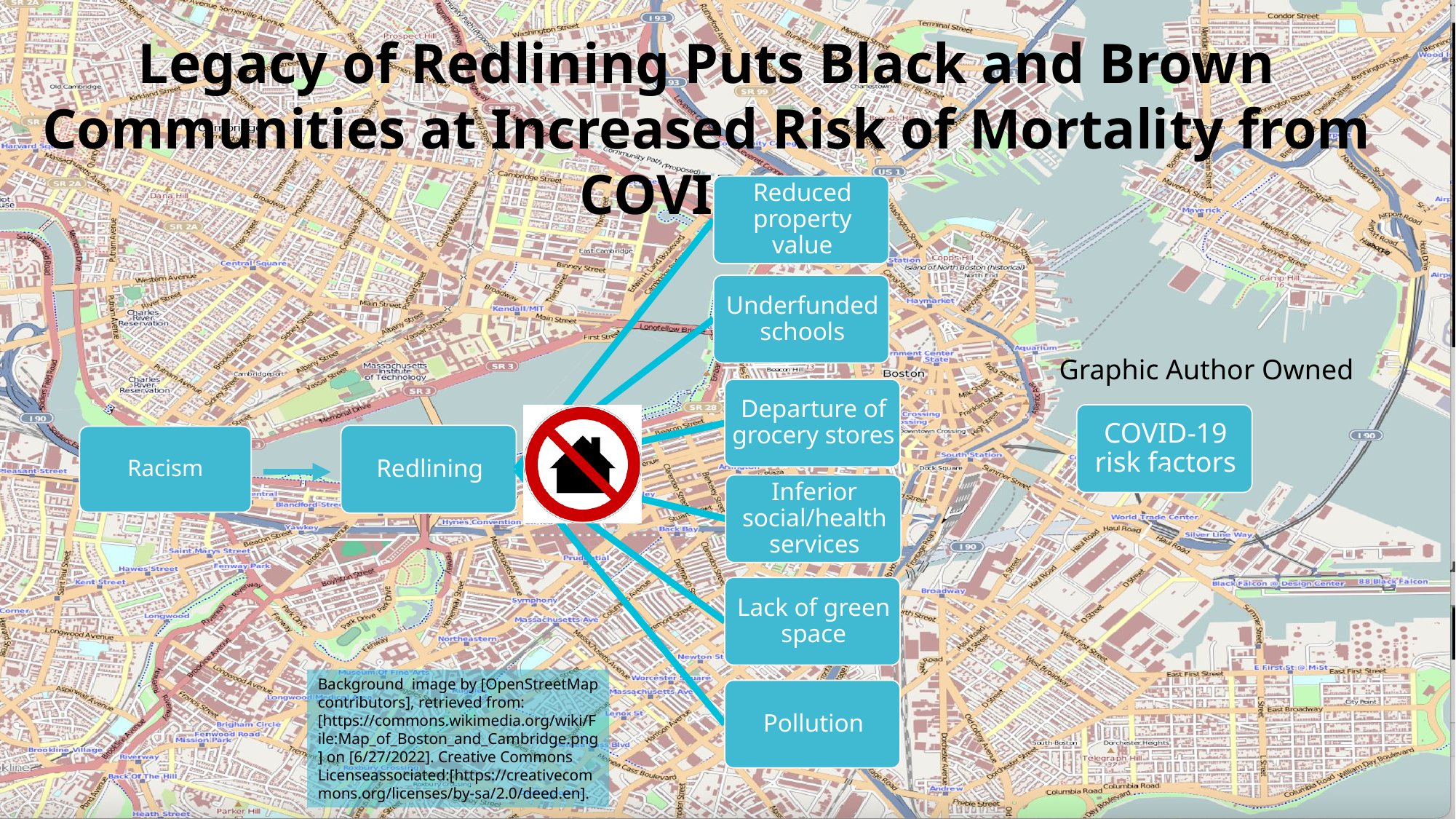

Legacy of Redlining Puts Black and Brown Communities at Increased Risk of Mortality from COVID-19
Graphic Author Owned
Racism
Background image by [OpenStreetMap contributors], retrieved from: [https://commons.wikimedia.org/wiki/File:Map_of_Boston_and_Cambridge.png] on [6/27/2022]. Creative Commons Licenseassociated:[https://creativecommons.org/licenses/by-sa/2.0/deed.en].

## Slide 10
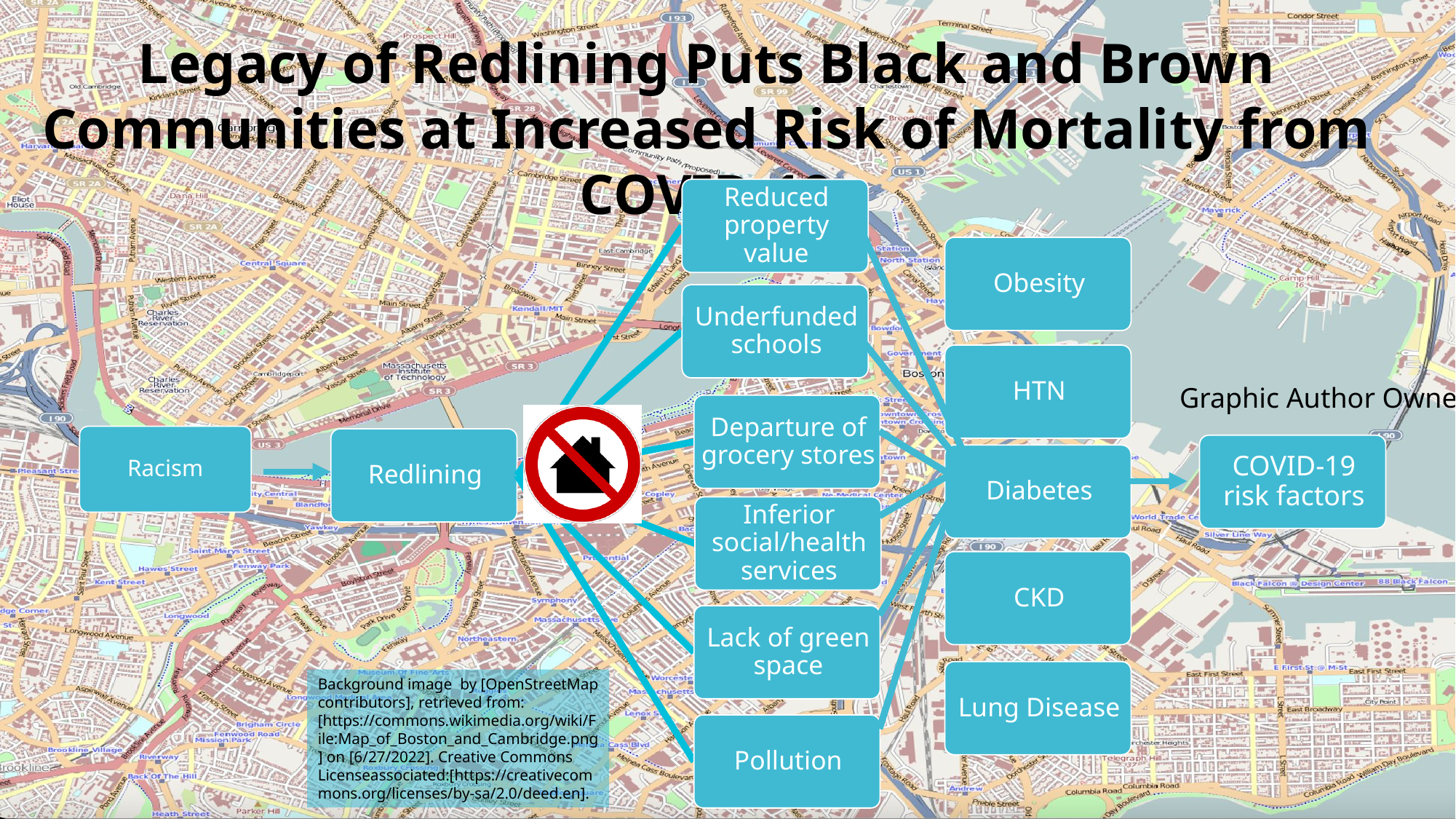

Legacy of Redlining Puts Black and Brown Communities at Increased Risk of Mortality from COVID-19
Graphic Author Owned
Racism
Background image by [OpenStreetMap contributors], retrieved from: [https://commons.wikimedia.org/wiki/File:Map_of_Boston_and_Cambridge.png] on [6/27/2022]. Creative Commons Licenseassociated:[https://creativecommons.org/licenses/by-sa/2.0/deed.en].

## Slide 11
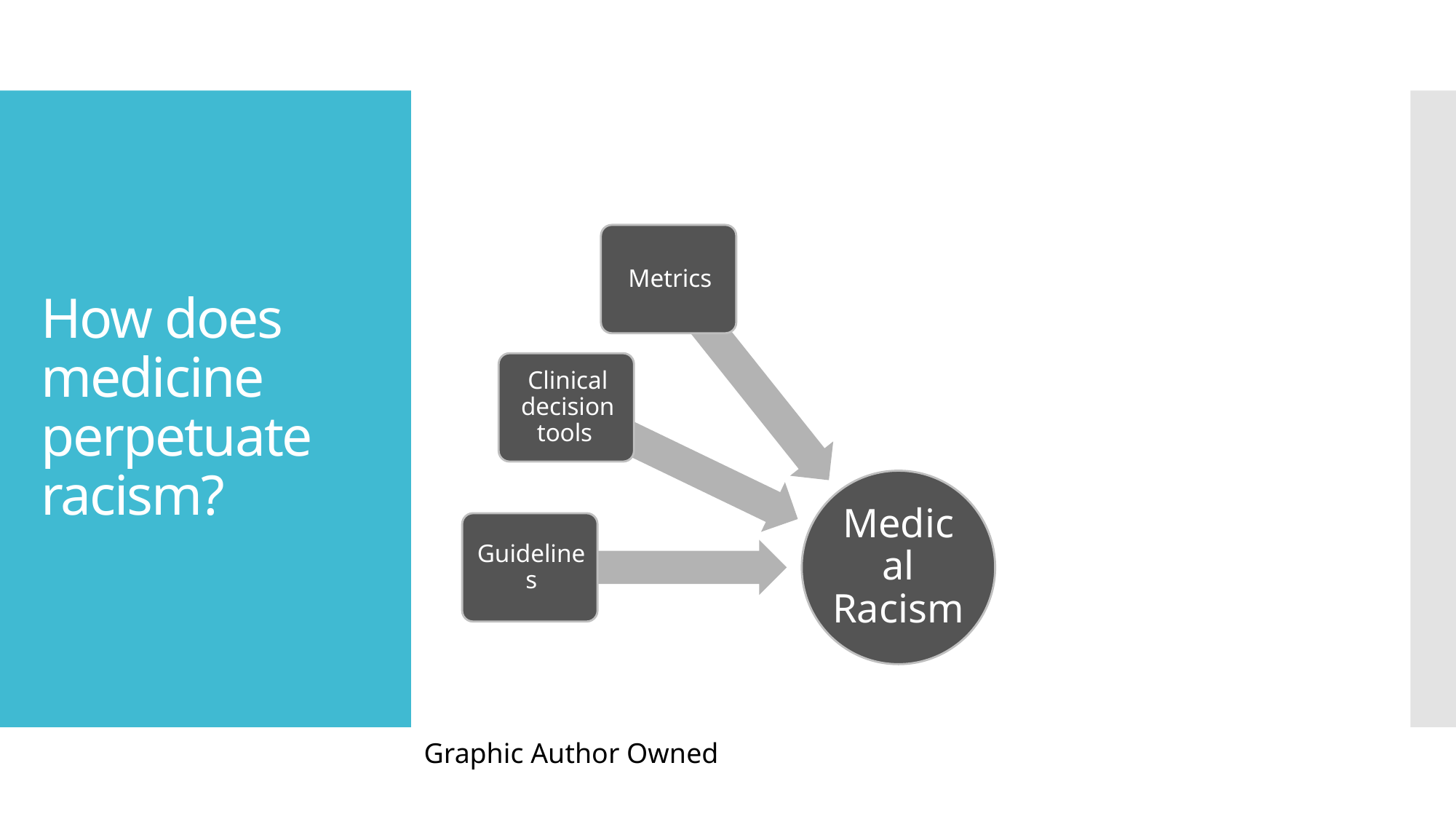

# How does medicine perpetuate racism?
Graphic Author Owned

## Slide 12
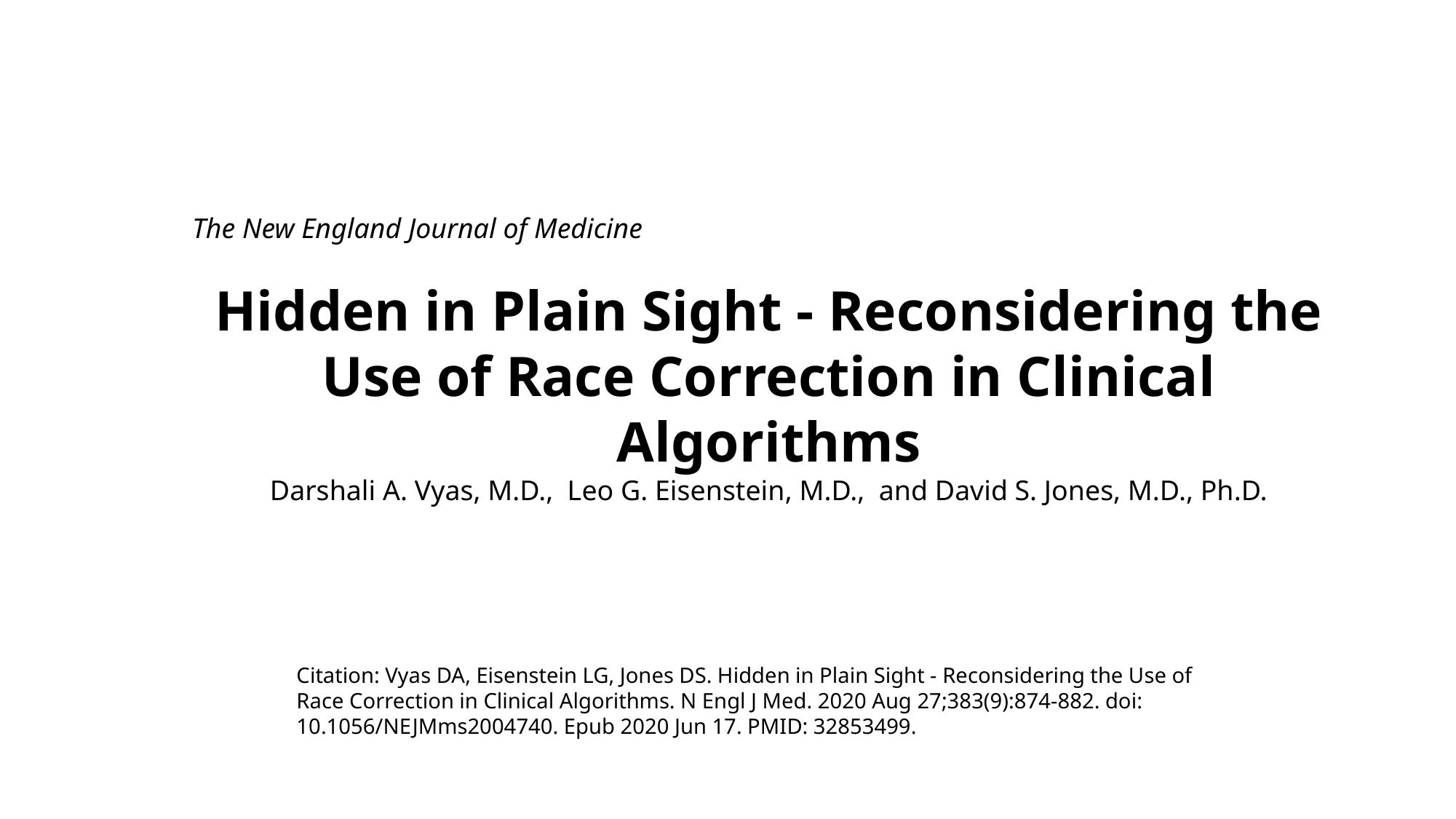

The New England Journal of Medicine
Hidden in Plain Sight - Reconsidering the Use of Race Correction in Clinical Algorithms
Darshali A. Vyas, M.D.,  Leo G. Eisenstein, M.D.,  and David S. Jones, M.D., Ph.D.
Citation: Vyas DA, Eisenstein LG, Jones DS. Hidden in Plain Sight - Reconsidering the Use of Race Correction in Clinical Algorithms. N Engl J Med. 2020 Aug 27;383(9):874-882. doi: 10.1056/NEJMms2004740. Epub 2020 Jun 17. PMID: 32853499.

## Slide 13
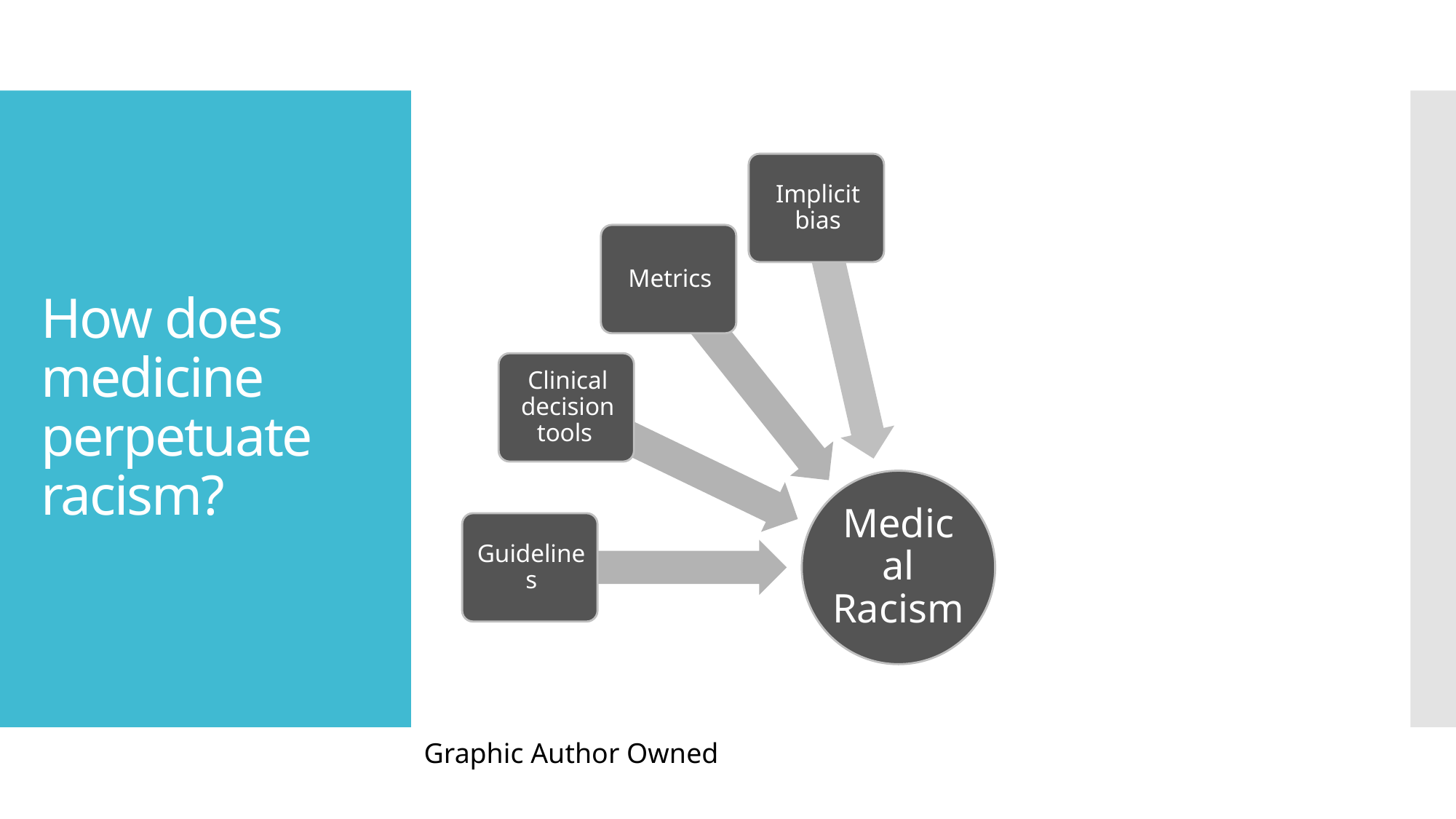

# How does medicine perpetuate racism?
Graphic Author Owned

## Slide 14
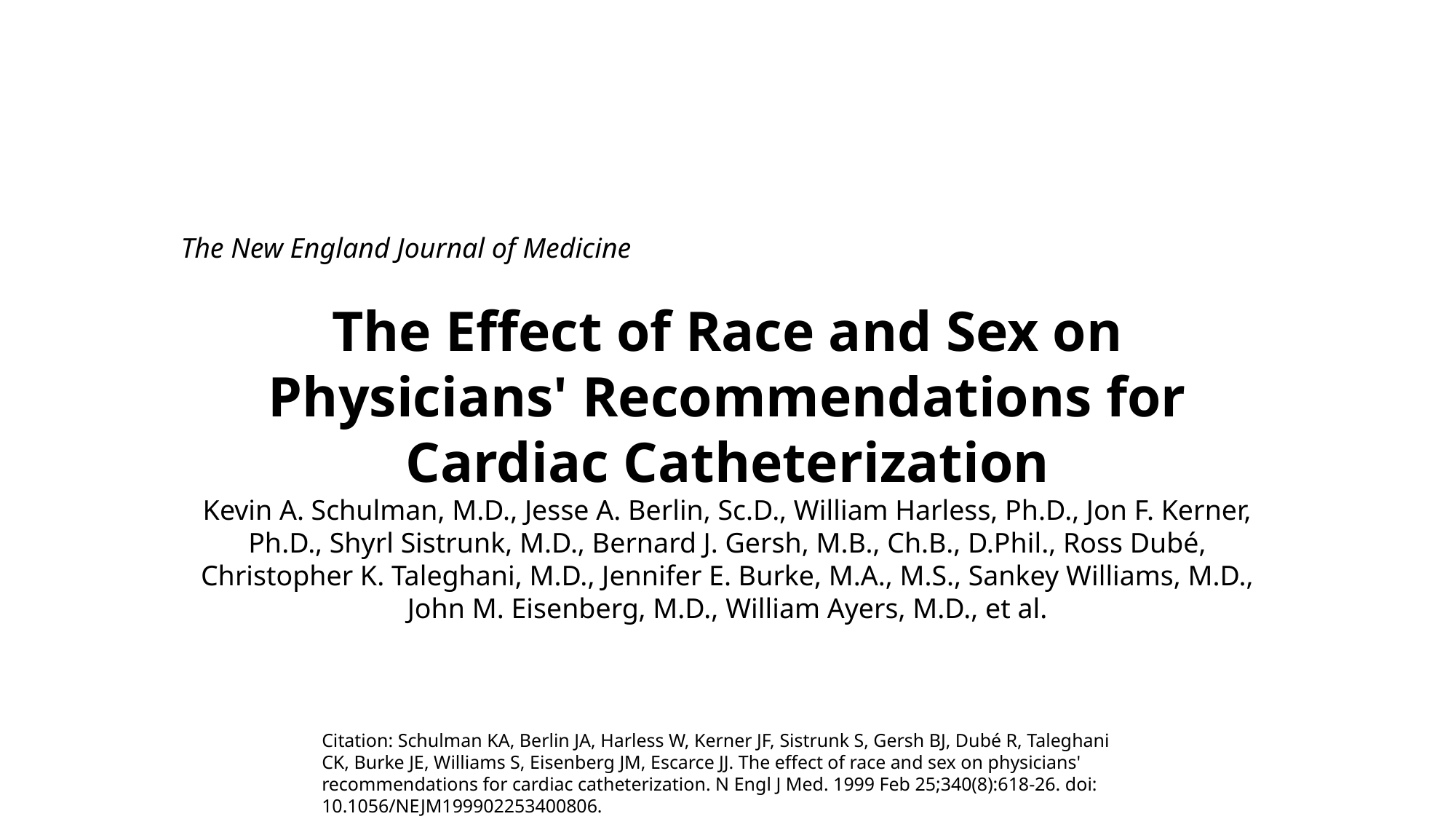

The New England Journal of Medicine
The Effect of Race and Sex on Physicians' Recommendations for Cardiac Catheterization
Kevin A. Schulman, M.D., Jesse A. Berlin, Sc.D., William Harless, Ph.D., Jon F. Kerner, Ph.D., Shyrl Sistrunk, M.D., Bernard J. Gersh, M.B., Ch.B., D.Phil., Ross Dubé, Christopher K. Taleghani, M.D., Jennifer E. Burke, M.A., M.S., Sankey Williams, M.D., John M. Eisenberg, M.D., William Ayers, M.D., et al.
Citation: Schulman KA, Berlin JA, Harless W, Kerner JF, Sistrunk S, Gersh BJ, Dubé R, Taleghani CK, Burke JE, Williams S, Eisenberg JM, Escarce JJ. The effect of race and sex on physicians' recommendations for cardiac catheterization. N Engl J Med. 1999 Feb 25;340(8):618-26. doi: 10.1056/NEJM199902253400806.

## Slide 15
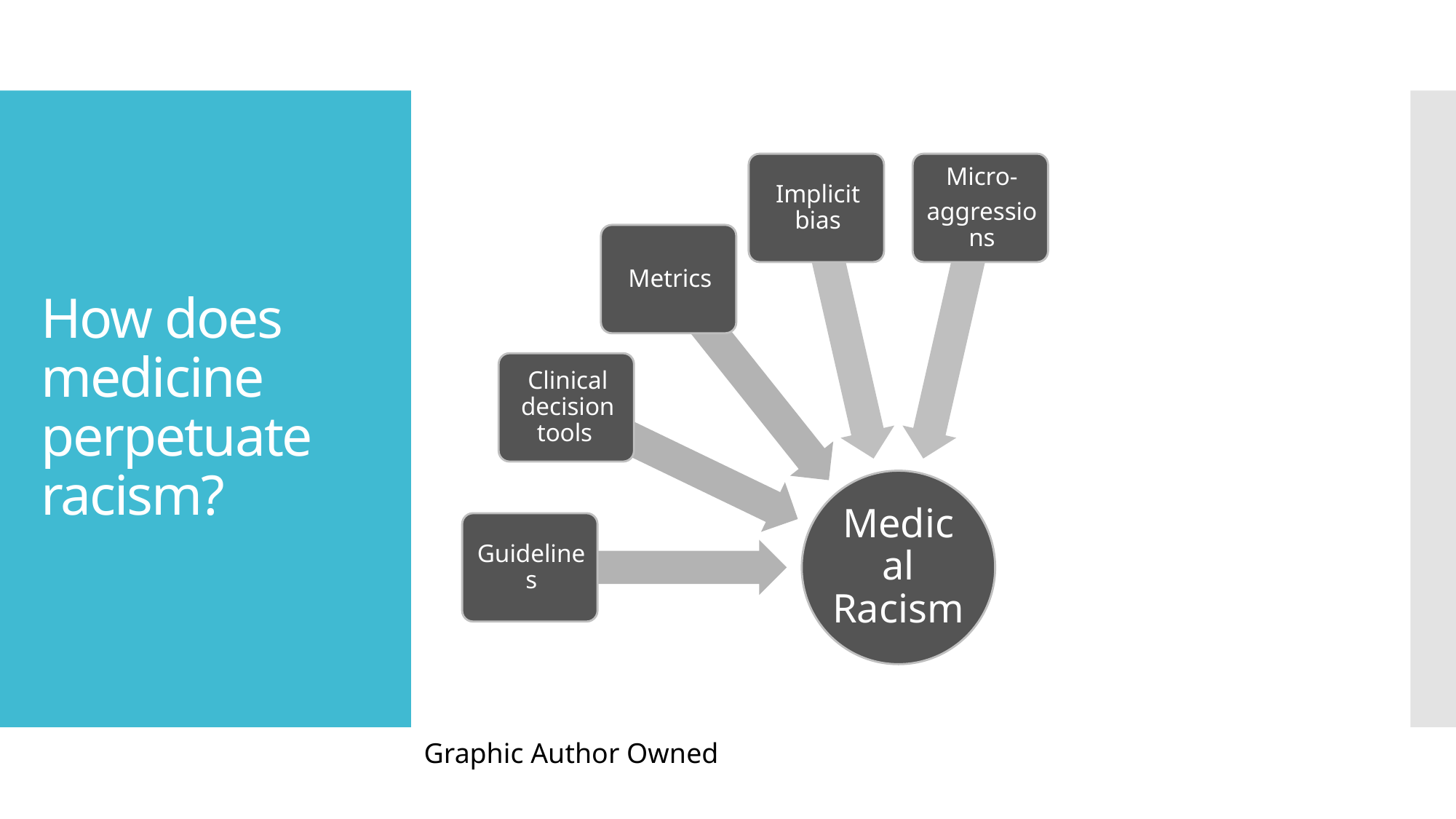

# How does medicine perpetuate racism?
Graphic Author Owned

## Slide 16
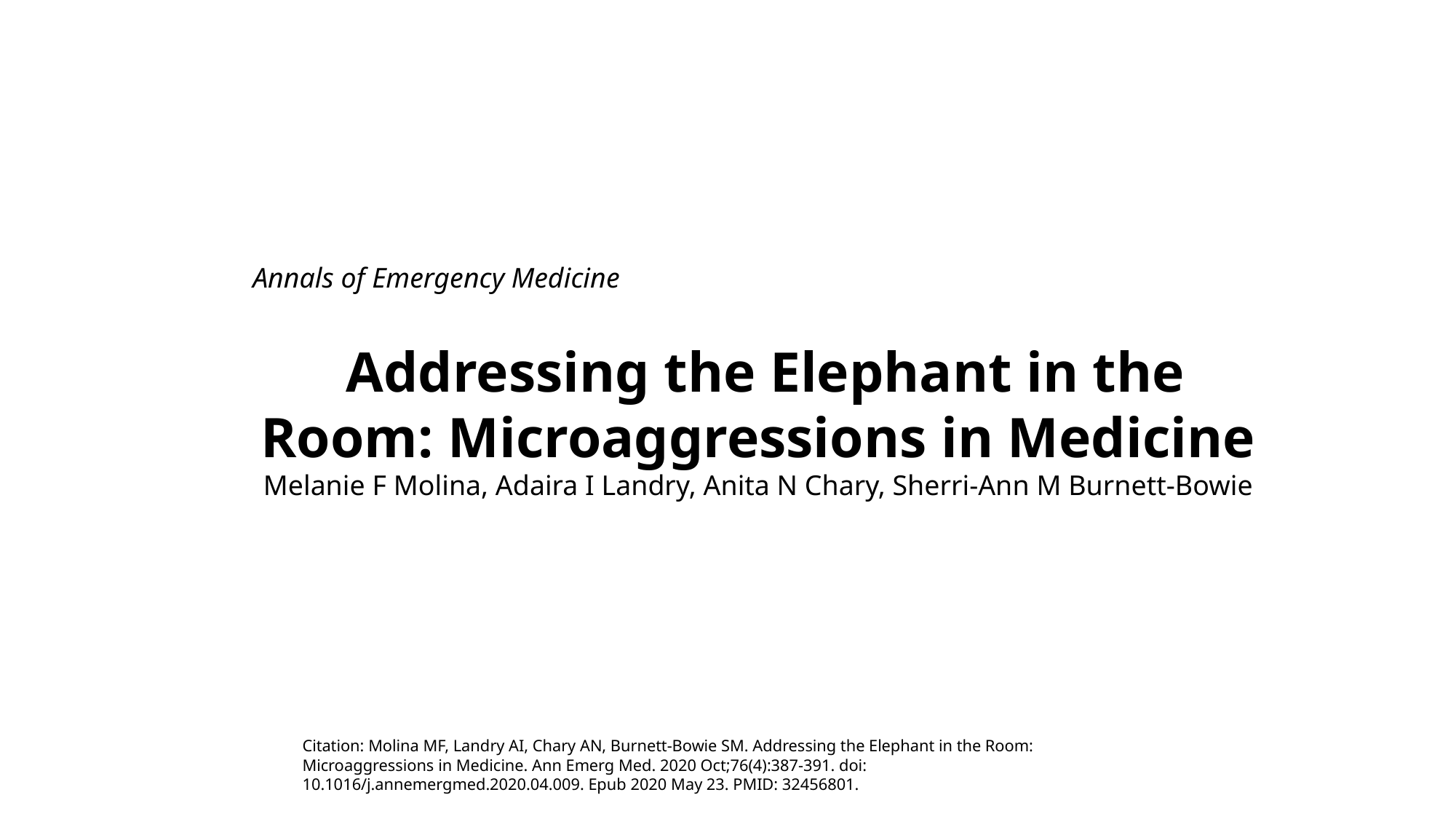

Annals of Emergency Medicine
 Addressing the Elephant in the Room: Microaggressions in Medicine
Melanie F Molina, Adaira I Landry, Anita N Chary, Sherri-Ann M Burnett-Bowie
Citation: Molina MF, Landry AI, Chary AN, Burnett-Bowie SM. Addressing the Elephant in the Room: Microaggressions in Medicine. Ann Emerg Med. 2020 Oct;76(4):387-391. doi: 10.1016/j.annemergmed.2020.04.009. Epub 2020 May 23. PMID: 32456801.

## Slide 17
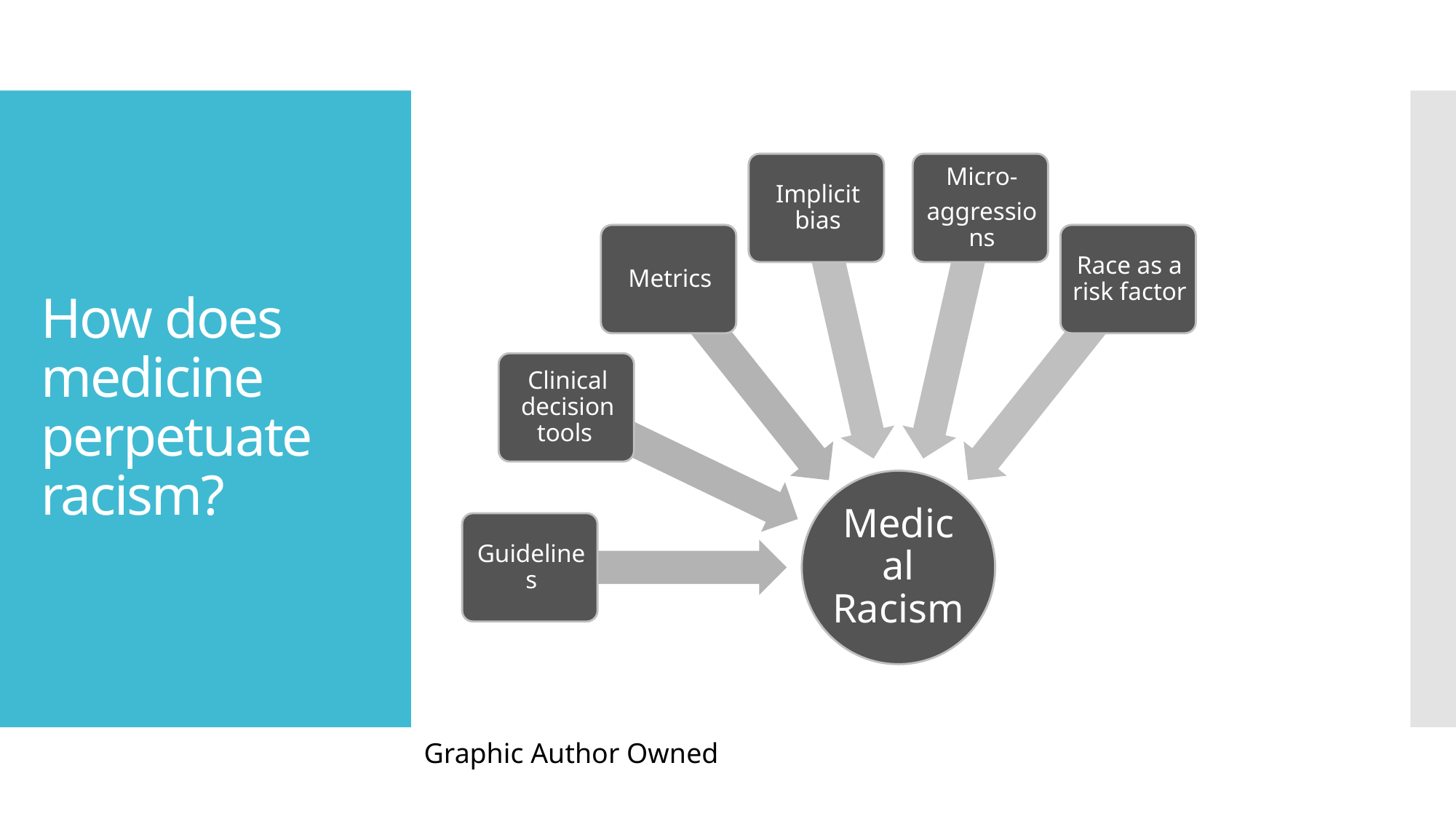

# How does medicine perpetuate racism?
Graphic Author Owned

## Slide 18
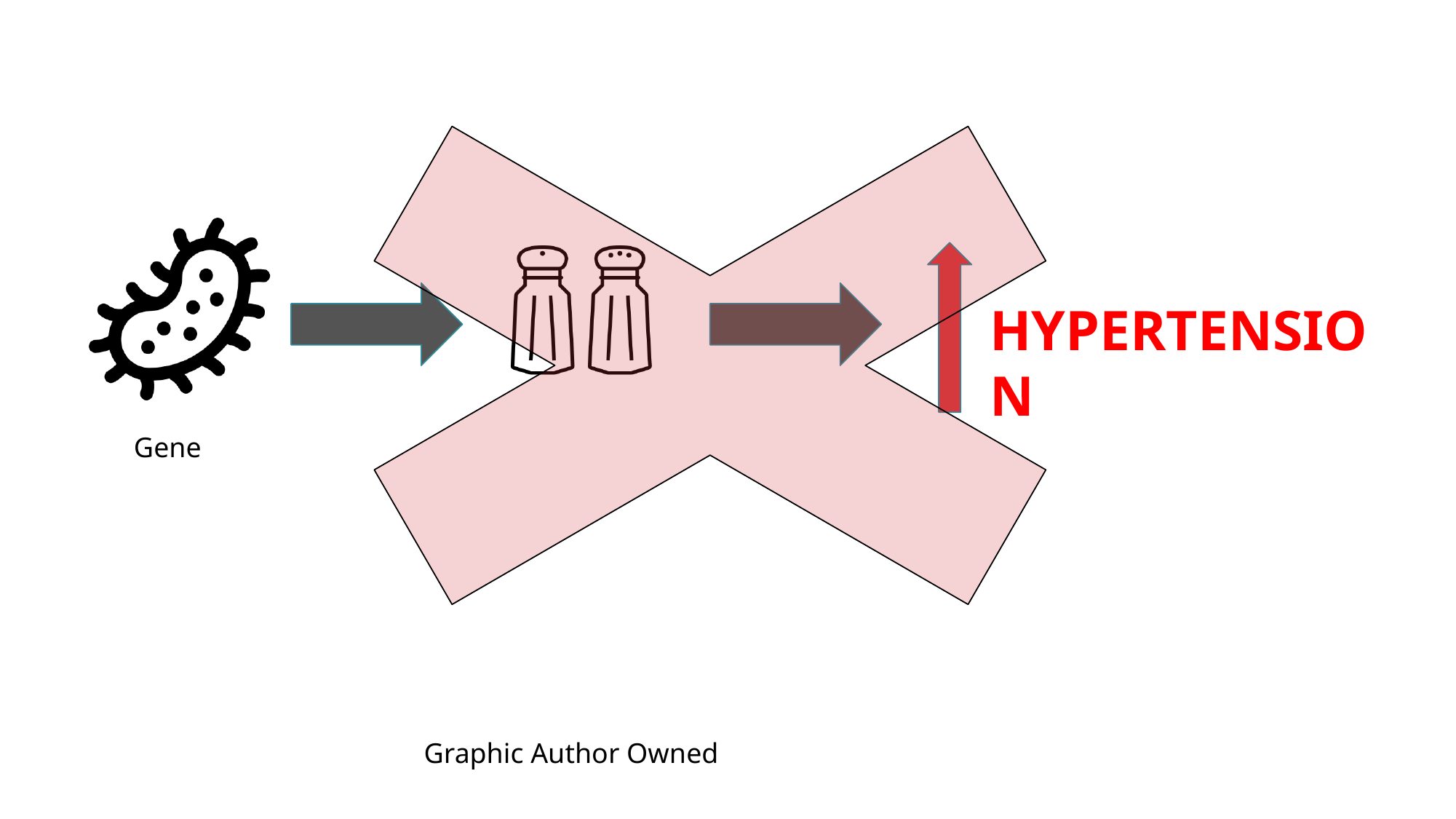

HYPERTENSION
Gene
Graphic Author Owned

## Slide 19
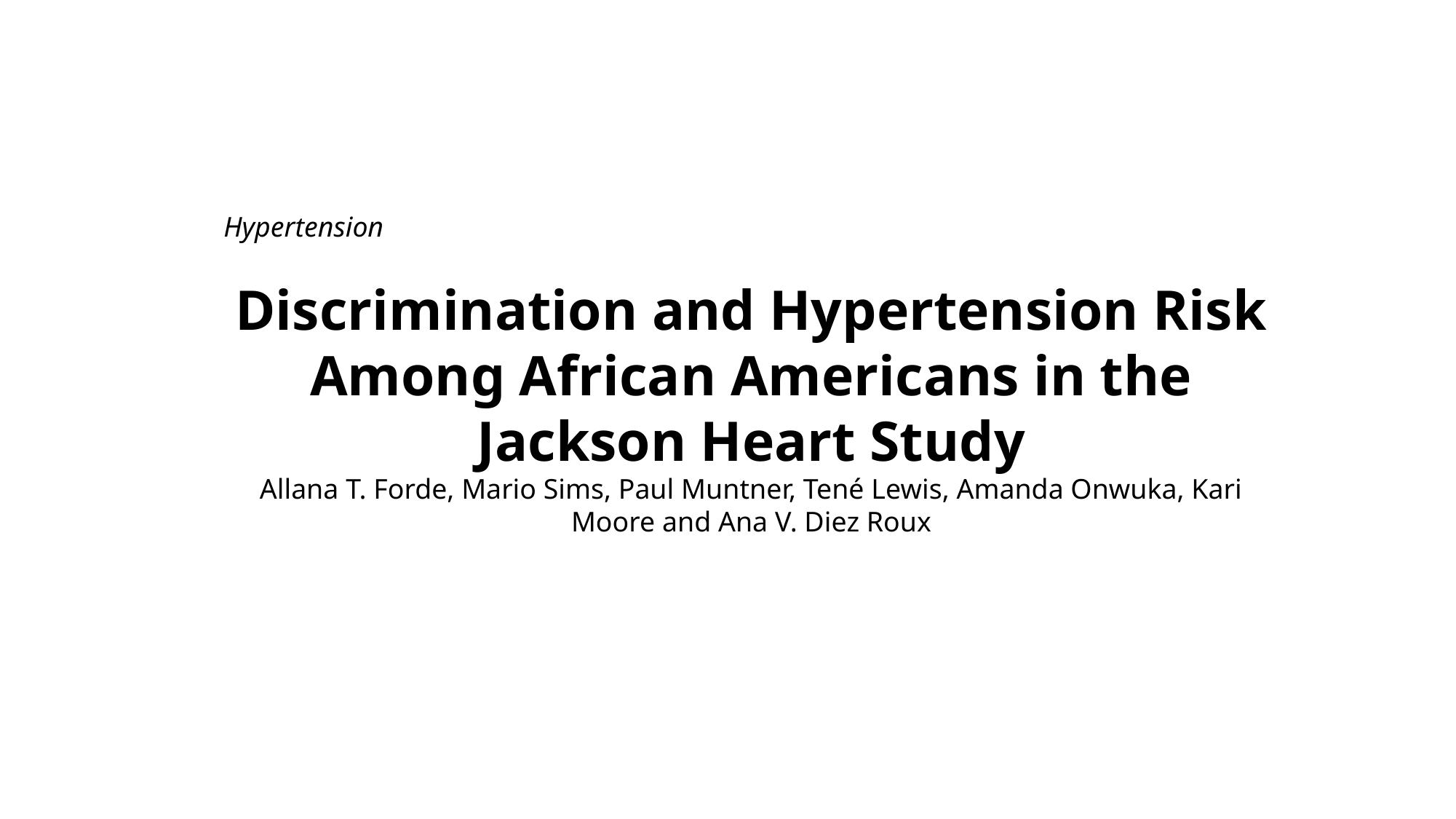

Hypertension
Discrimination and Hypertension Risk Among African Americans in the Jackson Heart Study
Allana T. Forde, Mario Sims, Paul Muntner, Tené Lewis, Amanda Onwuka, Kari Moore and Ana V. Diez Roux

## Slide 20
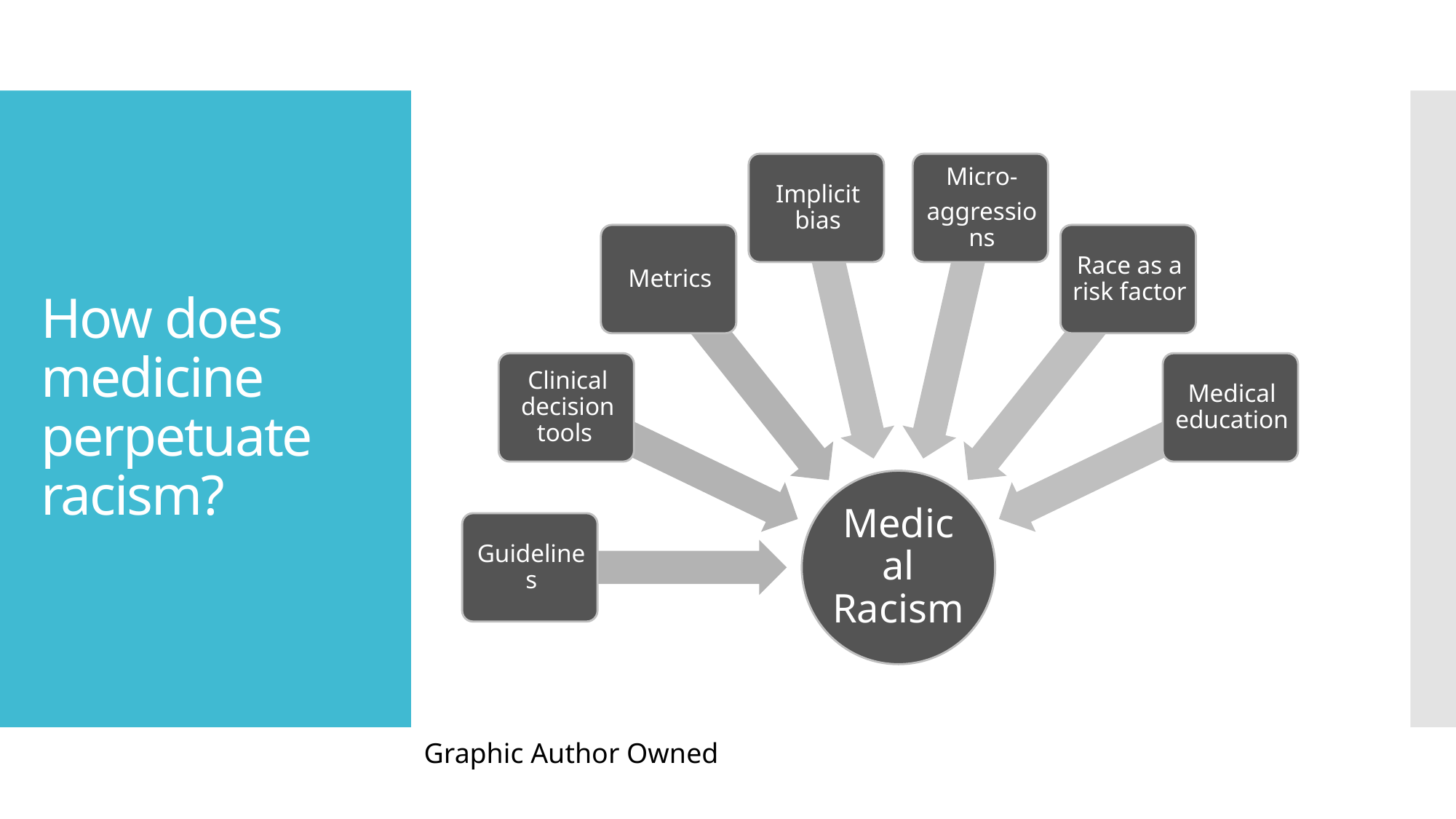

# How does medicine perpetuate racism?
Graphic Author Owned

## Slide 21
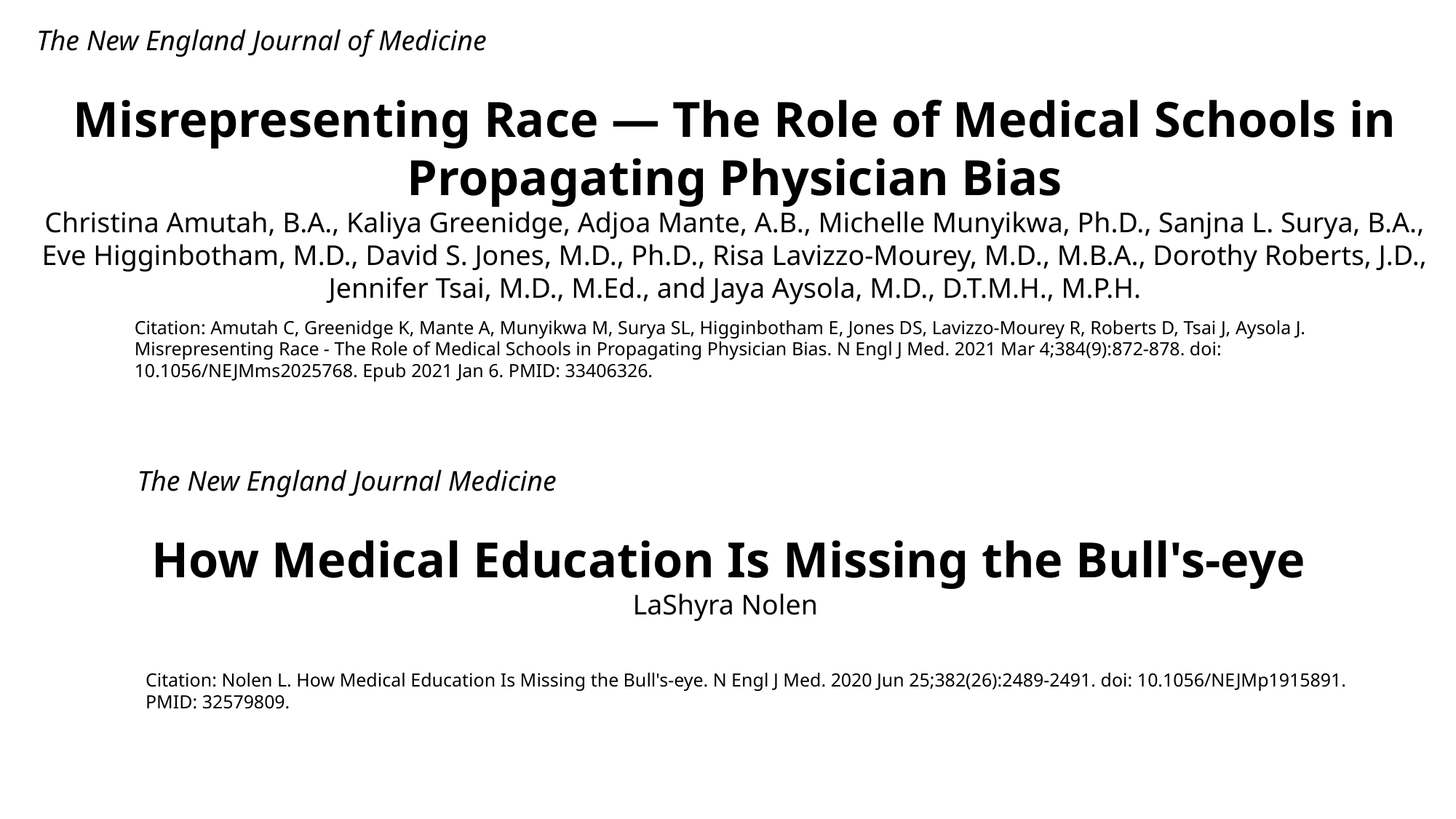

The New England Journal of Medicine
Misrepresenting Race — The Role of Medical Schools in Propagating Physician Bias
Christina Amutah, B.A., Kaliya Greenidge, Adjoa Mante, A.B., Michelle Munyikwa, Ph.D., Sanjna L. Surya, B.A., Eve Higginbotham, M.D., David S. Jones, M.D., Ph.D., Risa Lavizzo-Mourey, M.D., M.B.A., Dorothy Roberts, J.D., Jennifer Tsai, M.D., M.Ed., and Jaya Aysola, M.D., D.T.M.H., M.P.H.
Citation: Amutah C, Greenidge K, Mante A, Munyikwa M, Surya SL, Higginbotham E, Jones DS, Lavizzo-Mourey R, Roberts D, Tsai J, Aysola J. Misrepresenting Race - The Role of Medical Schools in Propagating Physician Bias. N Engl J Med. 2021 Mar 4;384(9):872-878. doi: 10.1056/NEJMms2025768. Epub 2021 Jan 6. PMID: 33406326.
The New England Journal Medicine
How Medical Education Is Missing the Bull's-eye
LaShyra Nolen
Citation: Nolen L. How Medical Education Is Missing the Bull's-eye. N Engl J Med. 2020 Jun 25;382(26):2489-2491. doi: 10.1056/NEJMp1915891. PMID: 32579809.

## Slide 22
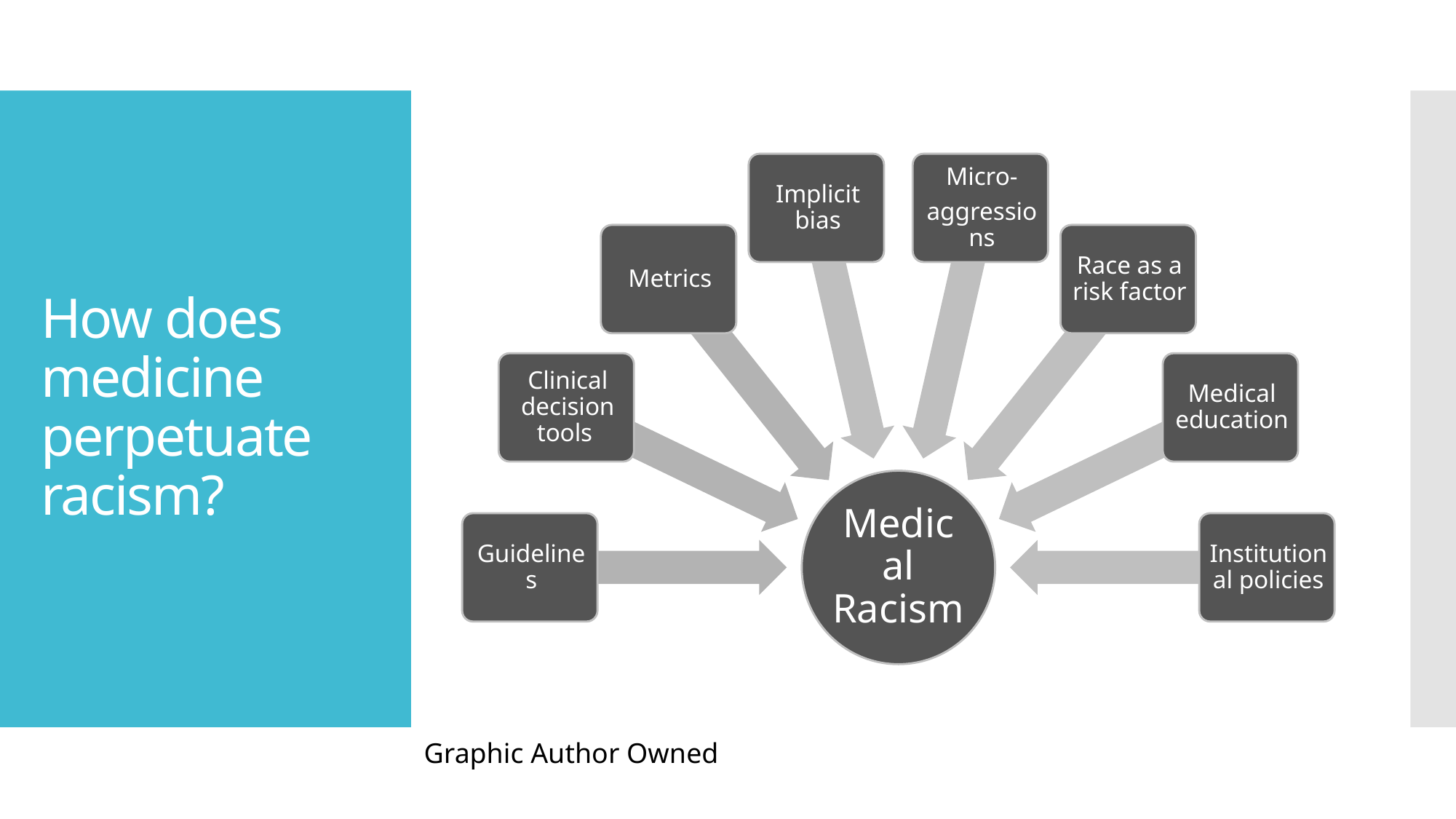

# How does medicine perpetuate racism?
Graphic Author Owned

## Slide 23
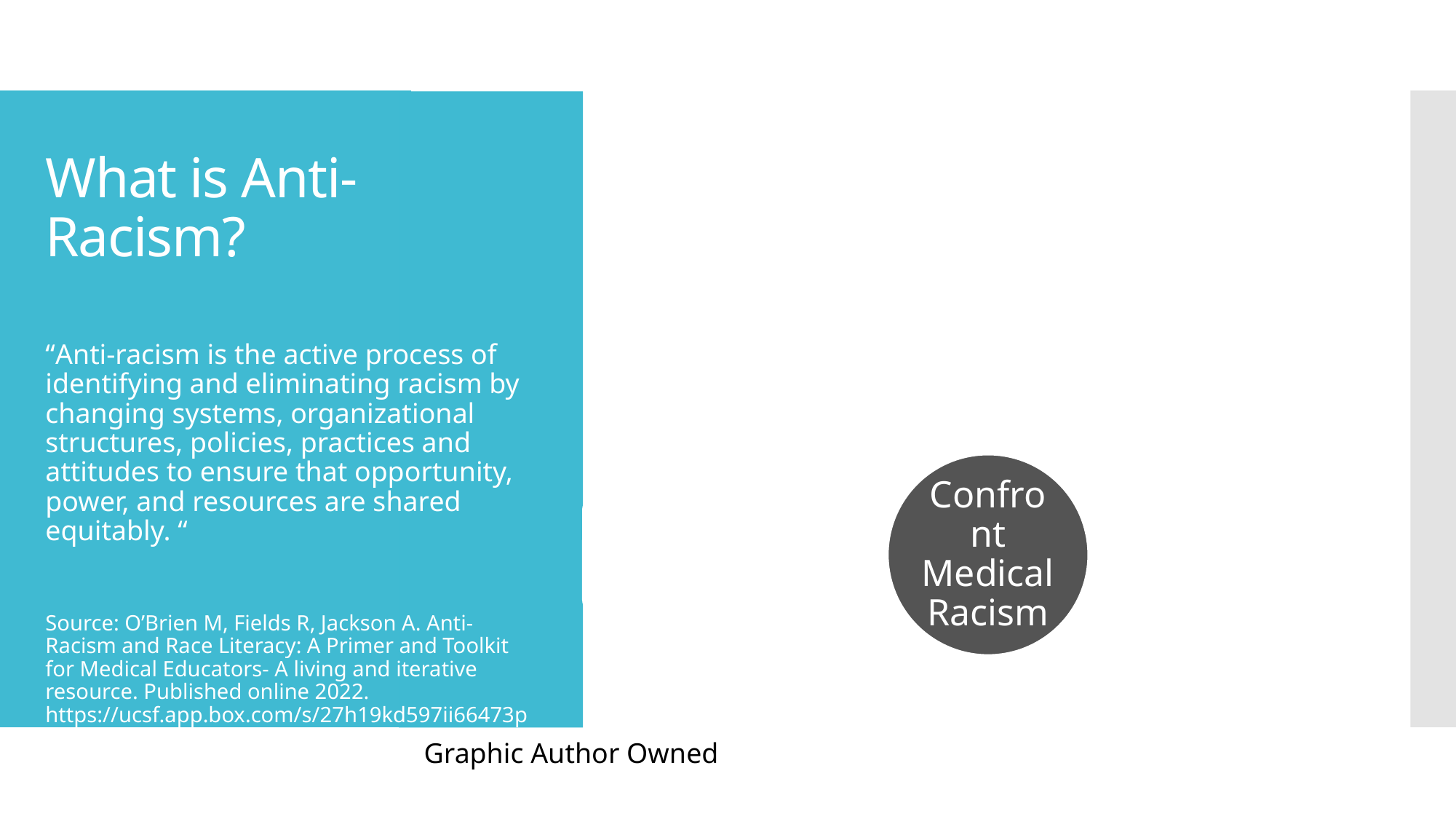

# What is Anti-Racism?
“Anti-racism is the active process of identifying and eliminating racism by changing systems, organizational structures, policies, practices and attitudes to ensure that opportunity, power, and resources are shared equitably. “
Source: O’Brien M, Fields R, Jackson A. Anti-Racism and Race Literacy: A Primer and Toolkit for Medical Educators- A living and iterative resource. Published online 2022. https://ucsf.app.box.com/s/27h19kd597ii66473parki 15u0cgochd
Graphic Author Owned

## Slide 24
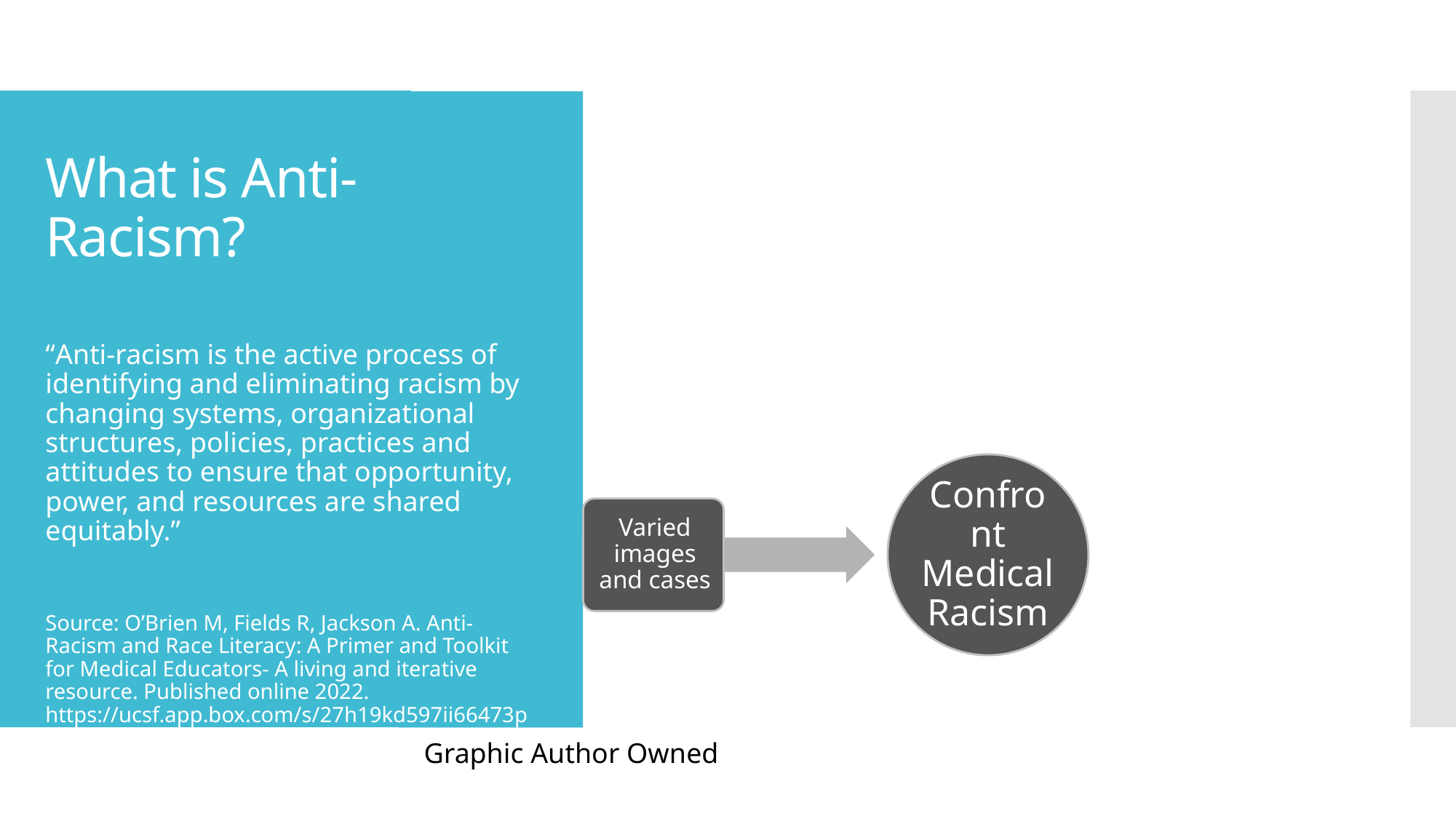

# What is Anti-Racism?
“Anti-racism is the active process of identifying and eliminating racism by changing systems, organizational structures, policies, practices and attitudes to ensure that opportunity, power, and resources are shared equitably.”
Source: O’Brien M, Fields R, Jackson A. Anti-Racism and Race Literacy: A Primer and Toolkit for Medical Educators- A living and iterative resource. Published online 2022. https://ucsf.app.box.com/s/27h19kd597ii66473parki 15u0cgochd
Graphic Author Owned

## Slide 25
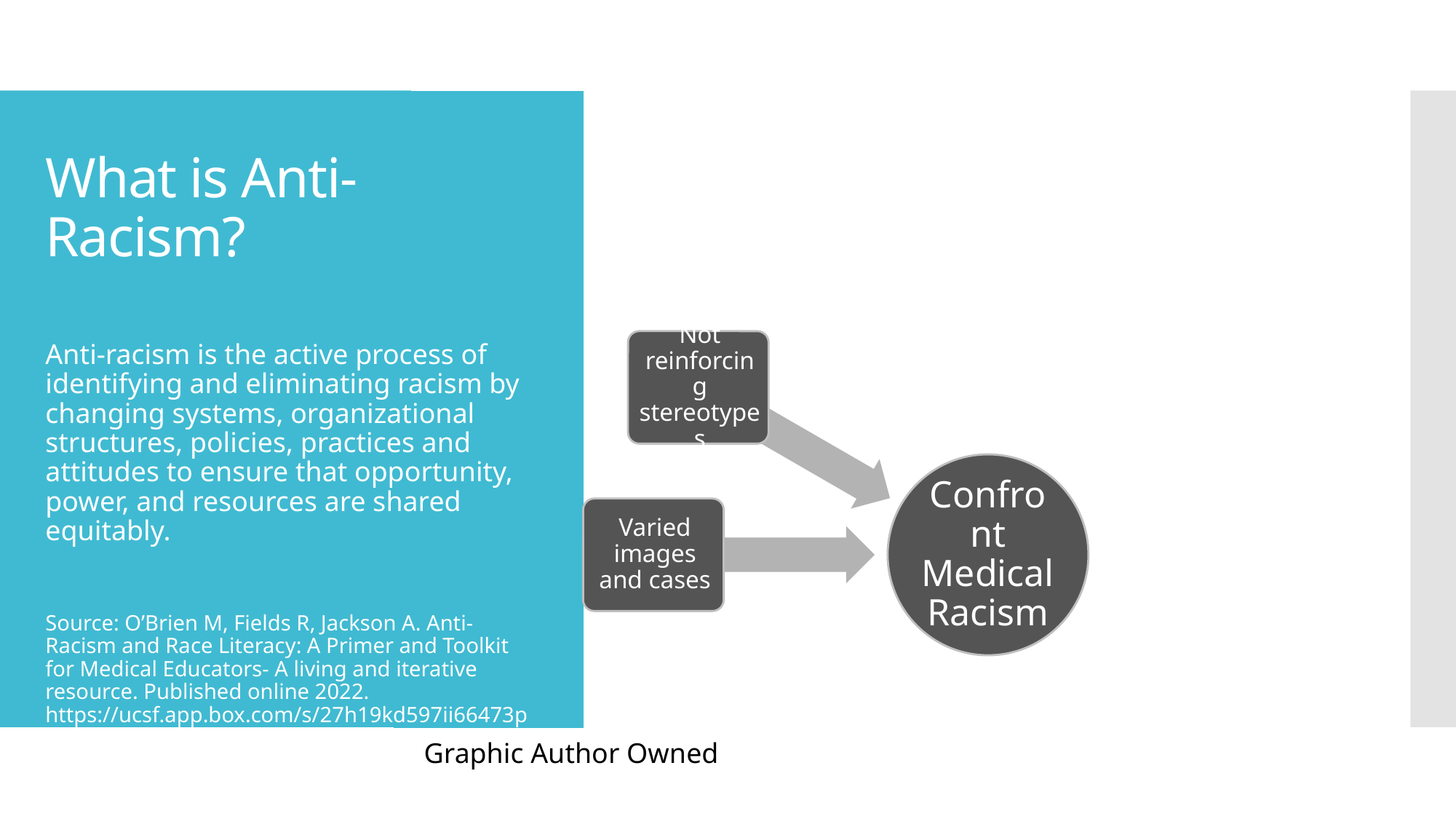

# What is Anti-Racism?
Anti-racism is the active process of identifying and eliminating racism by changing systems, organizational structures, policies, practices and attitudes to ensure that opportunity, power, and resources are shared equitably.
Source: O’Brien M, Fields R, Jackson A. Anti-Racism and Race Literacy: A Primer and Toolkit for Medical Educators- A living and iterative resource. Published online 2022. https://ucsf.app.box.com/s/27h19kd597ii66473parki 15u0cgochd
Graphic Author Owned

## Slide 26
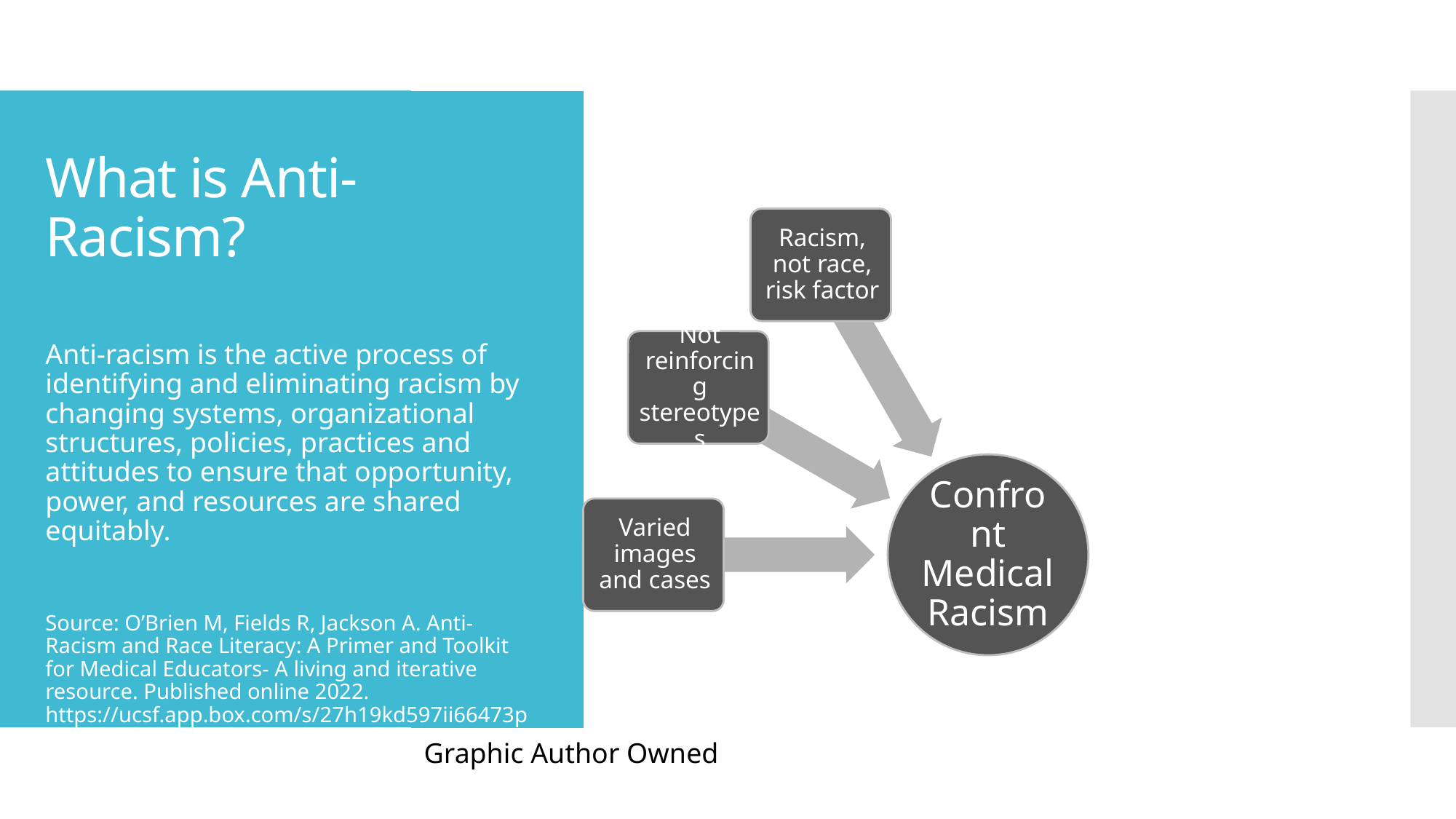

# What is Anti-Racism?
Anti-racism is the active process of identifying and eliminating racism by changing systems, organizational structures, policies, practices and attitudes to ensure that opportunity, power, and resources are shared equitably.
Source: O’Brien M, Fields R, Jackson A. Anti-Racism and Race Literacy: A Primer and Toolkit for Medical Educators- A living and iterative resource. Published online 2022. https://ucsf.app.box.com/s/27h19kd597ii66473parki 15u0cgochd
Graphic Author Owned

## Slide 27
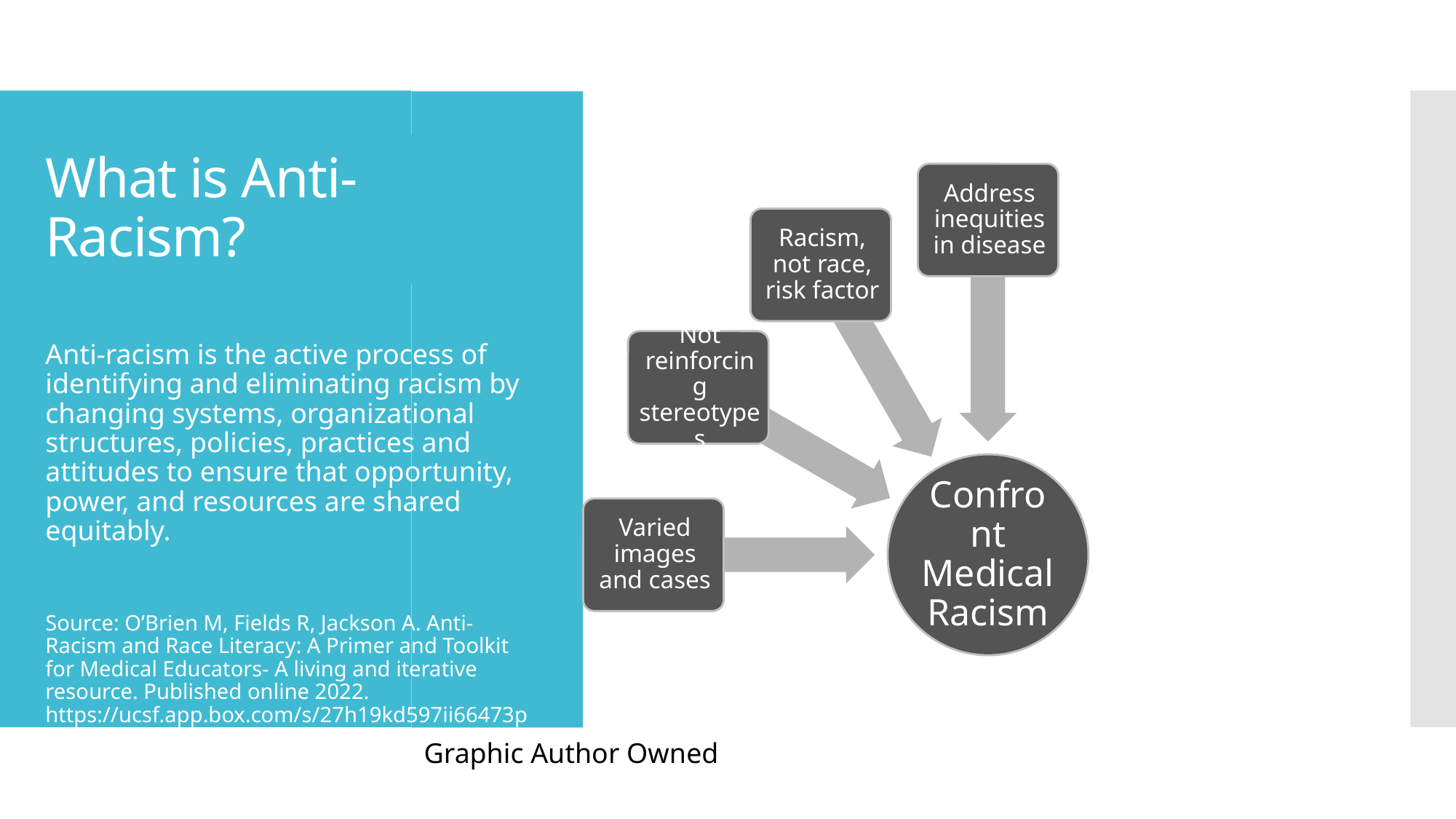

# What is Anti-Racism?
Anti-racism is the active process of identifying and eliminating racism by changing systems, organizational structures, policies, practices and attitudes to ensure that opportunity, power, and resources are shared equitably.
Source: O’Brien M, Fields R, Jackson A. Anti-Racism and Race Literacy: A Primer and Toolkit for Medical Educators- A living and iterative resource. Published online 2022. https://ucsf.app.box.com/s/27h19kd597ii66473parki 15u0cgochd
Graphic Author Owned

## Slide 28
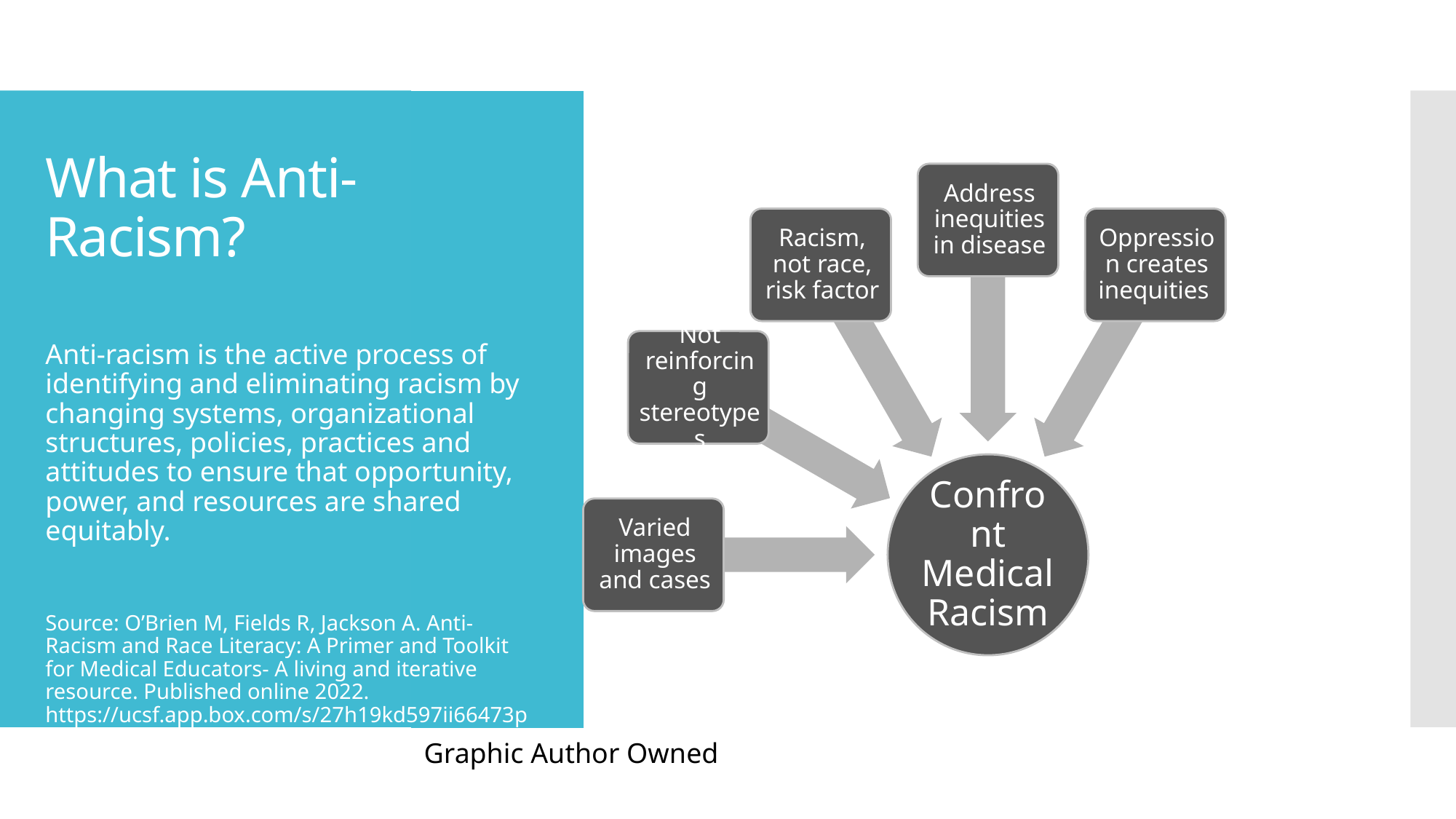

# What is Anti-Racism?
Anti-racism is the active process of identifying and eliminating racism by changing systems, organizational structures, policies, practices and attitudes to ensure that opportunity, power, and resources are shared equitably.
Source: O’Brien M, Fields R, Jackson A. Anti-Racism and Race Literacy: A Primer and Toolkit for Medical Educators- A living and iterative resource. Published online 2022. https://ucsf.app.box.com/s/27h19kd597ii66473parki 15u0cgochd
Graphic Author Owned

## Slide 29
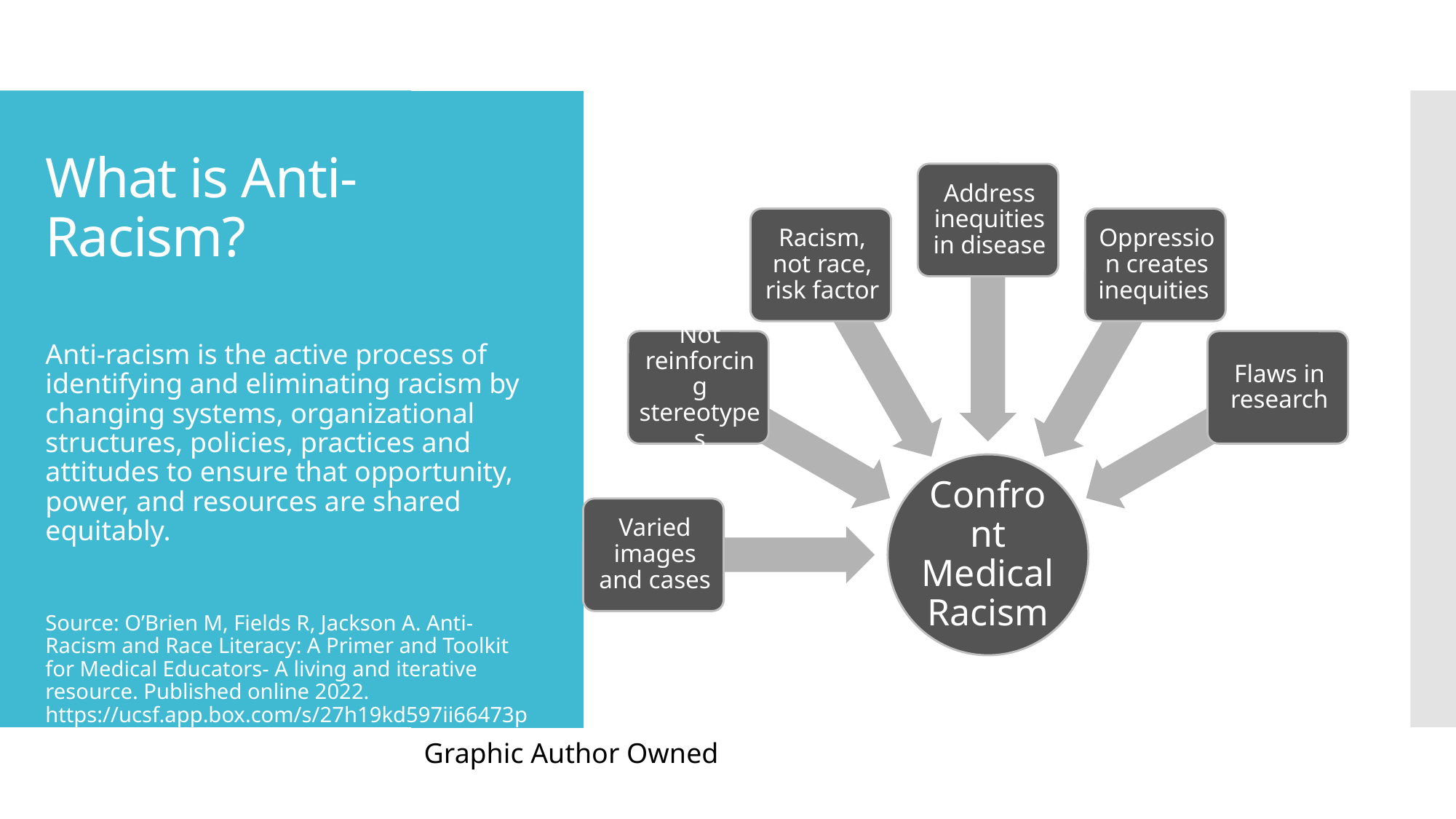

# What is Anti-Racism?
Anti-racism is the active process of identifying and eliminating racism by changing systems, organizational structures, policies, practices and attitudes to ensure that opportunity, power, and resources are shared equitably.
Source: O’Brien M, Fields R, Jackson A. Anti-Racism and Race Literacy: A Primer and Toolkit for Medical Educators- A living and iterative resource. Published online 2022. https://ucsf.app.box.com/s/27h19kd597ii66473parki 15u0cgochd
Graphic Author Owned

## Slide 30
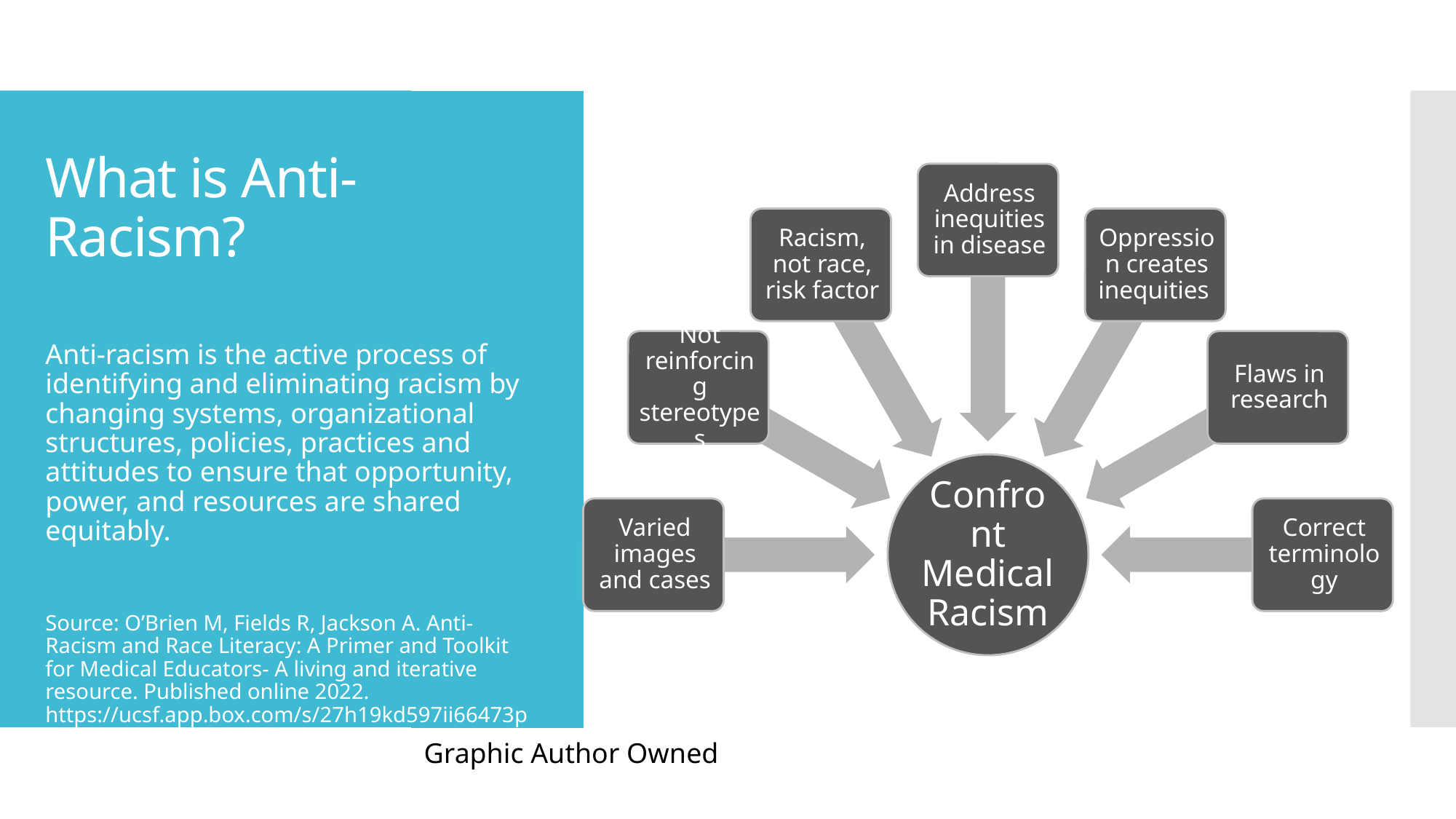

# What is Anti-Racism?
Anti-racism is the active process of identifying and eliminating racism by changing systems, organizational structures, policies, practices and attitudes to ensure that opportunity, power, and resources are shared equitably.
Source: O’Brien M, Fields R, Jackson A. Anti-Racism and Race Literacy: A Primer and Toolkit for Medical Educators- A living and iterative resource. Published online 2022. https://ucsf.app.box.com/s/27h19kd597ii66473parki 15u0cgochd
Graphic Author Owned

## Slide 31
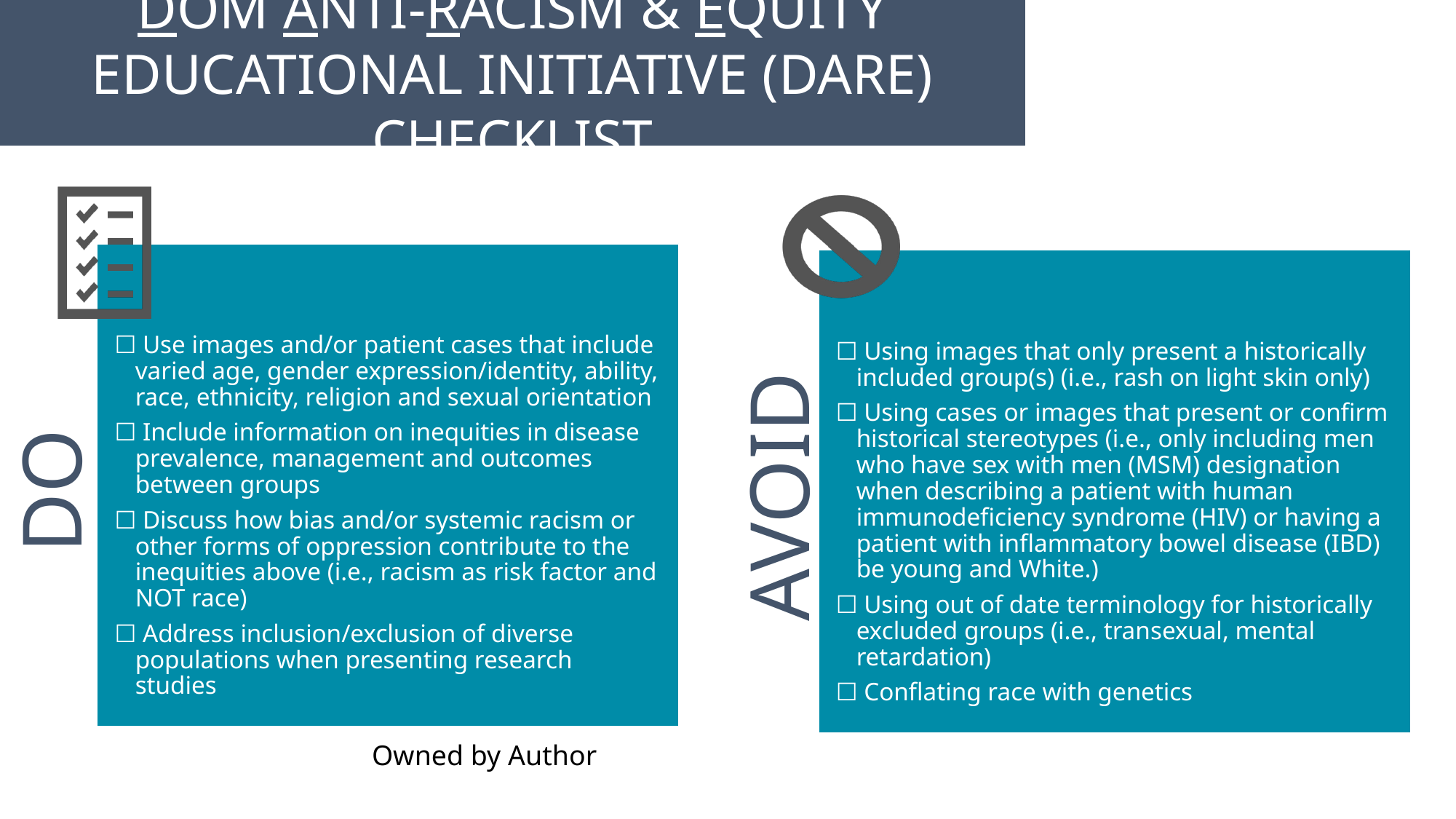

DOM Anti-Racism & Equity Educational Initiative (DARE) Checklist
Owned by Author

## Slide 32
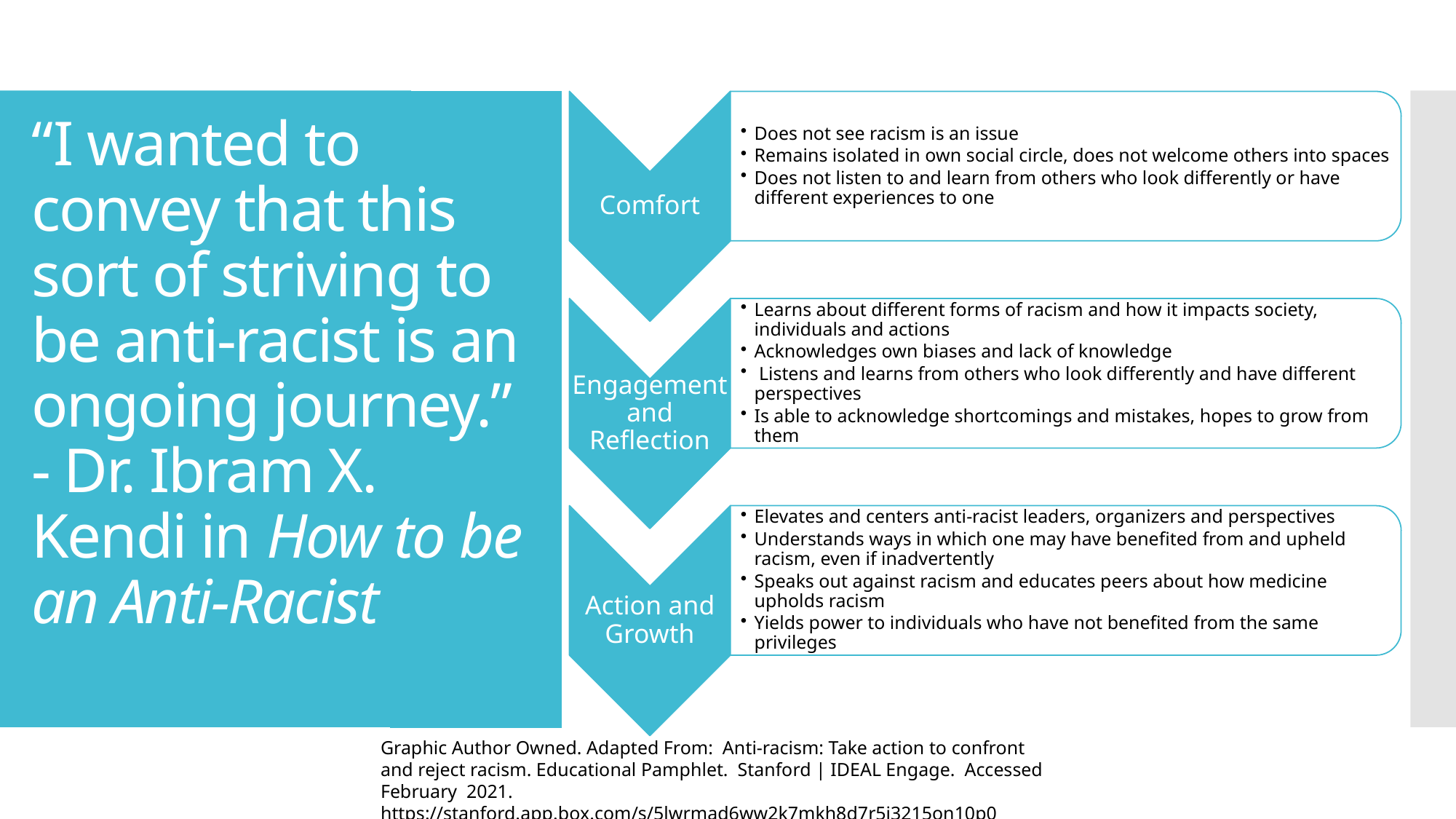

# “I wanted to convey that this sort of striving to be anti-racist is an ongoing journey.” - Dr. Ibram X. Kendi in How to be an Anti-Racist
Graphic Author Owned. Adapted From: Anti-racism: Take action to confront and reject racism. Educational Pamphlet. Stanford | IDEAL Engage. Accessed February 2021. https://stanford.app.box.com/s/5lwrmad6ww2k7mkh8d7r5i3215on10p0

## Slide 33
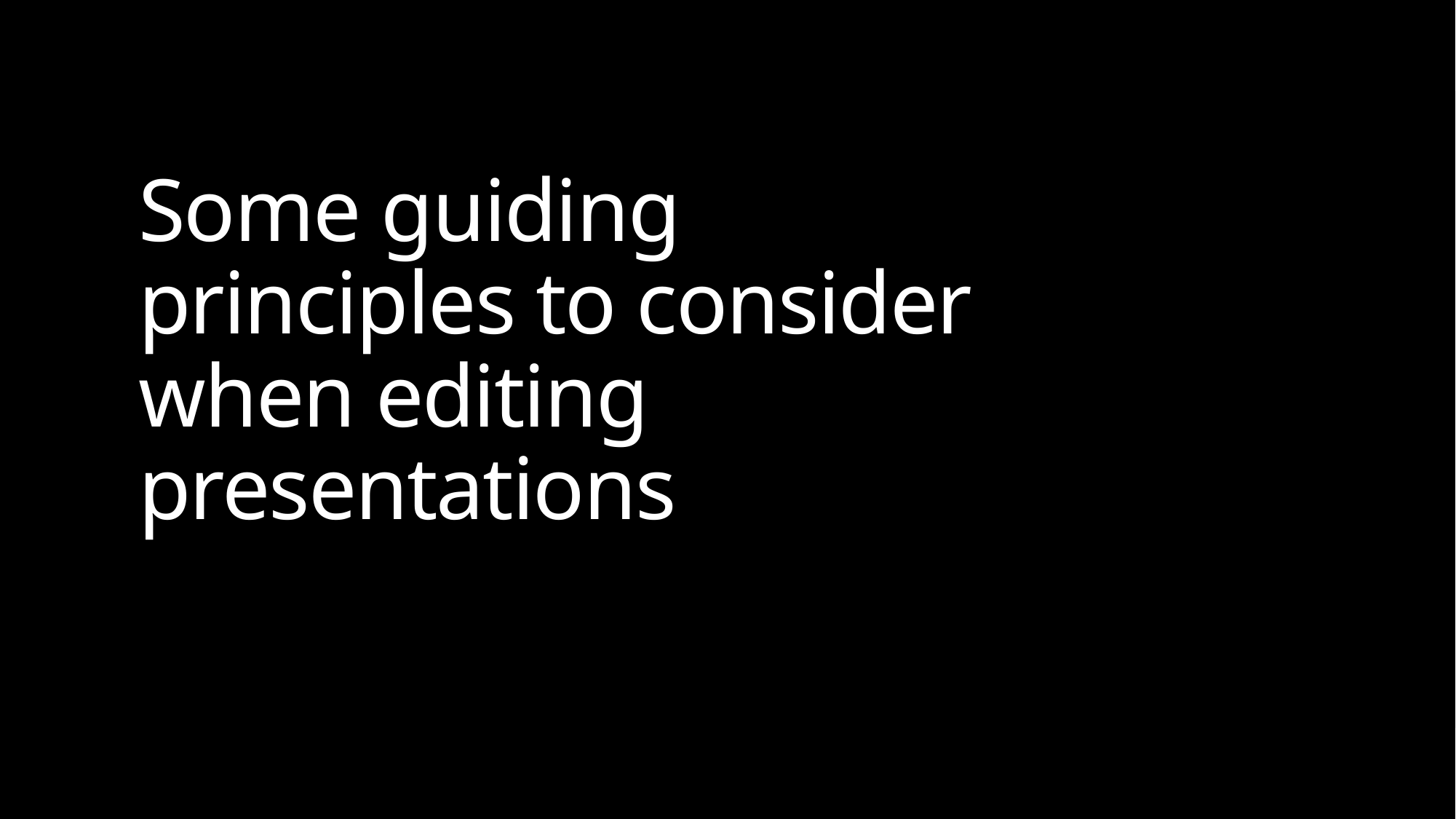

# Some guiding principles to consider when editing presentations

## Slide 34
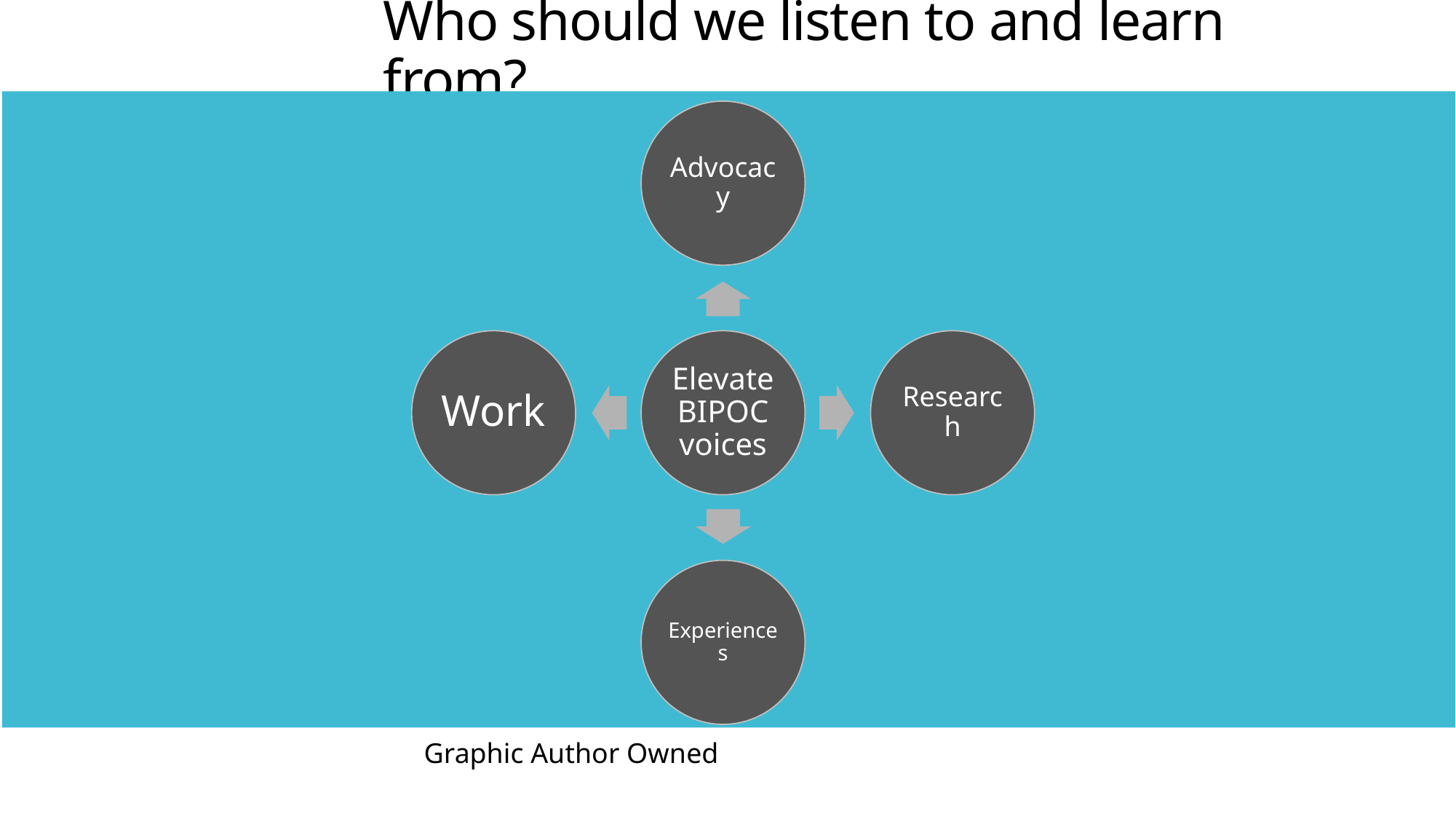

Who should we listen to and learn from?
Graphic Author Owned

## Slide 35
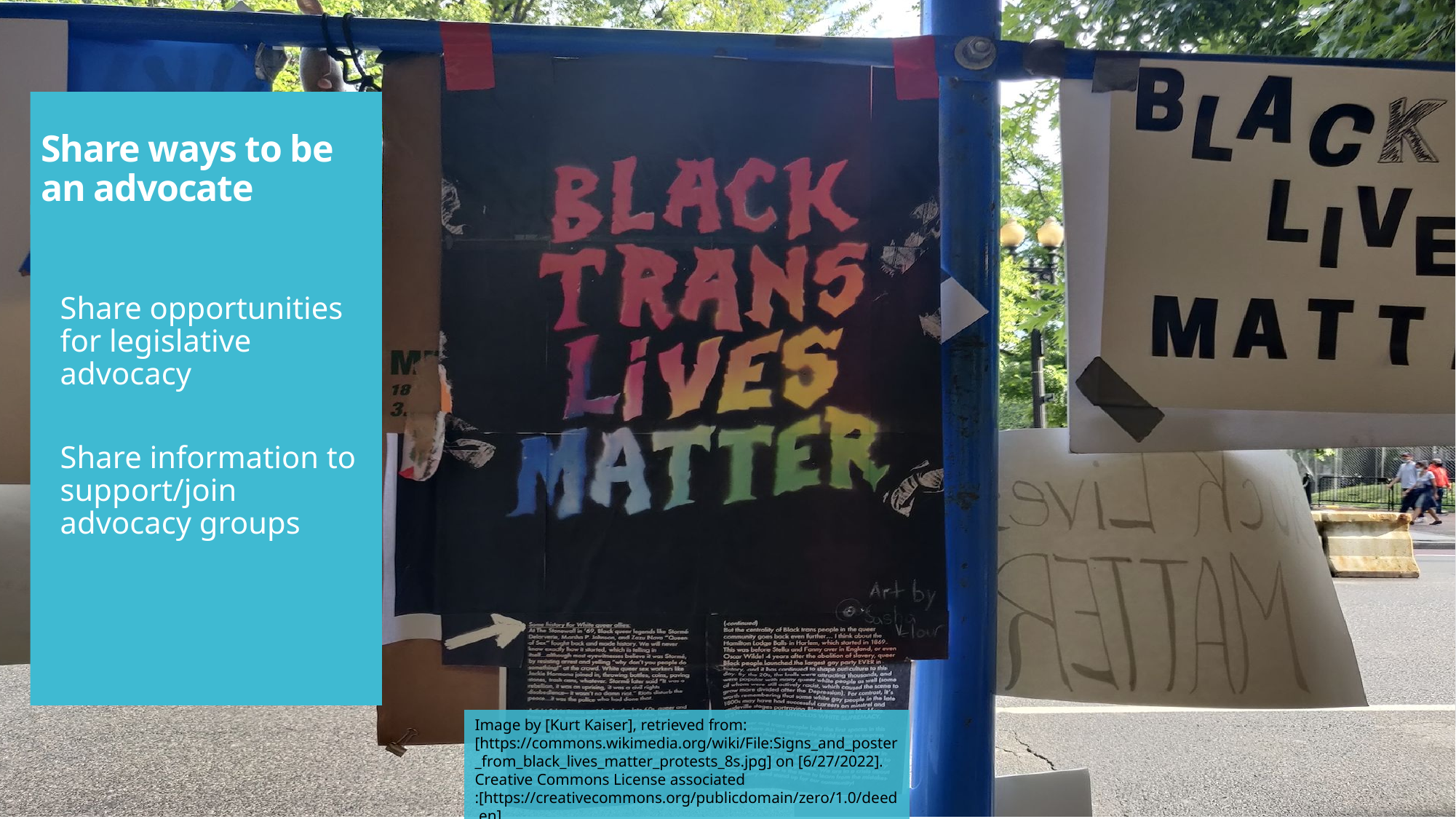

# Share ways to be an advocate
Share opportunities for legislative advocacy
Share information to support/join advocacy groups
Image by [Kurt Kaiser], retrieved from: [https://commons.wikimedia.org/wiki/File:Signs_and_poster_from_black_lives_matter_protests_8s.jpg] on [6/27/2022]. Creative Commons License associated :[https://creativecommons.org/publicdomain/zero/1.0/deed.en].

## Slide 36
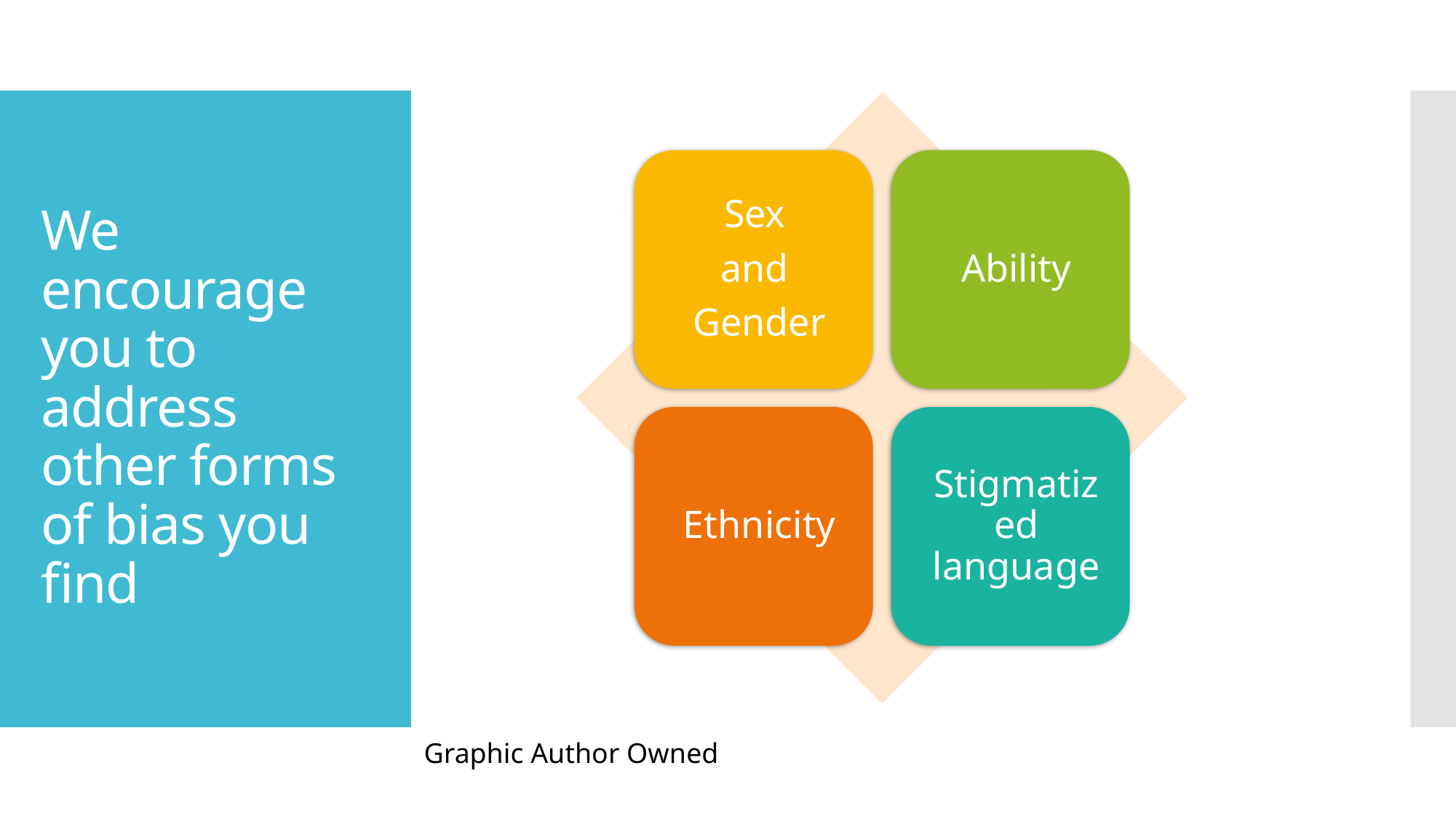

# We encourage you to address other forms of bias you find
Graphic Author Owned

## Slide 37
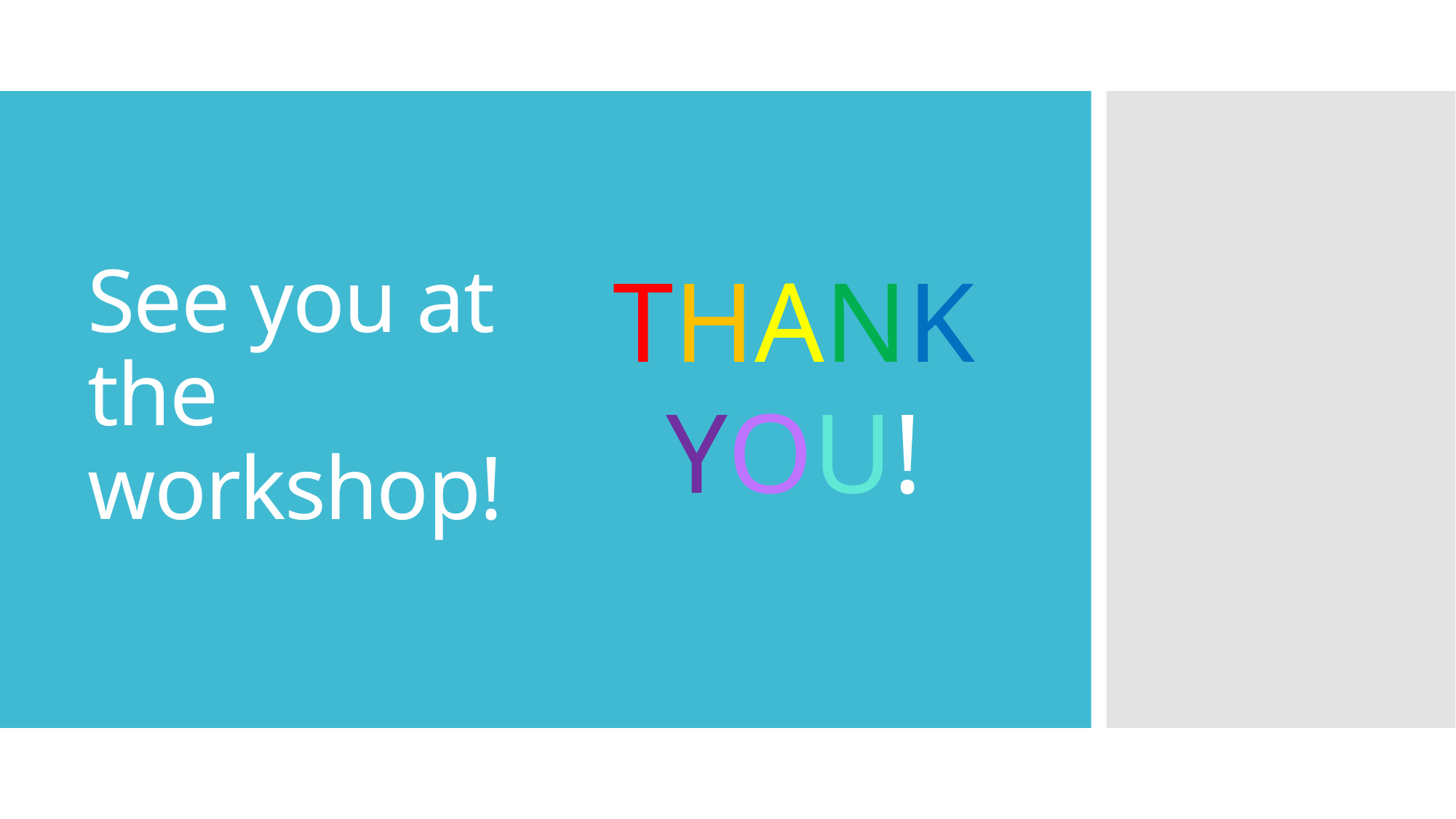

# See you at the workshop!
THANK YOU!

## Slide 38
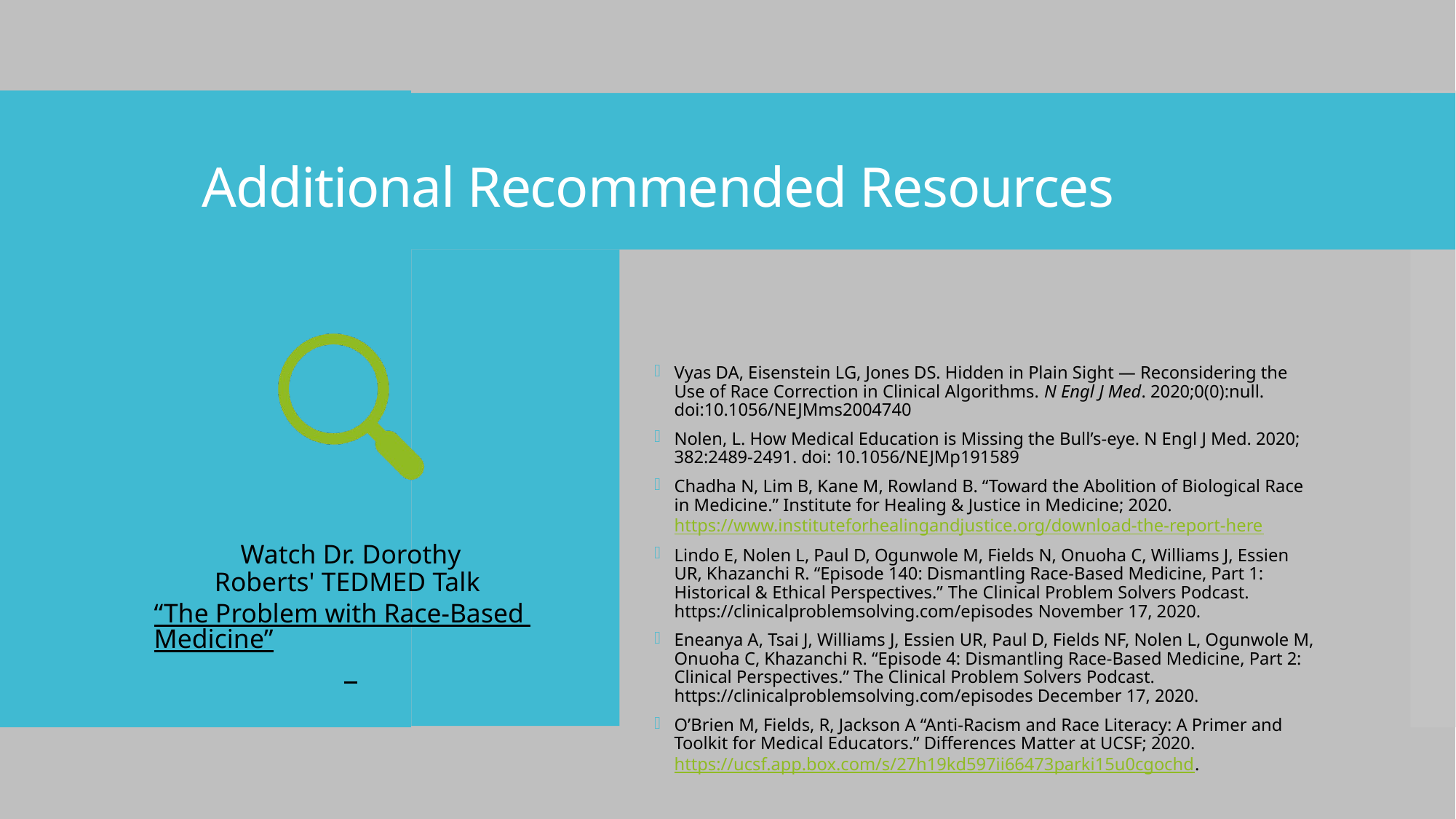

# Additional Recommended Resources
Watch Dr. Dorothy Roberts' TEDMED Talk “The Problem with Race-Based Medicine”
Vyas DA, Eisenstein LG, Jones DS. Hidden in Plain Sight — Reconsidering the Use of Race Correction in Clinical Algorithms. N Engl J Med. 2020;0(0):null. doi:10.1056/NEJMms2004740
Nolen, L. How Medical Education is Missing the Bull’s-eye. N Engl J Med. 2020; 382:2489-2491. doi: 10.1056/NEJMp191589
Chadha N, Lim B, Kane M, Rowland B. “Toward the Abolition of Biological Race in Medicine.” Institute for Healing & Justice in Medicine; 2020. https://www.instituteforhealingandjustice.org/download-the-report-here
Lindo E, Nolen L, Paul D, Ogunwole M, Fields N, Onuoha C, Williams J, Essien UR, Khazanchi R. “Episode 140: Dismantling Race-Based Medicine, Part 1: Historical & Ethical Perspectives.” ​The Clinical Problem Solvers Podcast.​ ​https://clinicalproblemsolving.com/episodes​ November 17, 2020.
Eneanya A, Tsai J, Williams J, Essien UR, Paul D, Fields NF, Nolen L, Ogunwole M, Onuoha C, Khazanchi R. “Episode 4: Dismantling Race-Based Medicine, Part 2: Clinical Perspectives.” The Clinical Problem Solvers Podcast. https://clinicalproblemsolving.com/episodes December 17, 2020.
O’Brien M, Fields, R, Jackson A “Anti-Racism and Race Literacy: A Primer and Toolkit for Medical Educators.” Differences Matter at UCSF; 2020. https://ucsf.app.box.com/s/27h19kd597ii66473parki15u0cgochd.

## Slide 39
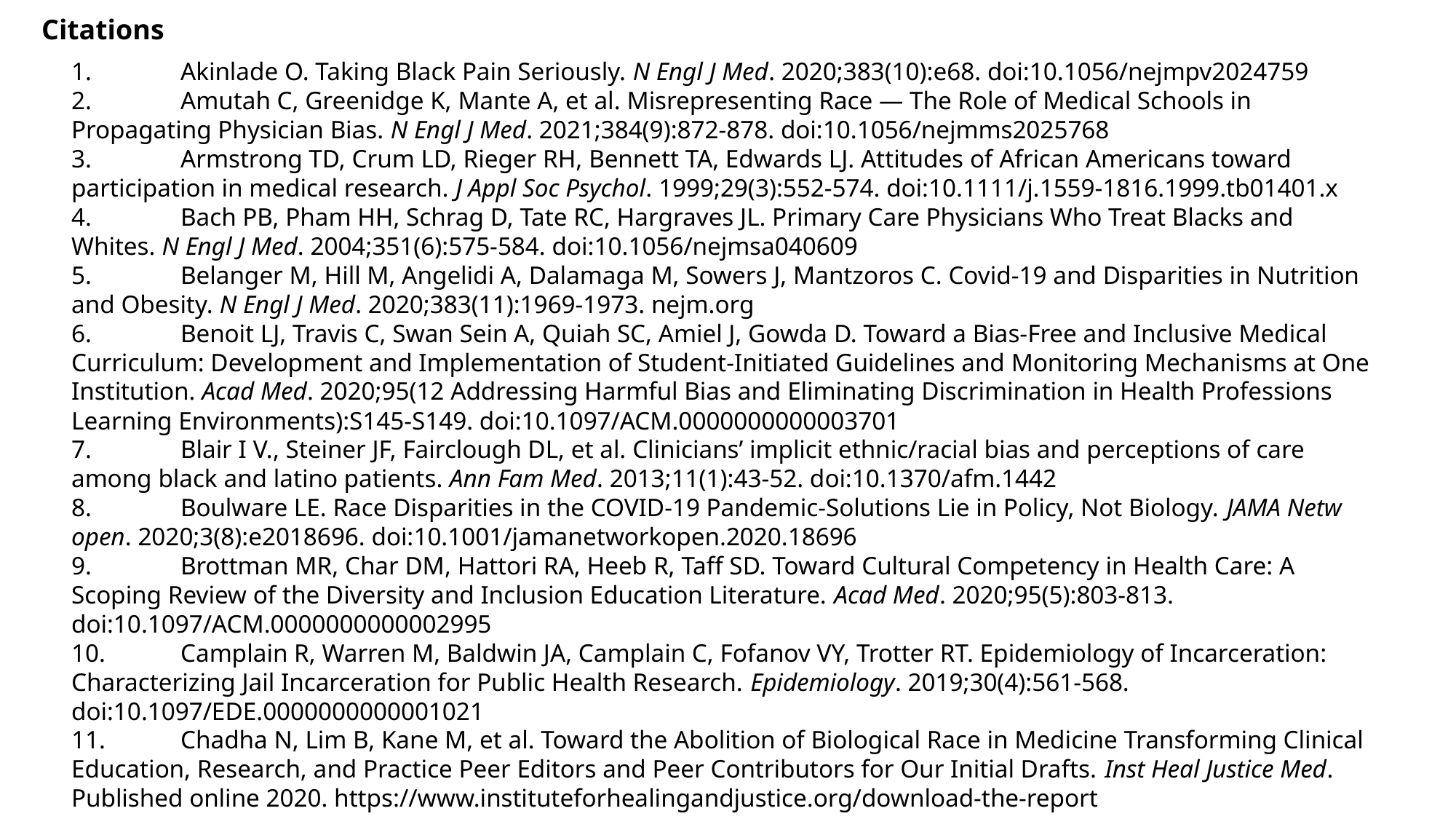

Citations
1. 	Akinlade O. Taking Black Pain Seriously. N Engl J Med. 2020;383(10):e68. doi:10.1056/nejmpv2024759
2. 	Amutah C, Greenidge K, Mante A, et al. Misrepresenting Race — The Role of Medical Schools in Propagating Physician Bias. N Engl J Med. 2021;384(9):872-878. doi:10.1056/nejmms2025768
3. 	Armstrong TD, Crum LD, Rieger RH, Bennett TA, Edwards LJ. Attitudes of African Americans toward participation in medical research. J Appl Soc Psychol. 1999;29(3):552-574. doi:10.1111/j.1559-1816.1999.tb01401.x
4. 	Bach PB, Pham HH, Schrag D, Tate RC, Hargraves JL. Primary Care Physicians Who Treat Blacks and Whites. N Engl J Med. 2004;351(6):575-584. doi:10.1056/nejmsa040609
5. 	Belanger M, Hill M, Angelidi A, Dalamaga M, Sowers J, Mantzoros C. Covid-19 and Disparities in Nutrition and Obesity. N Engl J Med. 2020;383(11):1969-1973. nejm.org
6. 	Benoit LJ, Travis C, Swan Sein A, Quiah SC, Amiel J, Gowda D. Toward a Bias-Free and Inclusive Medical Curriculum: Development and Implementation of Student-Initiated Guidelines and Monitoring Mechanisms at One Institution. Acad Med. 2020;95(12 Addressing Harmful Bias and Eliminating Discrimination in Health Professions Learning Environments):S145-S149. doi:10.1097/ACM.0000000000003701
7. 	Blair I V., Steiner JF, Fairclough DL, et al. Clinicians’ implicit ethnic/racial bias and perceptions of care among black and latino patients. Ann Fam Med. 2013;11(1):43-52. doi:10.1370/afm.1442
8. 	Boulware LE. Race Disparities in the COVID-19 Pandemic-Solutions Lie in Policy, Not Biology. JAMA Netw open. 2020;3(8):e2018696. doi:10.1001/jamanetworkopen.2020.18696
9. 	Brottman MR, Char DM, Hattori RA, Heeb R, Taff SD. Toward Cultural Competency in Health Care: A Scoping Review of the Diversity and Inclusion Education Literature. Acad Med. 2020;95(5):803-813. doi:10.1097/ACM.0000000000002995
10. 	Camplain R, Warren M, Baldwin JA, Camplain C, Fofanov VY, Trotter RT. Epidemiology of Incarceration: Characterizing Jail Incarceration for Public Health Research. Epidemiology. 2019;30(4):561-568. doi:10.1097/EDE.0000000000001021
11. 	Chadha N, Lim B, Kane M, et al. Toward the Abolition of Biological Race in Medicine Transforming Clinical Education, Research, and Practice Peer Editors and Peer Contributors for Our Initial Drafts. Inst Heal Justice Med. Published online 2020. https://www.instituteforhealingandjustice.org/download-the-report

## Slide 40
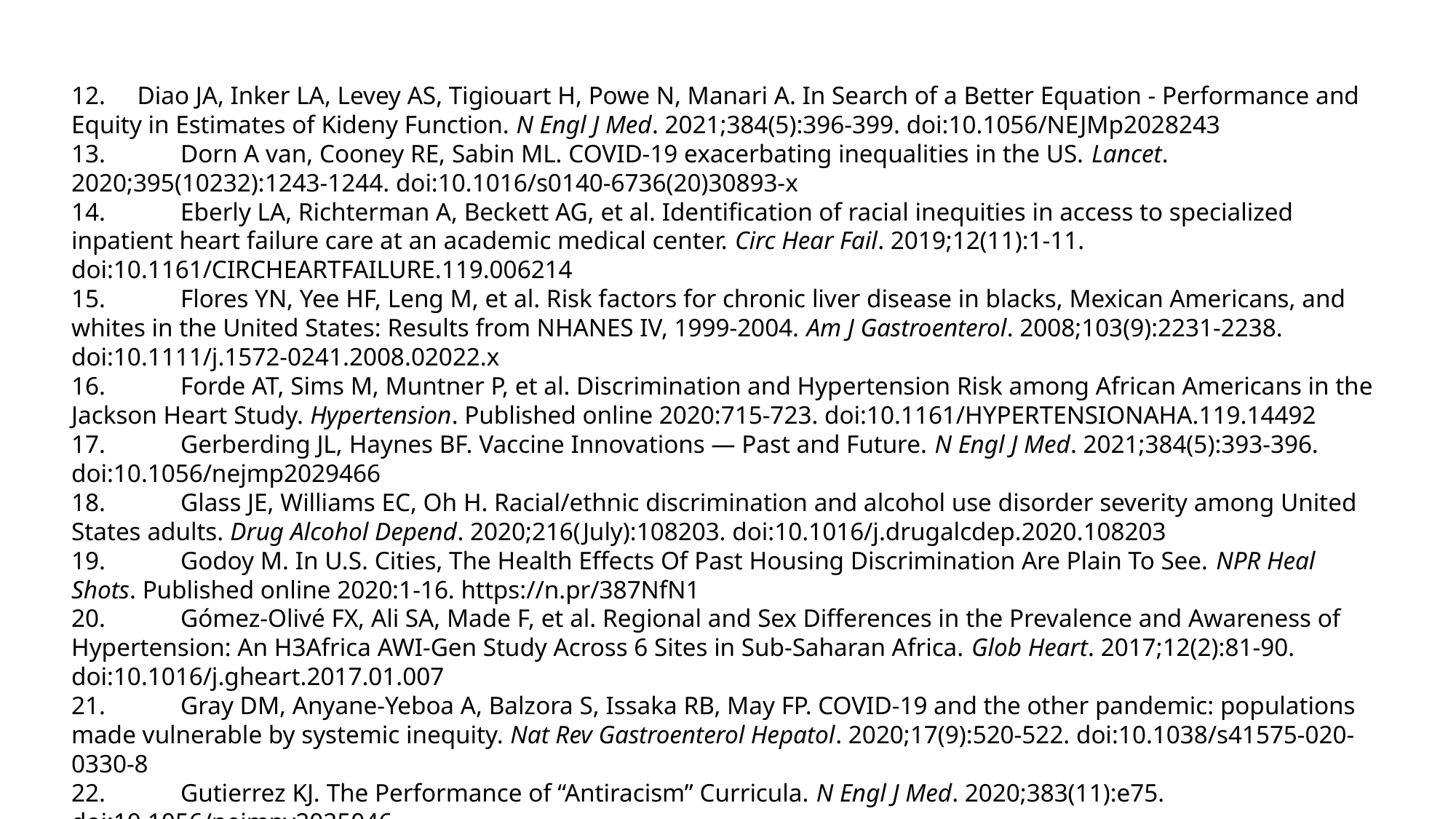

12. Diao JA, Inker LA, Levey AS, Tigiouart H, Powe N, Manari A. In Search of a Better Equation - Performance and Equity in Estimates of Kideny Function. N Engl J Med. 2021;384(5):396-399. doi:10.1056/NEJMp2028243
13. 	Dorn A van, Cooney RE, Sabin ML. COVID-19 exacerbating inequalities in the US. Lancet. 2020;395(10232):1243-1244. doi:10.1016/s0140-6736(20)30893-x
14. 	Eberly LA, Richterman A, Beckett AG, et al. Identification of racial inequities in access to specialized inpatient heart failure care at an academic medical center. Circ Hear Fail. 2019;12(11):1-11. doi:10.1161/CIRCHEARTFAILURE.119.006214
15. 	Flores YN, Yee HF, Leng M, et al. Risk factors for chronic liver disease in blacks, Mexican Americans, and whites in the United States: Results from NHANES IV, 1999-2004. Am J Gastroenterol. 2008;103(9):2231-2238. doi:10.1111/j.1572-0241.2008.02022.x
16. 	Forde AT, Sims M, Muntner P, et al. Discrimination and Hypertension Risk among African Americans in the Jackson Heart Study. Hypertension. Published online 2020:715-723. doi:10.1161/HYPERTENSIONAHA.119.14492
17. 	Gerberding JL, Haynes BF. Vaccine Innovations — Past and Future. N Engl J Med. 2021;384(5):393-396. doi:10.1056/nejmp2029466
18. 	Glass JE, Williams EC, Oh H. Racial/ethnic discrimination and alcohol use disorder severity among United States adults. Drug Alcohol Depend. 2020;216(July):108203. doi:10.1016/j.drugalcdep.2020.108203
19. 	Godoy M. In U.S. Cities, The Health Effects Of Past Housing Discrimination Are Plain To See. NPR Heal Shots. Published online 2020:1-16. https://n.pr/387NfN1
20. 	Gómez-Olivé FX, Ali SA, Made F, et al. Regional and Sex Differences in the Prevalence and Awareness of Hypertension: An H3Africa AWI-Gen Study Across 6 Sites in Sub-Saharan Africa. Glob Heart. 2017;12(2):81-90. doi:10.1016/j.gheart.2017.01.007
21. 	Gray DM, Anyane-Yeboa A, Balzora S, Issaka RB, May FP. COVID-19 and the other pandemic: populations made vulnerable by systemic inequity. Nat Rev Gastroenterol Hepatol. 2020;17(9):520-522. doi:10.1038/s41575-020-0330-8
22. 	Gutierrez KJ. The Performance of “Antiracism” Curricula. N Engl J Med. 2020;383(11):e75. doi:10.1056/nejmpv2025046
23. 	Hawkins D. Differential occupational risk for COVID-19 and other infection exposure according to race and ethnicity. Am J Ind Med. 2020;63(9):817-820. doi:10.1002/ajim.23145

## Slide 41
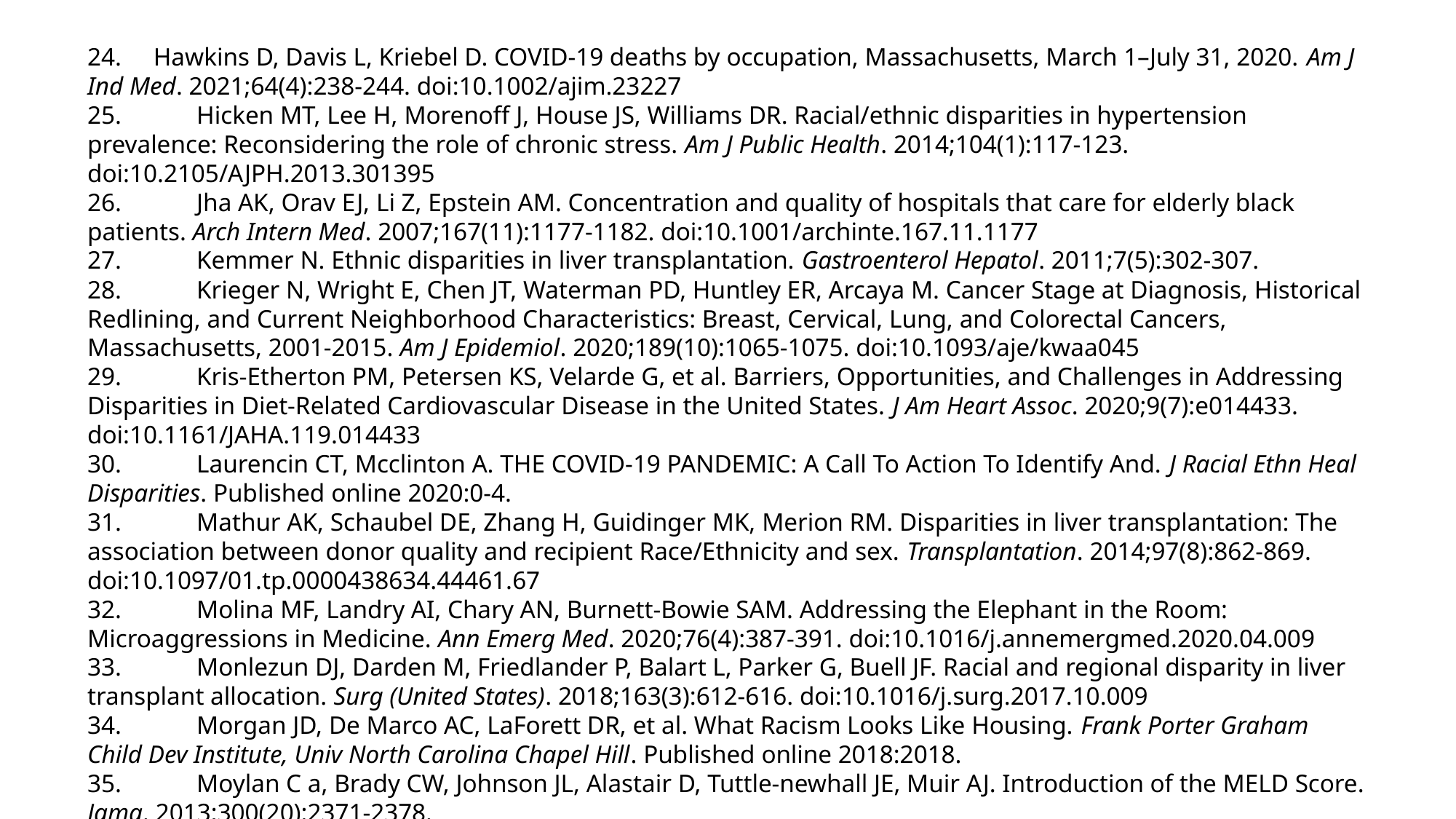

24. Hawkins D, Davis L, Kriebel D. COVID-19 deaths by occupation, Massachusetts, March 1–July 31, 2020. Am J Ind Med. 2021;64(4):238-244. doi:10.1002/ajim.23227
25. 	Hicken MT, Lee H, Morenoff J, House JS, Williams DR. Racial/ethnic disparities in hypertension prevalence: Reconsidering the role of chronic stress. Am J Public Health. 2014;104(1):117-123. doi:10.2105/AJPH.2013.301395
26. 	Jha AK, Orav EJ, Li Z, Epstein AM. Concentration and quality of hospitals that care for elderly black patients. Arch Intern Med. 2007;167(11):1177-1182. doi:10.1001/archinte.167.11.1177
27. 	Kemmer N. Ethnic disparities in liver transplantation. Gastroenterol Hepatol. 2011;7(5):302-307.
28. 	Krieger N, Wright E, Chen JT, Waterman PD, Huntley ER, Arcaya M. Cancer Stage at Diagnosis, Historical Redlining, and Current Neighborhood Characteristics: Breast, Cervical, Lung, and Colorectal Cancers, Massachusetts, 2001-2015. Am J Epidemiol. 2020;189(10):1065-1075. doi:10.1093/aje/kwaa045
29. 	Kris-Etherton PM, Petersen KS, Velarde G, et al. Barriers, Opportunities, and Challenges in Addressing Disparities in Diet-Related Cardiovascular Disease in the United States. J Am Heart Assoc. 2020;9(7):e014433. doi:10.1161/JAHA.119.014433
30. 	Laurencin CT, Mcclinton A. THE COVID-19 PANDEMIC: A Call To Action To Identify And. J Racial Ethn Heal Disparities. Published online 2020:0-4.
31. 	Mathur AK, Schaubel DE, Zhang H, Guidinger MK, Merion RM. Disparities in liver transplantation: The association between donor quality and recipient Race/Ethnicity and sex. Transplantation. 2014;97(8):862-869. doi:10.1097/01.tp.0000438634.44461.67
32. 	Molina MF, Landry AI, Chary AN, Burnett-Bowie SAM. Addressing the Elephant in the Room: Microaggressions in Medicine. Ann Emerg Med. 2020;76(4):387-391. doi:10.1016/j.annemergmed.2020.04.009
33. 	Monlezun DJ, Darden M, Friedlander P, Balart L, Parker G, Buell JF. Racial and regional disparity in liver transplant allocation. Surg (United States). 2018;163(3):612-616. doi:10.1016/j.surg.2017.10.009
34. 	Morgan JD, De Marco AC, LaForett DR, et al. What Racism Looks Like Housing. Frank Porter Graham Child Dev Institute, Univ North Carolina Chapel Hill. Published online 2018:2018.
35. 	Moylan C a, Brady CW, Johnson JL, Alastair D, Tuttle-newhall JE, Muir AJ. Introduction of the MELD Score. Jama. 2013;300(20):2371-2378.

## Slide 42
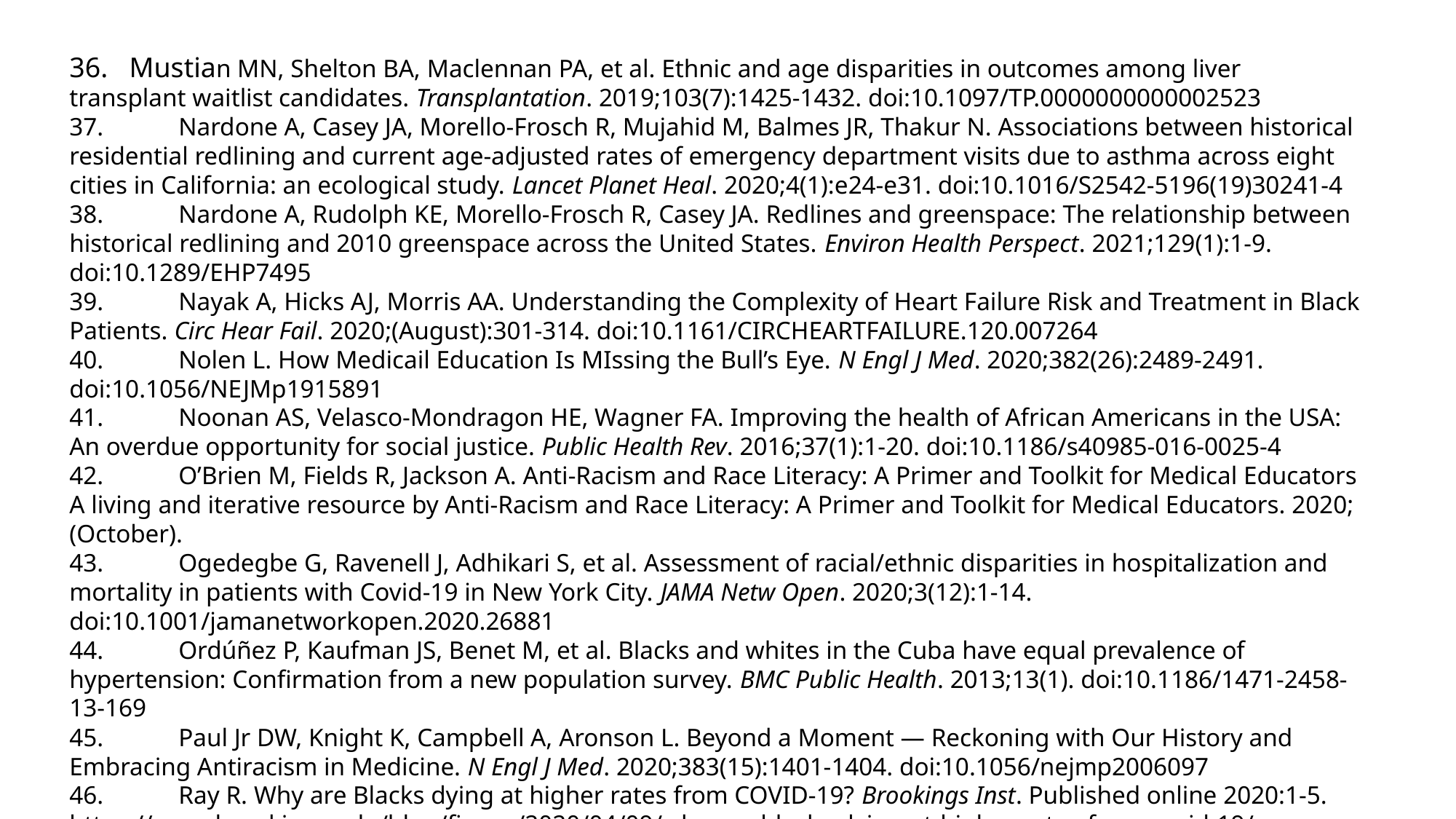

36. Mustian MN, Shelton BA, Maclennan PA, et al. Ethnic and age disparities in outcomes among liver transplant waitlist candidates. Transplantation. 2019;103(7):1425-1432. doi:10.1097/TP.0000000000002523
37. 	Nardone A, Casey JA, Morello-Frosch R, Mujahid M, Balmes JR, Thakur N. Associations between historical residential redlining and current age-adjusted rates of emergency department visits due to asthma across eight cities in California: an ecological study. Lancet Planet Heal. 2020;4(1):e24-e31. doi:10.1016/S2542-5196(19)30241-4
38. 	Nardone A, Rudolph KE, Morello-Frosch R, Casey JA. Redlines and greenspace: The relationship between historical redlining and 2010 greenspace across the United States. Environ Health Perspect. 2021;129(1):1-9. doi:10.1289/EHP7495
39. 	Nayak A, Hicks AJ, Morris AA. Understanding the Complexity of Heart Failure Risk and Treatment in Black Patients. Circ Hear Fail. 2020;(August):301-314. doi:10.1161/CIRCHEARTFAILURE.120.007264
40. 	Nolen L. How Medicail Education Is MIssing the Bull’s Eye. N Engl J Med. 2020;382(26):2489-2491. doi:10.1056/NEJMp1915891
41. 	Noonan AS, Velasco-Mondragon HE, Wagner FA. Improving the health of African Americans in the USA: An overdue opportunity for social justice. Public Health Rev. 2016;37(1):1-20. doi:10.1186/s40985-016-0025-4
42. 	O’Brien M, Fields R, Jackson A. Anti-Racism and Race Literacy: A Primer and Toolkit for Medical Educators A living and iterative resource by Anti-Racism and Race Literacy: A Primer and Toolkit for Medical Educators. 2020;(October).
43. 	Ogedegbe G, Ravenell J, Adhikari S, et al. Assessment of racial/ethnic disparities in hospitalization and mortality in patients with Covid-19 in New York City. JAMA Netw Open. 2020;3(12):1-14. doi:10.1001/jamanetworkopen.2020.26881
44. 	Ordúñez P, Kaufman JS, Benet M, et al. Blacks and whites in the Cuba have equal prevalence of hypertension: Confirmation from a new population survey. BMC Public Health. 2013;13(1). doi:10.1186/1471-2458-13-169
45. 	Paul Jr DW, Knight K, Campbell A, Aronson L. Beyond a Moment — Reckoning with Our History and Embracing Antiracism in Medicine. N Engl J Med. 2020;383(15):1401-1404. doi:10.1056/nejmp2006097
46. 	Ray R. Why are Blacks dying at higher rates from COVID-19? Brookings Inst. Published online 2020:1-5. https://www.brookings.edu/blog/fixgov/2020/04/09/why-are-blacks-dying-at-higher-rates-from-covid-19/

## Slide 43
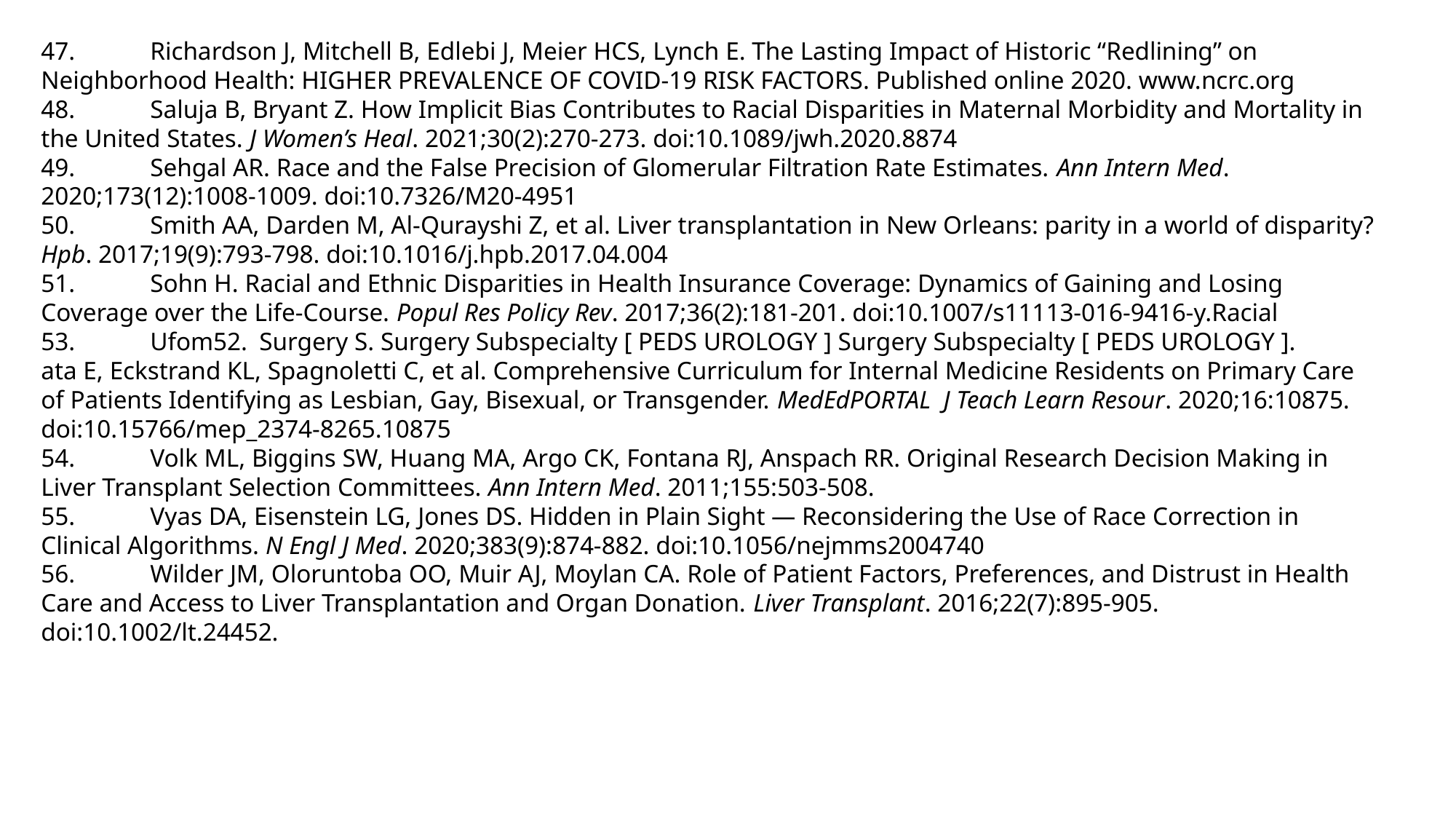

47. 	Richardson J, Mitchell B, Edlebi J, Meier HCS, Lynch E. The Lasting Impact of Historic “Redlining” on Neighborhood Health: HIGHER PREVALENCE OF COVID-19 RISK FACTORS. Published online 2020. www.ncrc.org
48. 	Saluja B, Bryant Z. How Implicit Bias Contributes to Racial Disparities in Maternal Morbidity and Mortality in the United States. J Women’s Heal. 2021;30(2):270-273. doi:10.1089/jwh.2020.8874
49. 	Sehgal AR. Race and the False Precision of Glomerular Filtration Rate Estimates. Ann Intern Med. 2020;173(12):1008-1009. doi:10.7326/M20-4951
50. 	Smith AA, Darden M, Al-Qurayshi Z, et al. Liver transplantation in New Orleans: parity in a world of disparity? Hpb. 2017;19(9):793-798. doi:10.1016/j.hpb.2017.04.004
51. 	Sohn H. Racial and Ethnic Disparities in Health Insurance Coverage: Dynamics of Gaining and Losing Coverage over the Life-Course. Popul Res Policy Rev. 2017;36(2):181-201. doi:10.1007/s11113-016-9416-y.Racial
53. 	Ufom52. 	Surgery S. Surgery Subspecialty [ PEDS UROLOGY ] Surgery Subspecialty [ PEDS UROLOGY ].
ata E, Eckstrand KL, Spagnoletti C, et al. Comprehensive Curriculum for Internal Medicine Residents on Primary Care of Patients Identifying as Lesbian, Gay, Bisexual, or Transgender. MedEdPORTAL  J Teach Learn Resour. 2020;16:10875. doi:10.15766/mep_2374-8265.10875
54. 	Volk ML, Biggins SW, Huang MA, Argo CK, Fontana RJ, Anspach RR. Original Research Decision Making in Liver Transplant Selection Committees. Ann Intern Med. 2011;155:503-508.
55. 	Vyas DA, Eisenstein LG, Jones DS. Hidden in Plain Sight — Reconsidering the Use of Race Correction in Clinical Algorithms. N Engl J Med. 2020;383(9):874-882. doi:10.1056/nejmms2004740
56. 	Wilder JM, Oloruntoba OO, Muir AJ, Moylan CA. Role of Patient Factors, Preferences, and Distrust in Health Care and Access to Liver Transplantation and Organ Donation. Liver Transplant. 2016;22(7):895-905. doi:10.1002/lt.24452.
