# Supplementary material for: DARE Training: Teaching Educators How to Revise Internal Medicine Residency Lectures by Using an Anti-racism Framework
Source: MedEdPORTAL. 2023 Nov 7;19:11351. doi: 10.15766/mep_2374-8265.11351 (PMC10627787; doi:10.15766/mep_2374-8265.11351)
Supplement: Supplementary file 1 — DARE Checklist of Best Practices.pptxPreworkshop Intro Facilitator Guide.docxPreworkshop Intro Slides.pptxWorkshop Facilitator Guide.docxWorkshop Slides.pptxPretraining Assessment.pptxPosttraining Assessment.pptxDARE Rubric.docxDARE Training Timeline.pptx [file mep_2374-8265.11351-s001.zip › F. Pretraining Assessment.pptx]

## Slide 1
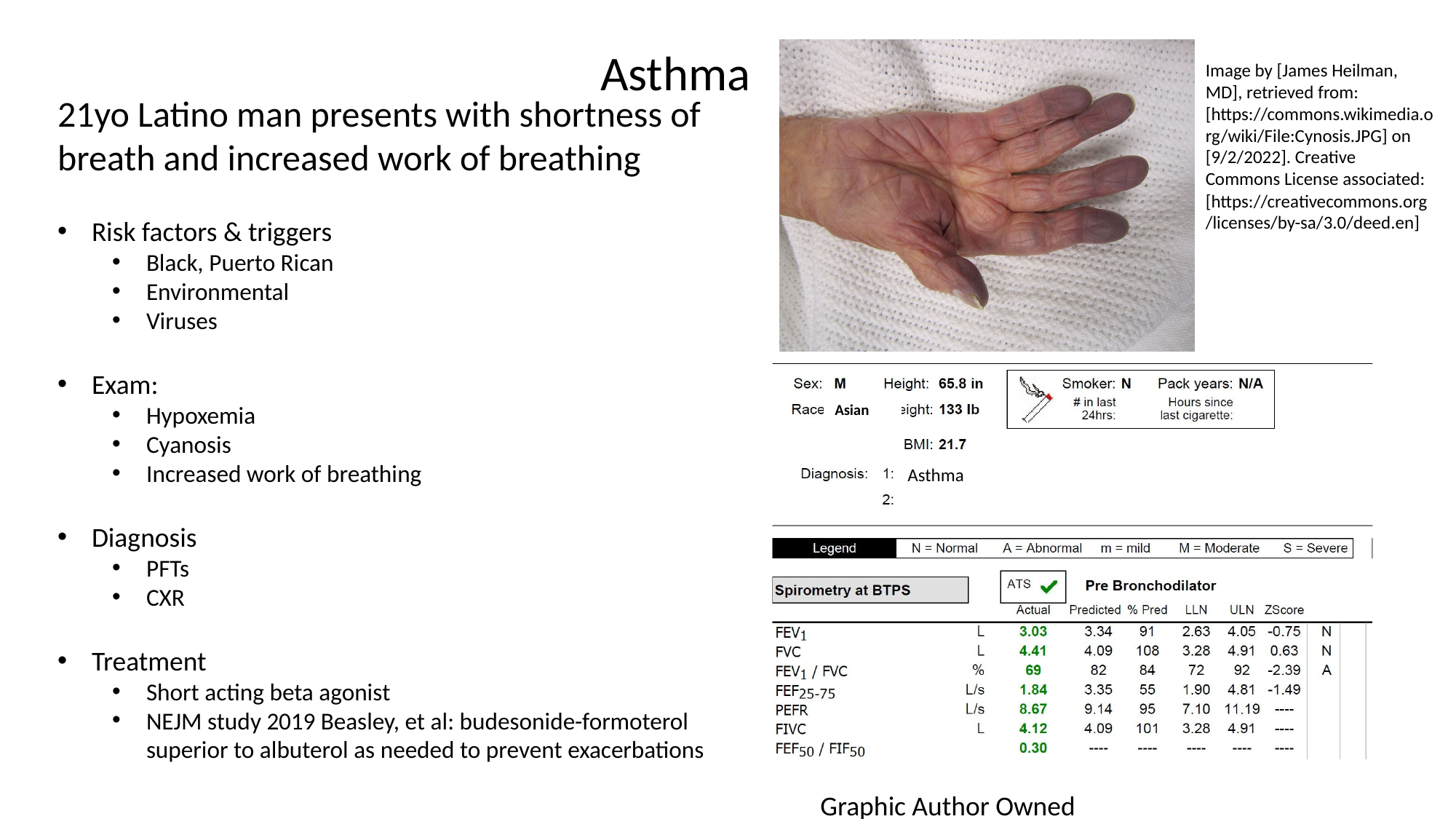

Asthma
Image by [James Heilman, MD], retrieved from: [https://commons.wikimedia.org/wiki/File:Cynosis.JPG] on [9/2/2022]. Creative Commons License associated: [https://creativecommons.org/licenses/by-sa/3.0/deed.en]
21yo Latino man presents with shortness of breath and increased work of breathing
Risk factors & triggers
Black, Puerto Rican
Environmental
Viruses
Exam:
Hypoxemia
Cyanosis
Increased work of breathing
Diagnosis
PFTs
CXR
Treatment
Short acting beta agonist
NEJM study 2019 Beasley, et al: budesonide-formoterol superior to albuterol as needed to prevent exacerbations
Asian
Asthma
Graphic Author Owned
