# Supplementary material for: DARE Training: Teaching Educators How to Revise Internal Medicine Residency Lectures by Using an Anti-racism Framework
Source: MedEdPORTAL. 2023 Nov 7;19:11351. doi: 10.15766/mep_2374-8265.11351 (PMC10627787; doi:10.15766/mep_2374-8265.11351)
Supplement: Supplementary file 1 — DARE Checklist of Best Practices.pptxPreworkshop Intro Facilitator Guide.docxPreworkshop Intro Slides.pptxWorkshop Facilitator Guide.docxWorkshop Slides.pptxPretraining Assessment.pptxPosttraining Assessment.pptxDARE Rubric.docxDARE Training Timeline.pptx [file mep_2374-8265.11351-s001.zip › G. Posttraining Assessment.pptx]

## Slide 1
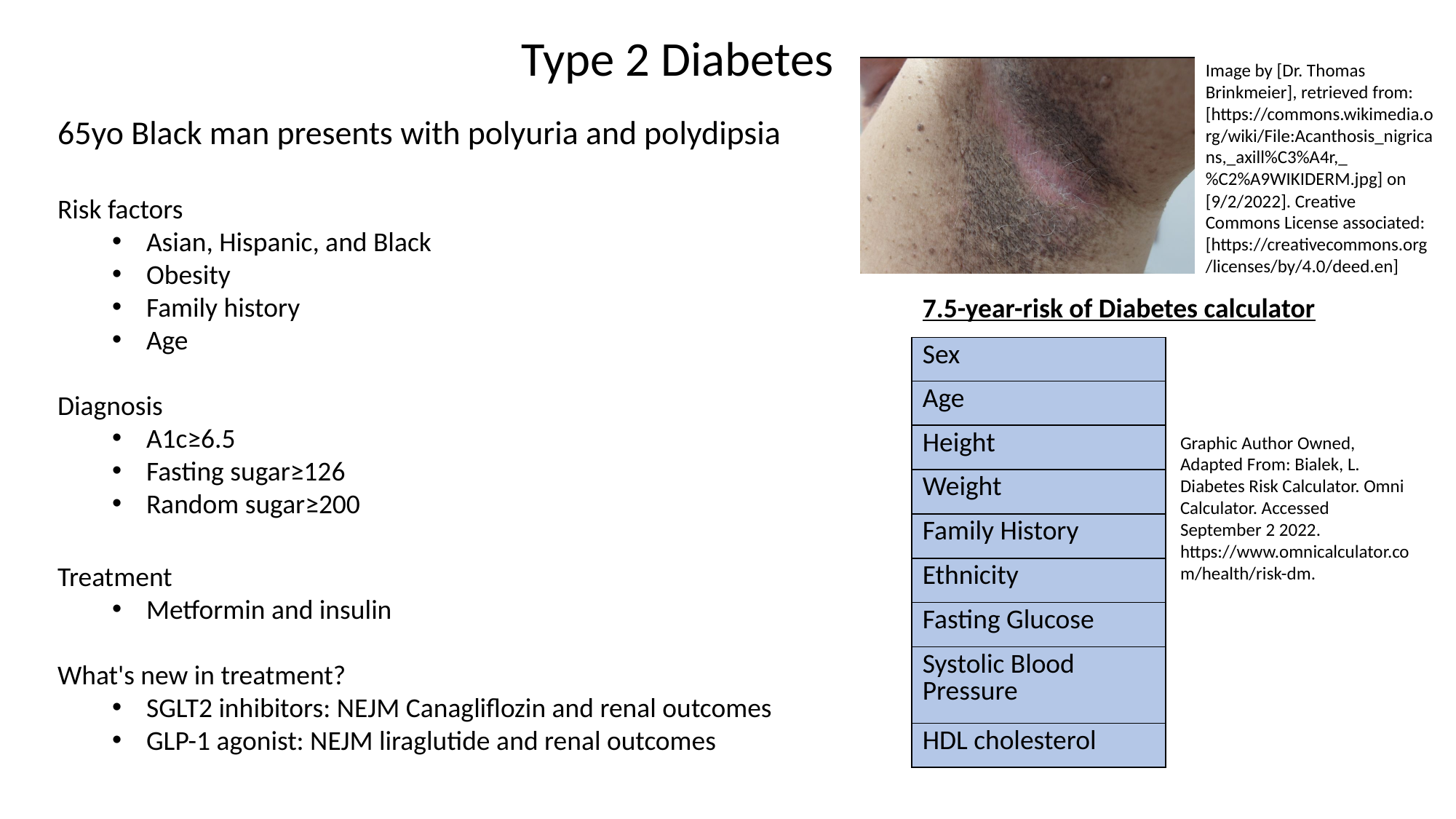

Type 2 Diabetes
Image by [Dr. Thomas Brinkmeier], retrieved from: [https://commons.wikimedia.org/wiki/File:Acanthosis_nigricans,_axill%C3%A4r,_%C2%A9WIKIDERM.jpg] on [9/2/2022]. Creative Commons License associated: [https://creativecommons.org/licenses/by/4.0/deed.en]
65yo Black man presents with polyuria and polydipsia
Risk factors
Asian, Hispanic, and Black
Obesity
Family history
Age
Diagnosis
A1c≥6.5
Fasting sugar≥126
Random sugar≥200
Treatment
Metformin and insulin
What's new in treatment?
SGLT2 inhibitors: NEJM Canagliflozin and renal outcomes
GLP-1 agonist: NEJM liraglutide and renal outcomes
7.5-year-risk of Diabetes calculator
| Sex |
| --- |
| Age |
| Height |
| Weight |
| Family History |
| Ethnicity |
| Fasting Glucose |
| Systolic Blood Pressure |
| HDL cholesterol |
Graphic Author Owned, Adapted From: Bialek, L. Diabetes Risk Calculator. Omni Calculator. Accessed September 2 2022. https://www.omnicalculator.com/health/risk-dm.
